# Transdiagnostic and Disorder-Level Genome-Wide Association Studies Enhance Precision of Substance Use and Psychiatric Genetic Risk Profiles in African and European Ancestries

**DOI:** 10.1016/j.biopsych.2025.04.021

**Published:** 2025-05-08

**Authors:** Yousef Khan, Christal N. Davis, Zeal Jinwala, Kyra L. Feuer, Sylvanus Toikumo, Emily E. Hartwell, Sandra Sanchez-Roige, Roseann E. Peterson, Alexander S. Hatoum, Henry R. Kranzler, Rachel L. Kember

**Affiliations:** Department of Psychiatry, University of Pennsylvania School of Medicine, Philadelphia, Pennsylvania (YK, CND, ZJ, KLF, ST, EEH, HRK, RLK); Mental Illness Research, Education and Clinical Center, Crescenz Veterans Affairs Medical Center, Philadelphia, Pennsylvania (CND, ZJ, ST, EEH, HRK, RLK); Department of Psychiatry, University of California San Diego, La Jolla, California (SSR); Division of Genetic Medicine, Vanderbilt University Medical Center, Nashville, Tennessee (SS-R); Genomic Medicine, University of California San Diego, La Jolla, California (SS-R); Institute for Department of Psychiatry and Behavioral Sciences, Institute for Genomics in Health, SUNY Downstate Health Sciences University, Brooklyn, New York (REP); and Department of Psychological & Brain Sciences, Washington University in St. Louis, St. Louis, Missouri (ASH).

## Abstract

**BACKGROUND:**

Substance use disorders (SUDs) and psychiatric disorders frequently co-occur, and their etiology likely reflects both transdiagnostic (i.e., common/shared) and disorder-level (i.e., independent/nonshared) genetic influences. Understanding the genetic influences that are shared and those that operate independently of the shared risk could enhance precision in diagnosis, prevention, and treatment, but this remains underexplored, particularly in non-European ancestry groups.

**METHODS:**

We applied genomic structural equation modeling to examine the common and independent genetic architecture among SUDs and psychotic, mood, and anxiety disorders using summary statistics from genome-wide association studies (GWASs) conducted in European ancestry (EUR) and African ancestry (AFR) individuals. To characterize the biological and phenotypic associations, we used FUMA, conducted genetic correlations, and performed phenome-wide association studies (PheWASs).

**RESULTS:**

In EUR individuals, transdiagnostic genetic factors represented SUDs, psychotic disorders, and mood/anxiety disorders, with a GWAS identifying 2 novel lead single nucleotide polymorphisms (SNPs) for the mood factor. In AFR individuals, genetic factors represented SUDs and psychiatric disorders, and a GWAS identified 1 novel lead SNP for the SUD factor. In EUR individuals, second-order factor models showed phenotypic and genotypic associations with a broad range of physical and mental health traits. Finally, genetic correlations and PheWASs highlighted how common and independent genetic factors for SUDs and psychotic disorders were differentially associated with psychiatric, sociodemographic, and medical phenotypes.

**CONCLUSIONS:**

Combining transdiagnostic and disorder-level genetic approaches can improve our understanding of co-occurring conditions and increase the specificity of genetic discovery, which is critical for identifying more effective prevention and treatment strategies to reduce the burden of these disorders.

Substance use disorders (SUDs) commonly co-occur with mood, anxiety (ANX), and psychotic disorders (1-3), which complicates the clinical course of affected individuals and results in greater health care and other costs (4,5). Genomewide association studies (GWASs) have shown these disorders to be highly polygenic and pleiotropic, with pleiotropic effects partially accounting for the co-occurrence of psychiatric disorders (6-13). Given this extensive pleiotropy, methods that model the shared structure of psychiatric disorders can provide deeper insights into their underlying biology. Genomic structural equation modeling (gSEM) allows this approach, leveraging shared liability to identify genetic factors that underlie multiple disorders (14) and improving our understanding of the shared biological pathways and risk mechanisms of commonly co-occurring conditions.

Several gSEM studies have modeled pleiotropy across psychiatric disorders in European ancestry (EUR) individuals and variously identified mood/internalizing, psychotic, neurodevelopmental, and compulsive factors (14-16). Similarly, an addiction factor was identified that underlies cannabis use disorder (CanUD), opioid use disorder (OUD), and problematic alcohol and tobacco use (17). To date, studies have applied gSEM only in EUR individuals, because modeling complex genetic relationships via SEM imposes greater demands for statistical power than simpler univariate GWASs, which themselves are often underpowered in non-EUR groups. Although the increasing inclusion of African ancestry (AFR) individuals in GWASs is beginning to mitigate this issue, low statistical power still precludes the application of gSEM in other ancestral groups.

Although previous studies typically focused on identifying transdiagnostic genetic risk, gSEM also enables more precise identification of disorder-specific genetic mechanisms than individual GWASs. Specifically, GWAS-by-subtraction (18) is a method that can parse genetic associations into those that influence risk for a disorder through a common genetic factor and those that operate independently of the common factor. Combining transdiagnostic and disorder-level approaches enhances statistical power to detect pleiotropic effects that contribute to transdiagnostic risk while also identifying patterns of genetic heterogeneity and increasing the specificity of genetic discovery (19).

To extend previous findings, we used gSEM to characterize the genetic structure shared among SUDs and psychotic, mood, and ANX disorders in EUR and, for the first time, AFR individuals. We first examined the disorders’ genetic factor structure and then explored each factor’s biological underpinnings by conducting GWASs. Next, we investigated the shared genetic etiology between SUDs and classes of psychiatric disorders by identifying second-order common factors. Finally, to enhance specificity, we characterized the common and independent genetic variance for select well-powered disorders using GWAS-by-subtraction. Thus, we addressed 2 critical gaps in previous research by applying gSEM among AFR individuals and by using a hierarchical approach to explore genetic specificity and transdiagnostic genetic risk.

## METHODS AND MATERIALS

### Preparation of Summary Statistics

We obtained summary statistics from large GWASs for SUDs and psychotic, mood, and ANX disorders in EUR and AFR individuals (Table 1 and Tables S1-S4). Genetic ancestry inferences, determined in the original studies, represent statistical approximations of genetic similarity and are not proxies for race or ethnicity. The traits included 4 SUDs: alcohol use disorder (AUD) (11,20), tobacco use disorder (TUD) (13), CanUD (10), and OUD (9); 2 disorders that can include psychotic features: bipolar disorder (BD) (8,21) and schizophrenia (SCZ) (7,21); and 2 mood and ANX traits: ANX (6,22,23) and major depressive disorder (MDD) (12,24). In EUR individuals, we used multitrait analysis of GWAS (MTAG) (25) to improve our ability to detect effects associated with a broad spectrum of ANX disorders (Supplemental Methods and Table S5).

In preparing summary statistics for gSEM, we performed linkage disequilibrium score regression (LDSC) as a necessary preliminary step, as gSEM leverages the genetic correlations among traits. To ensure optimal model performance, we tested 3 sets of LDSC reference panels in AFR individuals (Supplemental Methods). Ultimately, we selected the 1000 Genomes phase 3 reference panel (26) for EUR individuals and the Pan-UK Biobank panel (27) for AFR individuals, as these panels provided the most reliable genetic correlation estimates for gSEM. We used the reference panels to calculate genetic correlations between the included disorders separately in EUR and AFR individuals prior to gSEM (Supplemental Methods).

### Genomic SEM

**Figure 1.**
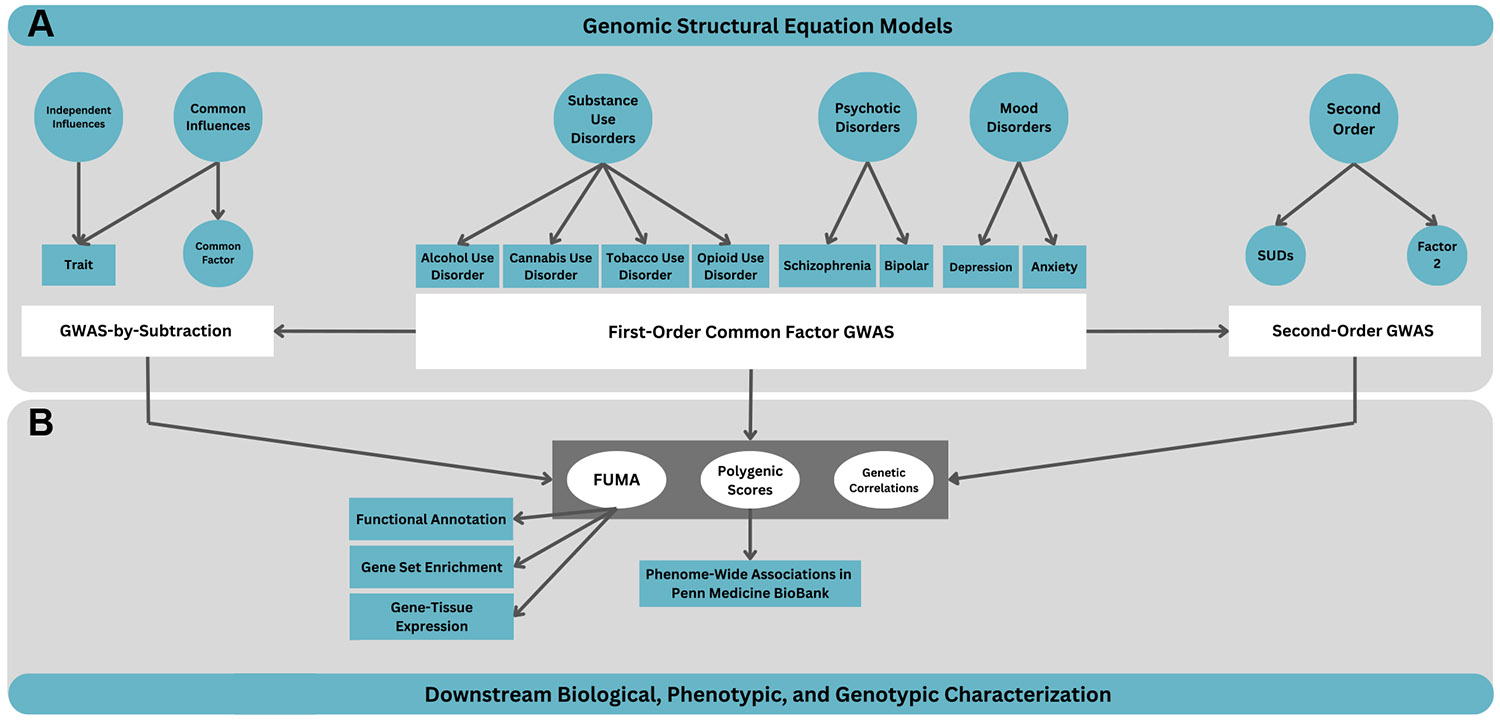
Study schema. Panel **(A)** (top half) depicts the genomic structural equation modeling process. This includes specifying first-order and second-order common factor models, conducting GWASs on the common factors, and performing GWAS-by-subtraction. Panel **(B)** (bottom half) outlines the downstream analyses used to characterize results from the genomic structural equation modeling analyses in **(A)**. FUMA was used for biological characterization, while polygenic scores and genetic correlations were used to evaluate phenotypic and genetic associations, respectively. GWAS, genome-wide association study; SUD, substance use disorder.

An overview of our modeling approach and downstream analyses is detailed below and represented in Figure 1. First, we specified first-order common factors by performing exploratory factor analysis (EFA) and confirmatory factor analysis (CFA) on independent data (odd vs. even chromosomes), and then we evaluated fit, theoretical consistency, and parsimony to determine the optimal model (Supplemental Methods). Each first-order common factor represents the genetic liability shared across disorders within a similar class (e.g., SUDs). To capture the shared genetic liability across SUDs and other psychiatric disorder classes, we specified second-order common factors, which model the genetic variance shared among the first-order common factors (Supplemental Methods). To estimate the single nucleotide polymorphism (SNP) associations with each first- and second-order genetic factor, we conducted GWASs using diagonally weighted least squares estimation (Supplemental Methods). We calculated the effective sample size of each factor as described by Mallard *et al*. (28).

To mitigate the effect of heterogeneous SNPs on both firstand second-order GWASs, we removed SNPs with a *Q*_SNP_ *p* value < 5 × 10^−28^ (Supplemental Methods, Figure S1, and Tables S6-S10). To identify significant independent SNPs, we performed LD clumping using PLINK 1.9 (29) (Supplemental Methods). For loci not previously associated with a corresponding SUD or psychotic, mood, or ANX disorder (hereafter referred to as novel), we performed a phenome-wide association study (PheWAS) on the lead SNP using GWAS Atlas (30) (Supplemental Methods) and used the LD-based PICS version 2.1.1 finemapping tool to assess the most likely causal variant responsible for the association in a locus.

Lastly, we applied GWAS-by-subtraction (18), a method based on gSEM, to the first-order models to investigate genetic influences on each trait, distinguishing those operating through the common factor from those operating independently of it (Figure S2). To ensure that GWAS-by-subtraction models were informative and adequately powered, we performed them only on disorders with a standardized unexplained variance >0.30 in the first-order CFA. Due to the limited statistical power of the AFR models, we restricted GWAS-by-subtraction to EUR individuals. Effective sample size calculations for GWAS-by-subtraction models were adjusted to account for the fact that the GWASs modeled residual heritability (18).

### Downstream Analyses

#### Biological Characterization.

We used FUMA version 1.6.0 (31) and MAGMA version 1.08 (32) to conduct downstream analyses, including gene-based tests, gene-set enrichment, and gene-tissue expression analyses in Brain-Span (33) and GTEx version 8 (34) tissue samples. Then, we examined SNP-to-gene associations via 1) expression quantitative trait loci (eQTLs) from PsychENCODE (35) and GTEx version 8 (34) brain tissue data and 2) chromatin interactions via Hi-C data (36). Lastly, we investigated protein-protein interactions (PPIs) of MAGMA-identified genes using STRING version 12.0 (37) to identify whether proteins encoded by these genes participate in common pathways (Supplemental Methods).

#### Genetic Correlations and Polygenic Score–Based PheWASs.

To characterize disorder-level and transdiagnostic genetic associations with other traits, we calculated genetic correlations using the Complex Traits Genetics Virtual Lab (CTG-VL) (38) and LDSC (39,40) with 1000 Genomes phase 3 (26) (for EUR) and Pan-UK Biobank (27) (for AFR) data as LD references (Supplemental Methods). We calculated transancestry genetic correlations to compare the genetic underpinnings of common factors identified in EUR and AFR individuals using Popcorn version 1.0 (41). To identify how polygenic risk was associated with a broad range of phenotypes, we performed polygenic score (PGS)–based PheWASs in the Penn Medicine Biobank (PMBB) using PRS-CS (42) and the *PheWAS* package in R (43). Genotyping, imputation, and phenotyping procedures were performed as previously described (9,44) (Supplemental Methods).

## RESULTS

### Preparation of Summary Statistics

In EUR individuals, performing MTAG on the ANX GWAS increased the number of genome-wide significant (GWS) lead SNPs from 1 to 5, and the sample size increased from 17,310 to 115,651 (Supplemental Results and Figure S3). In EUR individuals, all the disorders included in the gSEM models were significantly genetically correlated (Supplemental Results, Figure S4, and Table S11), with the strongest correlations among disorders of the same class. In AFR individuals, disorders were significantly genetically correlated within classes (Supplemental Results, Figure S5, and Table S12).

### First-Order Common Factors

#### European Ancestry.

**Figure 2.**
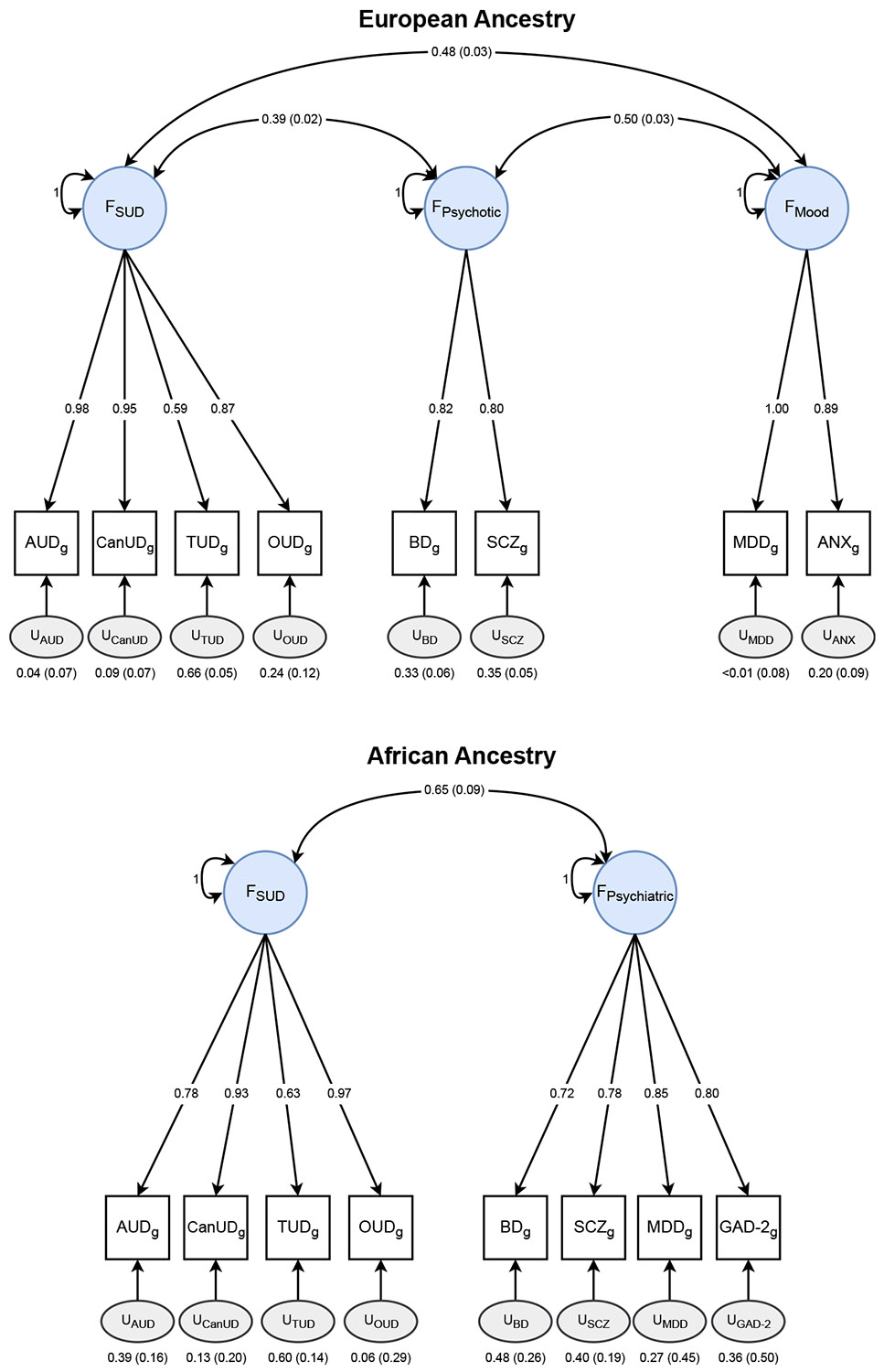
Genomic structural equation models. These pathways depict results from the confirmatory factor analysis of the first-order common factor genomic structural equation modeling. Common factors (blue circles) represent the shared genetic architecture across traits (white squares). Arrows from common factors to specific traits indicate the standardized loading of that trait onto the common factor. Underneath each trait is the standardized residual variance. Arrows between common factors represent correlations. All values in parentheses represent standard error values. ANX, anxiety; AUD, alcohol use disorder; BD, bipolar disorder; CanUD, cannabis use disorder; GAD-2, 2-item Generalized Anxiety Disorder questionnaire; MDD, major depressive disorder; OUD, opioid use disorder; SCZ, schizophrenia; TUD, tobacco use disorder.

Of the EFA models, a 3-factor structure was determined to be optimal based on the fit statistics and theoretical considerations (Supplemental Results and Table S13). A CFA based on this structure fit the data well (χ^2^_17_ = 130.04, *p* = 1.84 × 10^−19^, Akaike information criterion [AIC] = 168.04, comparative fit index [CFI] = 0.95, standardized root mean squared residual [SRMR] = 0.05). SUDs loaded onto the first factor, BD and SCZ loaded onto the second factor, and MDD and ANX loaded onto the third factor (Figure 2). The factors were significantly intercorrelated (Table S14).

**Figure 3.**
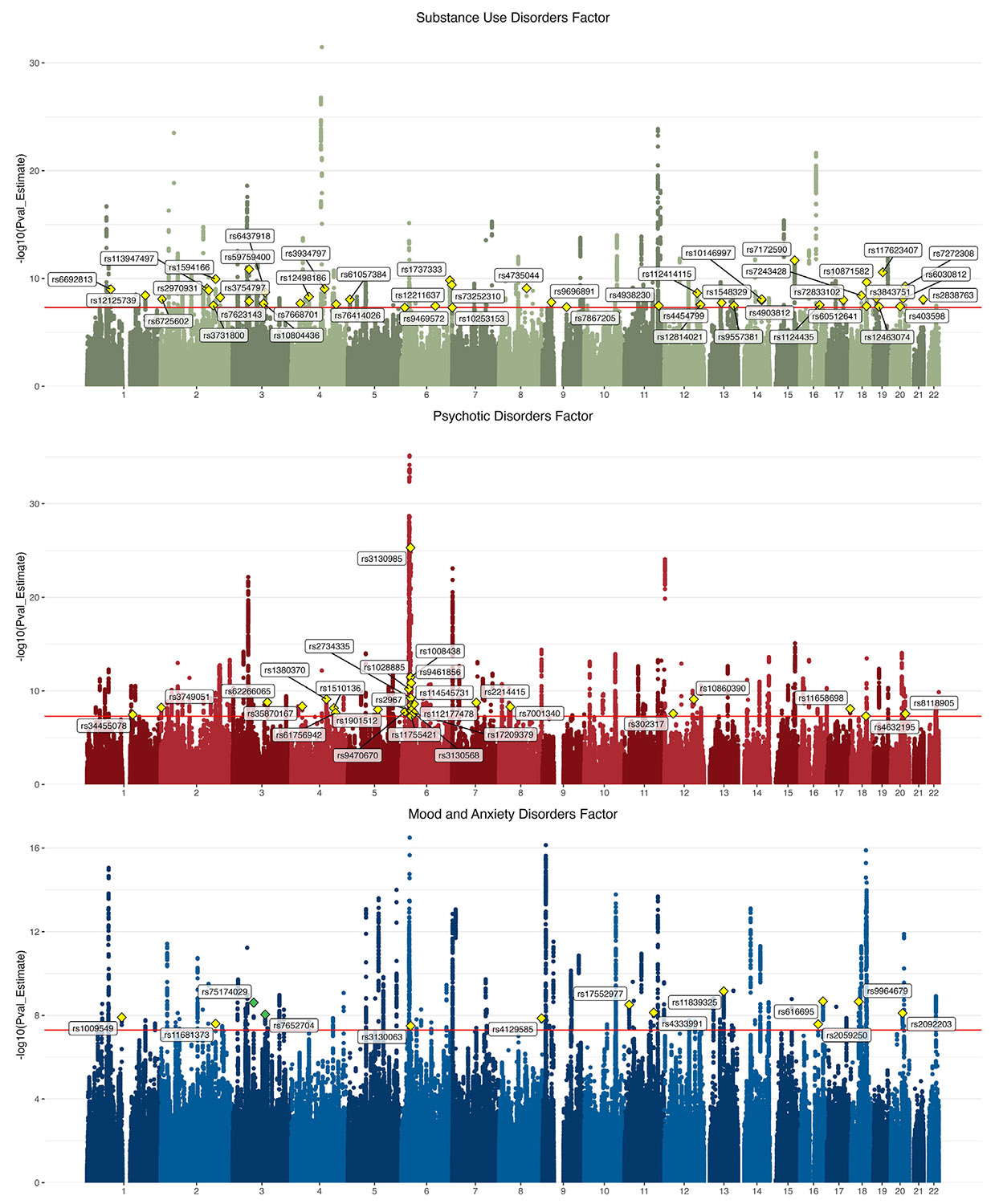
Manhattan plots for common factor GWASs in European ancestry individuals. For GWASs, regression models were conducted between SNPs and each first-order common factor depicted in Figure 2. The substance use disorder factor GWAS identified 143 lead SNPs, the psychotic disorder factor identified 162 lead SNPs, and the mood/anxiety disorder factor identified 112 lead SNPs. The lead SNPs for loci that were not significant in the input GWAS are annotated with yellow diamonds, and lead SNPs for loci not previously significantly associated with phenotypes related to the common factor (i.e., novel) are annotated with green diamonds. Results of the first-order common factor GWAS in African ancestry individuals are shown in Figures S18 and S19. GWAS, genome-wide association study; SNP, single nucleotide polymorphism.

A GWAS of the SUD factor identified 143 lead SNPs (Table S15), 47 of which were not identified (i.e., not GWS or in LD with GWS SNPs) in any of the input SUD GWASs (45). A GWAS of the psychotic disorder factor identified 162 lead SNPs (Table S16), 27 of which had not been identified by the SCZ or BD GWASs. The mood factor GWAS identified 112 lead SNPs (Table S17), 13 of which were not identified by the MDD or ANX GWASs, and 2 (rs75174029 and rs7652704) that were in novel loci for mood or ANX disorders. GWAS results are shown in Figure 3.

The PheWAS of rs75174029, one of the two novel lead SNPs, using GWAS Atlas identified associations with the number of non–cancer-related illnesses, general risk tolerance, and having trouble relaxing. Finemapping identified rs75174029 as the most likely causal variant within the locus (Table S18), and Hi-C data (Figure S6) revealed contact with *FOXP1*, a key regulatory gene in neural development (46,47) (Supplemental Results). A PheWAS of the other novel lead SNP associated rs7652704 with sensitivity/hurt feelings, neuroticism, and positive affect, among others (Figure S7). Although finemapping results for this SNP were inconclusive (Table S19), eQTL mapping demonstrated associations with genes responsible for immune and stress responses and cell organization (48-50) (Figure S8 and Supplemental Results).

Gene-tissue expression analyses showed a role for both SUD- and psychotic disorder–related genes during prenatal brain development, but no developmental period was significant for mood disorders (Figures S9-S11). Mapped genes were enriched for PPIs, indicating shared biological functions among disorders of the same class (SUDs = 1.37×, psychotic = 1.40×, and mood/ANX disorders = 1.43 enrichment; *p*s < 1.00 × 10^−16^).

The SUD factor was strongly genetically correlated with smoking and alcohol, depression, and socioeconomic factors (Figure S12). While the psychotic disorder factor correlated most strongly with psychiatric traits such as BD and SCZ, it also exhibited a positive correlation with risk taking and a negative correlation with cognitive performance (Figure S13). Although the mood factor correlated most strongly with depression and ANX, remaining correlations were predominantly with somatic traits such as chronic pain (Figure S14).

In a PGS-based PheWAS, genetic liability for SUDs was associated with TUD, respiratory illnesses, and mood/ANX disorders (Figure S15 and Table S20). Genetic liability for psychotic disorders was associated with psychiatric traits only (Figure S16 and Table S21), and genetic liability for mood disorders was most strongly associated not only with mood and ANX but also with various physical health traits (Figure S17 and Table S22).

#### African Ancestry.

Fit was generally poor for the EFA models (Table S23). Upon examining factor loadings, the proportion of variance explained, and eigenvalues, we proceeded with a 2-factor model representing SUDs and psychiatric disorders (Figure 2 and Supplemental Results). This model fit adequately (χ^2^_19_ = 21.49, *p* =.31, AIC = 55.49, CFI = 0.99, SRMR = 0.10) and required no constraints (Table S24). The SUD factor GWAS (Figure S18) identified 1 lead SNP, rs1944683, within an intergenic region on chromosome 11. The locus, although previously associated with alcohol consumption, tobacco-related traits, OUD, and CanUD in EUR and cross-ancestry studies, is novel for AFR individuals specifically (51-53). No significant variants were identified for psychiatric disorders (Figure S19).

Gene expression for the SUD factor was enriched in brain tissues involved in emotion processing, reward signaling, and cognitive control, including the putamen, amygdala, caudate, and hippocampus (Figure S20). Gene expression for the psychiatric disorder factor was not enriched for any developmental stage or tissue type, although the top associations were with brain tissues (Figure S21).

The psychiatric disorder factor was genetically correlated with all 11 traits examined, and the SUD factor was correlated with all except posttraumatic stress disorder (PTSD) (Figure S22). Although PheWASs identified no significant associations, the top hits generally aligned with the factor being examined. For example, genetic liability for SUDs was most strongly associated with TUD, followed by SUDs broadly (Figure S23 and Table S25). Among the top associations for the psychiatric factor was generalized ANX disorder (Figure S24 and Table S26).

Transancestry genetic correlations showed that the EUR SUD factor was genetically correlated with the AFR SUD factor (*r*_g_ = 0.730, SE = 0.094, *p* = .004). Similarly, the AFR psychiatric disorder factor was genetically correlated with the EUR psychotic disorder (*r*_g_ = 0.471, SE = 0.216, *p* = .014) and mood (*r*_g_ = 0.571, SE = 0.204, *p* = .035) factors.

### Second-Order Common Factors

#### European Ancestry.

The first-order SUD factor was genetically correlated with both the psychotic disorder (*r*_g_ = 0.38, SE = 0.03, *p* < .001) and mood (*r*_g_ = 0.44, SE = 0.03, *p* < .001) factors, so we used a higher-order CFA model (χ^2^_2_ = 57.61, *p* = 3.09 × 10^−13^, AIC = 65.61, CFI = 0.91, SRMR = 0.07) (Figure S25 and Table S27) to examine this shared structure. After accounting for shared genetic risk in the second-order model, there was less residual variance in the first-order SUD (u_SUD_ = 0.19, SE = 0.04) factor than in the psychotic disorder (u_Psychotic_ = 0.63, SE = 0.05) or mood (u_Mood_ = 0.56, SE = 0.04) factors. A GWAS of the second-order SUD and psychotic disorder factor, capturing genetic variance shared among SUDs and psychotic disorders, identified 76 lead SNPs (Figure S26 and Table S28), whereas a GWAS of the second-order SUD and mood factors identified 62 (Figure S27 and Table S29).

Genetic risk shared among SUDs and psychotic disorders implicated gene sets involved in transcription regulation, sequence-specific DNA binding, and neuron differentiation. Genetic risk shared between SUDs and mood disorders was associated with gene sets involved in mechanosensory behavior and axonal protein transport. For both second-order factors, gene expression was enriched in brain tissues (Figures S28 and S29). Mapped genes were enriched for PPIs (SUDs and psychotic disorder = 1.54×, SUDs and mood/ANX disorders = 1.57×; *p*s < 1.00 × 10^−16^).

**Figure 4.**
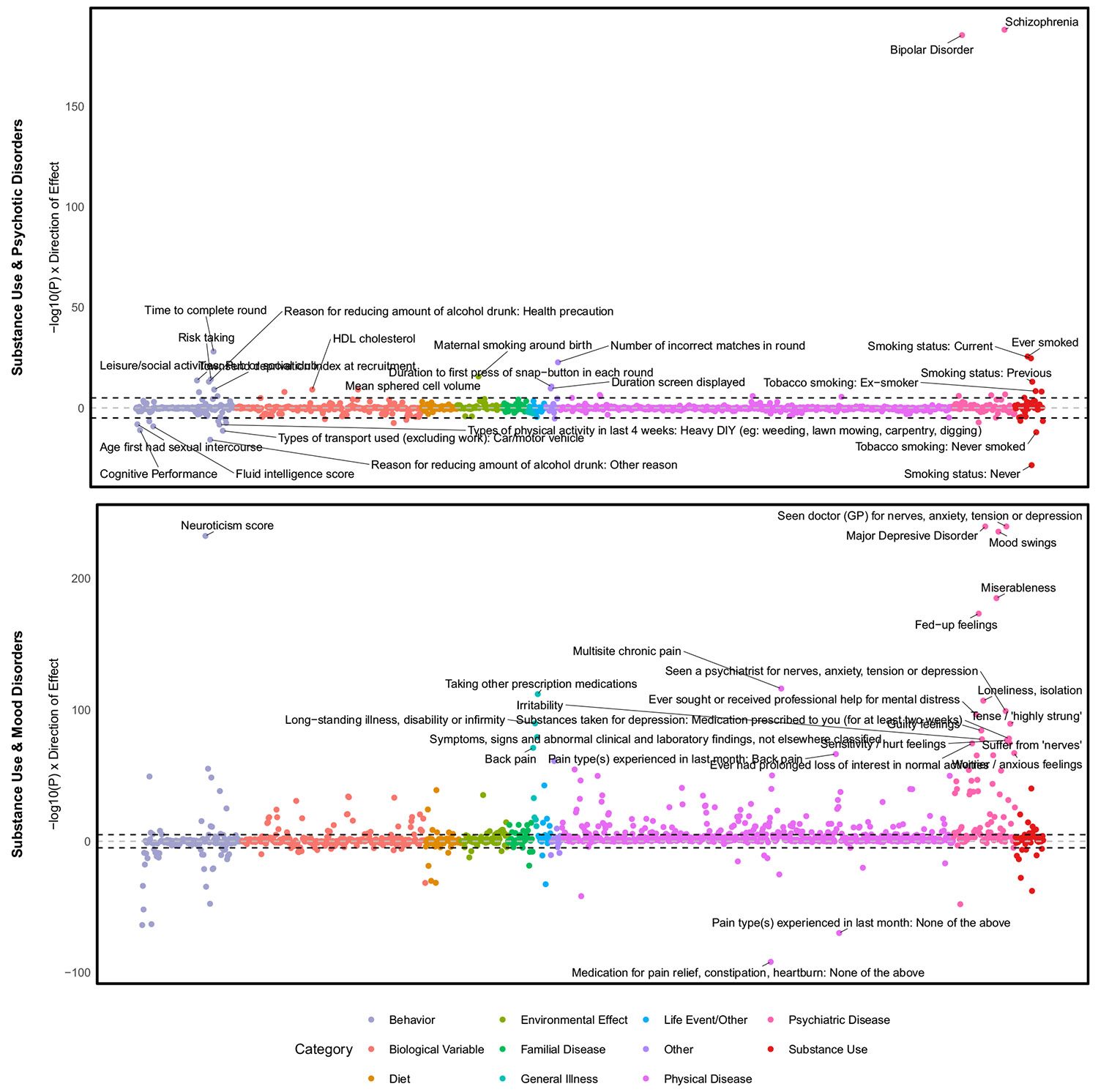
Genetic correlations for second-order common factors using Complex Traits Genetics Virtual Lab in European ancestry individuals. The y-axis represents the log-transformed false discovery rate–corrected *p* values across the 1437 traits included in the analysis, with the dashed line representing the significance threshold. Results of a similar analysis in African ancestry individuals are shown in Figure S35. DIY, do-it-yourself; GP, general practitioner; HDL, high-density lipoprotein.

The SUD and psychotic disorder factor correlated most strongly with SCZ and BD and less strongly, but still significantly, with smoking-related traits, cognitive measures, and risk taking (Figure 4). A PheWAS identified associations with tobacco and alcohol-related disorders (Figure S30 and Table S30). The SUD and mood factor correlated most strongly with mood, ANX, illness, and medication use for pain or gastrointestinal problems (Figure 4). Although a PheWAS showed the strongest associations with substance use and psychiatric disorders, it also showed associations with physical health (e.g., pain, type 2 diabetes, heart disease, and sleep disorders) (Figure S31 and Table S31).

#### African Ancestry.

The first-order SUD and psychiatric disorder factors were highly genetically correlated (*r*_g_ = 0.74, SE = 0.13, *p* < .001), and a second-order CFA accounting for this shared genetic risk fit well (χ^2^_19_ = 21.49, *p* = .31, AIC = 55.49, CFI = 0.99, SRMR = 0.10) (Figure S32 and Table S32). No significant SNPs were identified by the second-order GWAS (Figure S33).

There was enriched gene expression in brain regions associated with reward, emotion, memory processing, and executive functions (Figure S34). The second-order SUD and psychiatric factors correlated with all traits examined except PTSD, and the strongest correlations were with smoking trajectory, OUD, depression, and maximum alcohol consumption (Figure S35). A PheWAS of the second-order factor yielded no associations, but similar top hits were seen as in the first-order factors (Figure S36 and Table S33).

### GWAS-by-Subtraction in EUR

**Figure 5.**
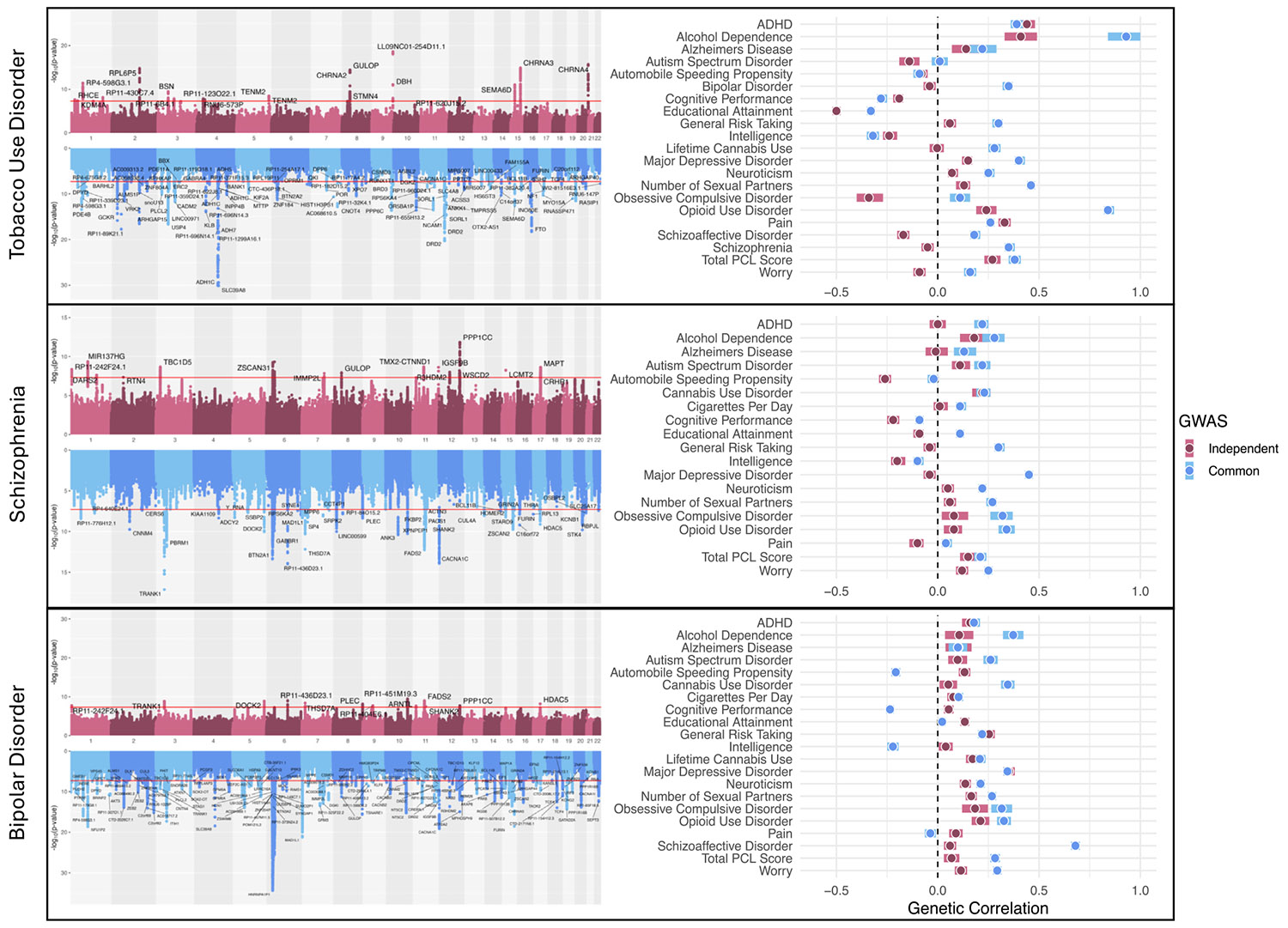
Hudson plots and genetic correlations of GWAS-by-subtraction models. The left panel presents GWAS-by-subtraction results modeled as Hudson plots, in which the upright plot is a Manhattan plot of independent GWAS results, and the inverted plot is a Manhattan plot of common GWAS results. Independent GWAS refers to genetic influences on a disorder that operate independently of the first-order common factor, while the common GWAS refers to influences on the disorder that operate through the common factor (Figure S2). Note that lead single nucleotide polymorphisms are annotated with their corresponding gene mappings. The right panel presents genetic correlations between specific traits and either the independent GWAS or the common GWAS. Note that due to limited statistical power in African ancestry individuals, GWAS-by-subtraction was performed only in European ancestry individuals. ADHD, attention-deficit/hyperactivity disorder; GWAS, genome-wide association study; PCL, PTSD Checklist.

Common and independent genetic effects were examined separately for TUD, SCZ, and BD (Figure 5) because these traits had a residual variance ≥0.35 in the first-order model. We identified 102 lead SNPs for TUD common, representing genetic effects on TUD that operate through the SUD factor, and 20 lead SNPs for TUD independent, representing genetic effects on TUD that operate independently from the SUD factor (Table S34). We identified 51 lead SNPs for SCZ common, 18 for SCZ independent (Table S35), 189 for BD common, and 13 for BD independent (Table S36).

Chromatin interaction mapping and gene-set analysis highlighted specificity within each trait. For TUD independent genetic effects, chromatin interaction mapping identified several nicotinic acetylcholine receptor genes (Figure S37), whereas for BD independent genetic effects, *MXI1* (54) and *ADD3* were identified, which were previously associated with BD (55) (Figure S38). SCZ independent mapped to the *ZSCAN* and *HIST1H* gene families (Figure S39), and the top gene set implicated upregulated genes in mouse models of 22q11.2 microdeletions, previously associated in humans with developing SCZ (56). For TUD, SCZ, and BD independent, PPIs were enriched (TUD = 3.31×, *p* = 3.45 × 10^−13^; SCZ = 5.48×, *p* < 1.00 × 10^−16^; BD = 5.75×, *p* = 2.19 × 10^−11^), indicating molecular mechanisms with enhanced specificity (Figures S40-S42).

Although TUD common was positively genetically correlated with SCZ, TUD independent was not (*r*_g_ = 0.35 vs. −0.05). SCZ common had a nominally weaker negative genetic correlation with cognitive performance (*r*_g_ = −0.09) and was positively correlated with educational attainment (*r*_g_ = 0.11), while SCZ independent had negative associations with both (*r*_g_ = −0.22 and −0.09). BD common and independent showed opposite associations with automobile speeding propensity and cognitive performance, both of which were negative for BD common (*r*_g_ = −0.21 and −0.24) and positive for BD independent (*r*_g_ = 0.13 and 0.05). BD independent was significantly associated with MDD (*r*_g_ = 0.35), whereas SCZ independent was not (*r*_g_ = −0.04). (Figure 5 and Tables S37-S39).

TUD common showed associations with multiple SUDs, whereas TUD independent had the highest associations with TUD and related medical conditions, such as chronic airway obstruction (Figure S43). SCZ common showed broad associations with mood disorders, but there were no associations for SCZ independent (Figure S44). Neither SCZ common nor SCZ independent was associated with SCZ, likely due to the small number of individuals with SCZ in the PMBB (57). Both BD common and independent showed associations with BD and other mood disorders, but only BD independent was associated with depression (Figure S45).

## DISCUSSION

Leveraging the largest available GWASs in EUR and AFR individuals, we combined multivariate approaches to examine the shared genetic architecture across SUDs and psychotic, mood, and ANX disorders. Consistent with other findings (17,58), we identified common genetic factors that underlie disorders with shared features. Although smaller samples and greater genetic diversity limited power to replicate the EUR factor structure in AFR individuals, there were commonalities across groups. For example, in both AFR and EUR models, SUDs loaded onto a single factor that was highly genetically correlated with a previously identified genetic addiction factor (17). Additionally, transancestry genetic correlations highlighted the consistency of genetic influences in AFR and EUR individuals.

In AFR individuals, we identified a lead SNP (rs1944683) associated with SUDs that has previously been implicated in alcohol, tobacco, cannabis, and opioid-related traits in EUR and cross-ancestry GWASs (51-53), highlighting the ability of gSEM to improve statistical power by leveraging shared genetic structure across traits. In EUR individuals, we detected 2 novel loci for mood and ANX disorders. Chromatin interaction mapping of the lead SNPs implicated genes involved in immune and stress responses (*BTLA* and *NECTIN3*, respectively) and hippocampal development (*FOXP1*) (46,49,59,60). *BTLA* may contribute to immune dysregulation in psychiatric pathogenesis (61-63), whereas *NECTIN3* and *FOXP1* are involved in synaptic plasticity and implicated in stress-related disorders (46,48,49,64). This evidence suggests that dysfunctions in synaptic plasticity and immune regulation may underlie a range of psychiatric conditions (65,66).

In EUR individuals, both the SUD and psychotic disorder factors showed associations with genes expressed in brain tissue during prenatal development, underscoring the importance of early neurodevelopmental processes in shaping susceptibility to SUDs and psychiatric disorders. Prenatal development may represent a sensitive window during which genetic and environmental factors interact to influence adult mental health outcomes (67).

### SUDs Share Genetic Liability With Psychotic and Mood Disorders

We also identified higher-order dimensions of liability to psychopathology, including genetic risk shared between SUDs and psychotic disorders and between SUDs and mood/ANX disorders. PheWASs showed broad manifestations of the dimensions of genetic liability. In EUR individuals, the shared liability for SUDs and mood/ANX disorders was associated with poor physical health, including obesity, type 2 diabetes, chronic pain, heart disease, and sleep disorders. Genetic correlations with the second-order factors underscored these findings across ancestry groups. In EUR individuals, correlations were seen with lower cognitive performance, elevated high-density lipoprotein cholesterol, chronic pain, long-standing illness, and miserableness, whereas in AFR individuals, correlations were seen with pain intensity and various substance use and psychiatric phenotypes. Thus, genetic risk for co-occurring SUDs and psychiatric disorders has far-reaching implications for health.

In addition to shared variance, most disorders exhibited unique genetic variance. At the second-order level, SUDs shared more genetic variance with mood/ANX disorders than with psychotic disorders, as indicated by the lower residual variance of the mood factor than the psychotic disorder factor in this model. This suggests different degrees of genetic convergence between SUDs and common psychiatric comorbidities. Such genetic convergence may partially explain comorbidities, including those between AUD and MDD (1) and CanUD and SCZ (68), while the heterogeneity in genetic overlap suggests the presence of both shared and distinct mechanisms underlying these conditions.

### Specificity of Genetic Effects for TUD, SCZ, and BD

GWAS-by-subtraction models provided insights into the structure of psychotic disorders. SCZ and BD shared a common genetic core, consistent with shared psychotic features and empirical nosology (69). However, genetic risk that was specific to each disorder showed different associations with complex traits. For example, SCZ independent was more negatively genetically correlated with cognition and educational attainment than BD independent, which was more strongly associated with risk taking and affective disorders. Our results are consistent with the expanded psychosis continuum hypothesis, which proposes that although SCZ and BD share a psychotic core, cognitive and affective domains differentiate them (70).

PheWAS results further showcased the enhanced specificity of findings when combining disorder-level and transdiagnostic approaches. TUD independent exhibited associations not observed for TUD common, including ischemic heart disease, atherosclerosis, obesity, and skin conditions. This may reflect unhealthy lifestyle factors, physiological effects of tobacco use, or shared biological pathways that underlie these conditions. Recent research comparing transdiagnostic and disorder-specific genetic effects across psychiatric disorders similarly observed differences in genetic associations (71), as has research parsing alcohol-specific risk from externalizing liability (72). Thus, hierarchical genetic approaches facilitate a more nuanced understanding of comorbidity and heterogeneity across cooccurring conditions.

These findings pave the way for more refined PGSs than are currently available. Current depression PGSs show little specificity, accounting for a similar amount of variance in mood, ANX, attention-deficit/hyperactivity disorder, and SUDs (73). Similarly, 16 PGSs for psychiatric phenotypes were associated with general psychopathology rather than the specific domain for which they purported to measure risk (74). Although PGSs have clinical utility in predicting some nonpsychiatric disorders (75,76), performance for psychiatric phenotypes remains limited (77). Efforts to develop more precise PGSs may enhance clinical utility.

## Conclusions

Integrating transdiagnostic and disorder-level genetic models clarifies the biological underpinnings of psychiatric comorbidity and identifies distinct pathways that contribute to heterogeneity within classes of psychiatric disorders. Previous gSEM studies have been limited to EUR individuals (14,16,78), and application has been hampered in non-EUR groups due to smaller samples, lower LD, higher genetic diversity, and increased admixture (79,80). We provide details (Supplemental Methods) on the efforts that we made to ensure inclusion of AFR individuals, which we hope will aid researchers in studying other ancestry groups using gSEM methods as inclusivity in GWASs increases. Advancing genetic discovery across diverse populations will be key in shaping our understanding of psychiatric etiology.

## Supplementary Material

1

2

Supplementary material cited in this article is available online at doi.org/10.1016/j.biopsych.2025.04.021.

## Figures and Tables

**Table 1.** GWASs From Which Summary Statistics Were Obtained

Reference	Year	Phenotype	Type	Cases	Controls	Total
European Ancestry						
Als *et al*. (12)	2023	MDD	Case-control	387,429	976,554	1,363,983
Otowa *et al*. (22)^a^	2016	ANX	Case-control	7016	14,745	21,761
Levey *et al*. (6)^a^	2020	ANX	Continuous	–	–	175,163
Purves *et al*. (23)^a^	2020	ANX	Case-control	25,453	58,113	83,566
Trubetskoy *et al*. (7)	2022	SCZ	Case-control	53,386	77,258	130,644
Mullins *et al*. (8)	2021	BD	Case-control	41,917	371,549	413,466
Zhou *et al*. (11)	2023	AUD	Case-control	113,325	639,923	753,248
Levey *et al*. (10)	2023	CanUD	Case-control	42,281	843,744	886,025
Toikumo *et al*. (13)	2024	TUD	Case-control	163,734	331,271	495,005
Kember *et al*. (9)	2022	OUD	Case-control	31,473	394,471	425,944
African Ancestry						
Levey *et al*. (24)	2021	MDD	Case-control	25,843	33,757	59,600
Levey *et al*. (6)	2020	ANX	Continuous	–	–	24,448
Bigdeli *et al*. (21)	2021	SCZ	Case-control	7509	8612	16,121
Bigdeli *et al*. (21)	2021	BD	Case-control	3027	8097	11,124
Kember *et al*. (20)	2023	AUD	Case-control	25,012	52,313	77,325
Levey *et al*. (10)	2023	CanUD	Case-control	19,065	104,143	123,208
Toikumo *et al*. (13)	2024	TUD	Case-control	45,465	68,955	114,420
Kember *et al*. (9)	2022	OUD	Case-control	8968	79,530	88,498

See Tables S1 to S4 for a full description of cohorts.

ANX, anxiety; AUD, alcohol use disorder; BD, bipolar disorder; CanUD, cannabis use disorder; GWAS, genome-wide association study; MDD, major depressive disorder; OUD, opioid use disorder; SCZ, schizophrenia; TUD, tobacco use disorder.

a Summary statistics were jointly analyzed using the multitrait analysis of GWAS to enhance the statistical power of the ANX phenotype prior to being used in genomic structural equation modeling (see Supplemental Methods).

**Table:** KEY RESOURCES TABLE

Resource Type	Specific Reagent or Resource	Source or Reference	Identifiers	Additional Information
Add additional rows as needed for each resource type	Include species and sex when applicable.	Include name of manufacturer, company, repository, individual, or research lab. Include PMID or DOI for references; use “this paper” if new.	Include catalog numbers, stock numbers, database IDs or accession numbers, and/or RRIDs. RRIDs are highly encouraged; search for RRIDs at https://scicrunch.org/resources.	Include any additional information or notes if necessary.
Deposited Data; Public Database	GWAS of major depressive disorder (EUR, AFR ancestry)	https://doi.org/10.1101/2022.08.24.22279149	GWAS Catalog: 37464041	Summary statistics available at: https://ipsych.dk/en/research/downloads/
Deposited Data; Public Database	GWAS of anxiety disorders (EUR ancestry)	https://doi.org/10.1038/mp.2015.197	GWAS Catalog: 26754954	
Deposited Data; Public Database	GWAS of GAD-2 scores (EUR, AFR ancestry)	https://doi.org/10.1176/appi.ajp.2019.19030256	GWAS Catalog: 31906708	
Deposited Data; Public Database	GWAS of lifetime anxiety disorder (EUR ancestry)	https://doi.org/10.1038/s41380-019-0559-1	GWAS Catalog: 31748690	
Deposited Data; Public Database	GWAS of schizophrenia (EUR ancestry)	https://doi.org/10.1038/s41586-022-04434-5	FigShare: 10.6084/m9.figshare.19426775	
Deposited Data; Public Database	GWAS of schizophrenia (AFR ancestry)	https://doi.org/10.1093/schbul/sbaa133	GWAS Catalog: 33169155	
Deposited Data; Public Database	GWAS of bipolar disorder (EUR ancestry)	https://doi.org/10.1038/s41588-021-00857-4	FigShare: 10.6084/m9.figshare.14102594	
Deposited Data; Public Database	GWAS of bipolar disorder (AFR ancestry)	https://doi.org/10.1093/schbul/sbaa133	GWAS Catalog: 33169155	
Deposited Data; Public Database	GWAS of alcohol use disorder (EUR ancestry)	https://doi.org/10.1038/s41591-023-02653-5	dbGaP: phs001672	Summary statistics also available at: https://medicine.yale.edu/lab/gelernter/stats/
Deposited Data; Public Database	GWAS of alcohol use disorder (AFR ancestry)	https://doi.org/10.1176/appi.ajp.21090892	GWAS Catalog: 37282553	
Deposited Data; Public Database	GWAS of cannabis use disorder (EUR, AFR ancestry)	https://doi.org/10.1038/s41588-023-01563-z	dbGap: phs001672.v7.p1	
Deposited Data; Public Database	GWAS of tobacco use disorder (EUR, AFR ancestry)	https://doi.org/10.1038/s41562-024-01851-6	N/A	Summary statistics available at: https://psychemerge.com/
Deposited Data; Public Database	GWAS of opioid use disorder (EUR, AFR ancestry)	https://doi.org/10.1038/s41593-022-01160-z	dbGaP: phs001672	
Software; Algorithm	GenomicSEM v0.0.5c	https://doi.org/10.1038/s41562-019-0566-x	N/A	GitHub: https://github.com/GenomicSEM/GenomicSEM?tab=readme-ov-file
Software; Algorithm	R v4.4.2	The R Foundation	RRID: SCR_001905	
Software; Algorithm	LDSC v1.0.1	https://doi.org/10.1038/ng.3211	RRID:SCR_022801	
Software; Algorithm	FUMA v1.6.0	https://doi.org/10.1038/s41467-017-01261-5	RRID:SCR_017521	
Software; Algorithm	MAGMA v1.08	DOI: 10.1186/1471-2105-4-30	RRID:SCR_005757	
Software; Algorithm	STRING v12.0	DOI: 10.1093/nar/gkac1000	RRID:SCR_005223	
Software; Algorithm	Complex-Traits Genetics Virtual Lab	https://doi.org/10.1101/518027	N/A	Tool accessible at: https://vl.genoma.io/
Software; Algorithm	PRS-CS	https://doi.org/10.1038/s41467-019-09718-5	N/A	GitHub: https://github.com/getian107/PRScs
Software; Algorithm	PheWAS R package	DOI: 10.1093/bioinformatics/btu197	RRID:SCR_003512	
Software; Algorithm	Popcorn v1.0	DOI: 10.1016/j.ajhg.2016.05.001	N/A	GitHub: https://github.com/brielin/Popcorn
