# Supplementary material for: Transdiagnostic and Disorder-Level Genome-Wide Association Studies Enhance Precision of Substance Use and Psychiatric Genetic Risk Profiles in African and European Ancestries
Source: Biol Psychiatry. Author manuscript; Available in PMC 2026 Apr 17. (PMC13088903; doi:10.1016/j.biopsych.2025.04.021)
Supplement: 1 [file NIHMS2161994-supplement-1.pdf]

SUPPLEMENTARY INFORMATION

Transdiagnostic and Disorder-Level Genome-Wide Association Studies Enhance Precision of Substance Use and Psychiatric Genetic Risk Profiles in African and European Ancestries

Khan *et al.*

Supplementary Methods **4**

MTAG 4

Procedures for Summary Statistics in GenomicSEM 4

African Ancestry Reference Panels 5

GenomicSEM 7

LD Clumping & Identification of Novel Lead SNPs 8

SNP-Level PheWAS 8

Biological Characterization 8

Genetic Correlations 9

Polygenic Score-Based PheWAS 9

Supplementary Results **10**

MTAG 10

Genetic Correlations among Input GWAS 10

Exploratory and Confirmatory Factor Analysis 10

Biological Characterization of First-Order Common Factor GWAS Findings 11

Supplementary Figures **12**

Supplementary Figure 1. Common and independent pathway models to identify factor specific Q_{SNPs} 12

Supplementary Figure 2. GWAS-by-subtraction pathway models 13

Supplementary Figure 3. Manhattan plot comparison of Otowa, et al., 2016 anxiety disorders GWAS prior to and after applying MTAG 14

Supplementary Figure 4. Genetic correlations of input GWAS in European ancestry individuals 15

Supplementary Figure 5. Genetic correlations of input GWAS in African ancestry individuals 16

Supplementary Figure 6. Regional annotation plot for rs75174029, a novel SNP identified by the European ancestry mood/anxiety disorders GWAS. 17

Supplementary Figure 7. PheWAS plots of novel SNPs for the mood disorders common factor 18

Supplementary Figure 8. Regional annotation plot for rs7652704, a novel SNP identified by the European ancestry mood/anxiety disorders GWAS. 19

Supplementary Figure 9. Results of MAGMA tissue expression analysis of EUR substance use disorders factor 20

Supplementary Figure 10. Results of MAGMA tissue expression analysis of EUR psychotic disorders factor . 21

Supplementary Figure 11. Results of MAGMA tissue expression analysis of EUR mood disorders factor 22

Supplementary Figure 12. Genetic correlation results for the EUR substance use disorders factor 23

Supplementary Figure 13. Genetic correlation results for the EUR psychotic disorders factor 24

Supplementary Figure 14. Genetic correlation results for the EUR mood disorders factor 25

Supplementary Figure 15. PheWAS results for the EUR substance use disorders factor in Penn Medicine BioBank 26

Supplementary Figure 16. PheWAS results for the EUR psychotic disorders factor in Penn Medicine BioBank 27

Supplementary Figure 17. PheWAS results for the EUR mood disorders factor in Penn Medicine BioBank .. 28

Supplementary Figure 18. Manhattan plot for substance use disorders factor in AFR ancestry individuals 29

Supplementary Figure 19. Manhattan plot for psychiatric disorders factor in AFR ancestry individuals 30

Supplementary Figure 20. Results of MAGMA tissue expression analysis of AFR ancestry substance use disorders factor 31

Supplementary Figure 21. Results of MAGMA tissue expression analysis of AFR ancestry psychiatric disorders factor 32

Supplementary Figure 22. Genetic correlations between AFR ancestry common factors and psychiatric and substance use phenotypes 33

Supplementary Figure 23. PheWAS results for AFR ancestry substance use disorders factor in Penn Medicine BioBank 34

Supplementary Figure 24. PheWAS results for AFR ancestry psychiatric disorders factor in Penn Medicine BioBank 35

Supplementary Figure 25. EUR ancestry second order common factor model 36

Supplementary Figure 26. Manhattan plot for second-order common factor representing overlap between substance use and psychotic disorders in EUR ancestry individuals 37

Supplementary Figure 27. Manhattan plot for second-order common factor representing overlap between substance use and mood/anxiety disorders in EUR ancestry individuals 38

Supplementary Figure 28. Results of MAGMA tissue expression analysis of EUR ancestry second-order substance use and psychotic disorders factor 39

Supplementary Figure 29. Results of MAGMA tissue expression analysis of EUR ancestry second-order substance use and mood disorders factor 40

Supplementary Figure 30. PheWAS results for EUR ancestry second-order common factor representing overlap in substance use and psychotic disorders in Penn Medicine BioBank 41

Supplementary Figure 31. PheWAS results for EUR ancestry second-order common factor representing overlap in substance use and mood/anxiety disorders in Penn Medicine BioBank 42
Supplementary Figure 32. AFR ancestry second order common factor model 43
Supplementary Figure 33. Manhattan plot for second-order common factor representing overlap between substance use and psychiatric disorders in AFR ancestry individuals 44
Supplementary Figure 34. Results of MAGMA tissue expression analysis of AFR ancestry second-order substance use and psychiatric disorders factor 45
Supplementary Figure 35. Genetic correlations between the AFR ancestry second-order common factor and psychiatric and substance use traits 46
Supplementary Figure 36. PheWAS results for AFR ancestry second-order common factor representing overlap in substance use and psychiatric disorders in Penn Medicine BioBank 47
Supplementary Figure 37. Significant SNPs identified in TUD Independent GWAS 52
Supplementary Figure 38. Significant SNPs identified in BD Independent GWAS 57
Supplementary Figure 39. Significant SNPs identified in SCZ Independent GWAS 63
Supplementary Figure 40. Protein-protein interaction network plot for TUD Independent. 64
Supplementary Figure 41. Protein-protein interaction network plot for SCZ Independent. 65
Supplementary Figure 42. Protein-protein interaction network plot for BD Independent. 66
Supplementary Figure 43. Hudson plot of PheWAS results for tobacco use disorders GWAS-by-subtraction in Penn Medicine BioBank 67
Supplementary Figure 44. Hudson plot of PheWAS results for schizophrenia GWAS-by-subtraction in Penn Medicine BioBank 68
Supplementary Figure 45. Hudson plot of PheWAS results for bipolar disorder GWAS-by-subtraction in Penn Medicine BioBank 69

Supplementary Methods

MTAG

Compared to other psychiatric traits included our analysis of European ancestry individuals, available GWAS of anxiety disorders were comparatively underpowered (Supplementary Table 1). Consequently, we used Multi-Trait Analysis of GWAS (MTAG)(1) to leverage the genetic effects from a study of Lifetime Anxiety Disorder(2) and a study of GAD-2 questionnaire scores(3) to enhance the statistical power of a GWAS for a broad spectrum of anxiety disorders(4) in European ancestry individuals. We chose to enhance the power of the summary statistics from Otowa, et al., 2016, because they included the most diverse array of anxiety disorders among the three anxiety GWAS. This choice was supported by the strong genetic correlations between the generalized anxiety GWAS(4) and both GWAS of lifetime anxiety disorder ($r_g = 0.7429$) and GAD-2 scores ($r_g = 0.7309$) (Supplementary Table 5).

Effective sample sizes were calculated as the sum of $4/(1/n\_\text{case} + 1/n\_\text{control})$ for each cohort in each of the two case-control GWAS. For the GAD-2 score GWAS, the total sample size was used as the input for MTAG because GAD-2 is a continuous trait. As quality control measures included in the MTAG software, SNPs with MAF<0.01 were excluded from analysis, along with duplicate SNPs and those with missing values. Following MTAG analysis, the effective sample size for follow-up analyses was calculated using the formula described by Turley, et al., 2018.(1)

Procedures for Summary Statistics in GenomicSEM

All summary statistics and analyses were conducted on the NCBI hg19/GRCh37 genome assembly. For traits with continuous outcomes (i.e., GAD-2 score), the total sample size was used for LDSC and GenomicSEM computations. For traits with a binary outcome (i.e., case-control), the effective sample size column contained within the GWAS summary statistics was used. When no effective sample size column was present, effective sample size was calculated for each set of summary statistics using the formula described by Grotzinger, et al, 2023:(5)

$$N_{eff} = \sum_{k=1}^{N} 4 * v_k * (1 - v_k) * n_k$$

Where v and n are the sample prevalence and sample total, respectively, for the kth cohort of a GWAS meta-analysis of N cohorts. Summary statistics were then prepared for GWAS using the following options in GenomicSEM: The “se.logit” flag was set to “TRUE” when the standard error column reflected the standard error of a logistic beta, the “OLS” flag was set to “TRUE” when the phenotype reflected a continuous outcome, and the “linprob” flag was set to “TRUE” when the phenotype was of a binary outcome but with only Z-statistics present as a measure of effect in the GWAS summary statistics. SNPs were then filtered based on MAF>0.01 and 0.6. Following preparation of summary statistics, 2,083,079 SNPs remained for analysis in the European-ancestry subset, and 6,350,709 SNPs remained for analysis in the African-ancestry subset.

gSEM analysis requires computation of the genetic covariance matrix and corresponding sampling covariance matrix for included traits, which we computed using linkage disequilibrium score regression (LDSC) in GenomicSEM 0.0.5c.(6) As part of this process, we also computed

and reported genetic correlations between included traits. When SNP-level sample sizes were not available within summary statistics, we calculated the effective sample size using the sum of effective sample sizes across the GWAS cohorts.(5) For EUR analyses, SNPs were restricted to those contained within the EUR HapMap3 reference panel(7) with a minor allele frequency (MAF) > 0.01. We performed LDSC using EUR 1000 Genomes Phase 3 linkage disequilibrium (LD) scores.(8) Given the statistical challenges associated with including non-EUR individuals in gSEM analyses due in part to differences in LD structure and admixture, we compared three sets of LD scores before performing LDSC in AFR individuals as described below.

African Ancestry Reference Panels

To determine the optimal linkage disequilibrium (LD) score reference panel for use in the African ancestry gSEM models, we compared three sets of references: (1) 1000 Genomes Phase 3, (2) PanUKB, and (3) Million Veteran Program (MVP). We used publicly available 1000 Genomes(8) and PanUKB(9) LD scores. MVP LD scores were generated from 1000 randomly selected African ancestry MVP participants using covariate-adjusted LD score regression (cov-LDSC),(10) which is a method that has shown improved performance among admixed populations, such as African Americans. As recommended to further account for population stratification, the first ten ancestry-specific principal components (PCs) were computed within the sample and included as covariates when generating LD scores.

To ensure the accuracy of and prevent bias in estimates derived from the LD scores, we restricted LD score regression (LDSC) analyses to well-imputed, biallelic autosomal SNPs that are outside of the MHC region. The set of SNPs meeting these criteria varied for each LD reference panel. For the 1000 Genomes Phase 3 panel, we used the list of 1,217,312 HapMap3 SNPs provided in the reference files prepared by Finucane et al. (2015)(11) for LDSC. For the PanUKB reference, we retained all 1,190,983 SNPs, as LD scores were computed only for SNPs that met the aforementioned criteria and passed additional quality control, including having imputation quality (R^2) > 0.90 and minor allele frequency > 0.01 (see https://pan-dev.ukbb.broadinstitute.org/docs/ld/index.html). For the MVP reference, we restricted our analyses to SNPs that met the same criteria as those used by the Broad Institute to prepare the PanUKB reference files. Thus, a total of 2,388 SNPs were removed due to low MAF, and 8,707 were removed due to low imputation quality, leaving 1,516,281 SNPs in MVP.

In comparing the performance of the three sets of reference panels, we evaluated: (1) the number of SNPs retained following filtering and munging the input summary statistics, (2) liability scale SNP-based heritability, (3) confounding evidenced by inflated values on the LDSC intercept, and (4) the length and distribution of resulting LD blocks. Results are presented below:

1000G reference, 1000G SNPlist				
**trait**	**# snps**	**heritability**	**SE**	**intercept**
AUD	423441	0.0806	0.0133	1.0304
TUD	691706	0.0445	0.008	1.0257
OUD	250024	0.0668	0.0229	1.0236
CanUD	604363	0.0616	0.0116	1.0306
GAD2	869992	0.0282	0.0365	1.0076
MDD	869312	0.0415	0.0188	1.0184
SCZ	891719	0.1204	0.0294	1.0587
BIP	891833	0.1417	0.0642	1.0344

MVP reference, MVP SNPlist				
**trait**	**# snps**	**heritability**	**SE**	**intercept**
AUD	1508956	0.0427	0.0062	1.0615
TUD	1515026	0.0434	0.0065	1.0631
OUD	1512615	0.0218	0.0092	1.0347
CanUD	1507988	0.021	0.0056	1.0464
GAD2	1476669	0.0391	0.0217	1.0056
MDD	1480787	1.00E-03	0.0082	1.0297
SCZ	1512163	0.0496	0.0155	1.0649
BIP	1511435	0.0554	0.0328	1.0324

UKBB reference, Pan-UKBB SNPlist				
**trait**	**# snps**	**heritability**	**SE**	**intercept**
AUD	613531	0.0885	0.0153	1.0352
TUD	979504	0.064	0.0083	1.0235
OUD	429964	0.0662	0.0206	1.034
CanUD	897996	0.068	0.0116	1.0289
GAD2	1152886	0.0619	0.0394	1.0026
MDD	1144306	0.0397	0.017	1.0193
SCZ	1184566	0.1661	0.0278	1.05
BIP	1183486	0.2238	0.0631	1.0254

Using the 1000 Genomes LD reference panel and SNP list resulted in the fewest number of SNPs remaining after performing LDSC on the input summary statistics, including as few as 250,024 SNPs for OUD. As LDSC accuracy decreases as the number of SNPs decreases,(12) we chose not to progress with the 1000 Genomes reference panels due to the potential for unreliable genetic correlations upon which gSEM models are based. On the other hand, the reference panels generated in MVP resulted in the largest number of remaining SNPs but tended to produce lower heritability estimates than the other reference panels, including a non-significant heritability estimate for MDD. MVP also consistently had the highest inflation in test statistics based on the

LDSC intercept. Finally, examining the distribution of the LD scores, MVP LD scores were consistently lower than those using PanUKB. As PanUKB reference panels resulted in an adequate number of SNPs available for analyses, produced significant heritability estimates, showed low inflation in test statistics, and had a broader distribution of LD scores compared to MVP (see below), we conducted African ancestry analyses using PanUKB references.

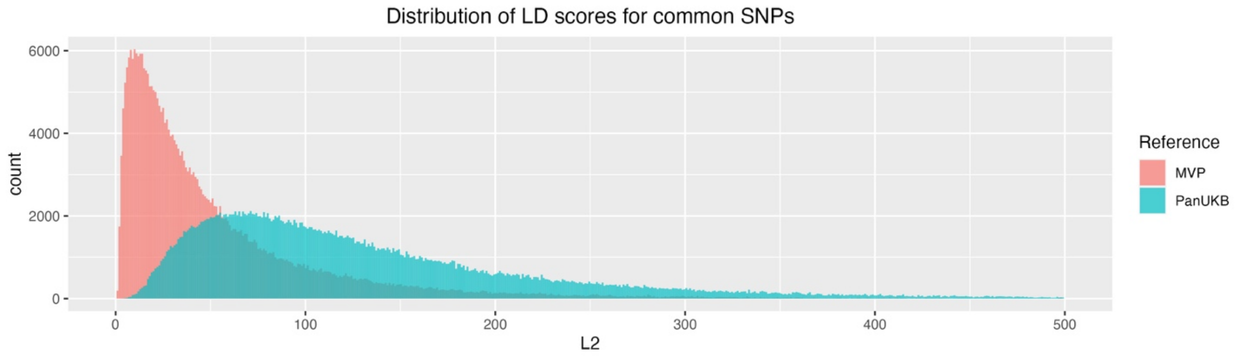

Comparison across ALL SNPs in both sets		
**Statistic**	**MVP L2**	**PanUKB L2**
Min	0.202	2.564
25th percentile	24.679	53.958
Median	48.104	86.065
Mean	77.451	110.042
75th percentile	92.463	136.970
Max	3672.834	3193.00

GenomicSEM

Prior to constructing common factor models, we performed exploratory (EFA) and confirmatory factor analysis (CFA) on independent data to evaluate the reliability of our results. We performed EFA on the odd chromosomes to evaluate the optimal number of factors and the loadings of each disorder in a hypothesis-free manner. Following EFA, we examined model fit (i.e., chi-square value, Akaike information criterion (AIC), comparative fix index (CFI), and standardized root mean squared residual (SRMR)) and eigenvalues to determine the optimal model.(13) Traits with a loading ≥0.35 were retained for CFA, which was performed on the even chromosomes to avoid overfitting the data. Because of limited statistical power in AFR models, factor analyses were performed on all chromosomes. Prior to performing GWAS, quality control filtering was applied to summary statistics by retaining only SNPs with a MAF > 0.01 and imputation score > 0.60.

After performing GWAS, we calculated Q_{SNP} to measure the heterogeneity of each SNP's effects. For example, a SNP that primarily influences SUDs through its effects on a single disorder, like TUD, should violate the null hypothesis. To identify heterogeneous SNPs, we examined associations between each SNP and common factor via a common pathway model. Then, separately for each factor, we fit an independent pathway model in which the SNP predicted each of the factor's indicators. By performing a chi-square difference test on the two models, we were able to test the null hypothesis that a SNP's effects operate entirely through the

common factor, and we removed SNPs with $p < 5 \times 10^{-8}$ prior to conducting all downstream analyses.

In accordance with our focus on characterizing shared genetic variance between SUDs and psychiatric disorders, we specified two second-order genetic factors using the summary statistics obtained from GWAS of the first-order factors: (1) SUD and psychotic disorders, and (2) SUD and mood (Supplementary Figures 25-27). Keeping with standard practice in bivariate SEM models, the loadings of each first-order factor onto the respective second-order factor were specified as the square root of the genetic correlation between the two first-order factors(14). GWAS then performed for each second-order factor with post-GWAS quality control procedures similar to those applied to first-order GWAS results.

LD Clumping & Identification of Novel Lead SNPs

Following common factor GWAS and GWAS-by-subtraction, LD clumping of summary statistics results was performed using PLINK 1.9(15) with ancestry-matched 1000 Genomes Phase 3 (for European) or PanUKB (for African) reference panels, a significance threshold of $5*10^{-8}$ for index SNPs, r^2 threshold of 0.10, and physical distance threshold of 3000kb. The lead SNP refers to the variant with the strongest statistical association in the region identified by GWAS. For common factor GWAS, lead SNPs were considered not to have been identified by any input GWAS if they were not located within +-1000kb of any lead SNP from any input study for the corresponding common factor. Lead SNPs from input studies were obtained from the supplementary materials for each input GWAS.

To determine if a lead SNP from common factor GWAS had previously been associated with any of the input traits by any previous study, a review of GWAS Catalog(16) was conducted. First, common factor GWAS lead SNP chromosome and base-pair information was lifted over from NCBI assembly hg19/GRCh37 to hg38/GRCh38 using the UCSC Genome Browser's LiftOver tool.(17) Then, for each lead SNP, a query of GWAS Catalog was conducted of all GWAS reporting significant SNPs in the range of +-1000kb of the lead SNP's position. The list of trait associations was subsequently reviewed for any terms corresponding to any input traits for the common factor GWAS. If there were no matches, then the SNP was considered novel in that it had not been previously associated with any previous GWAS of the input traits for a common factor at the time the search was conducted.

SNP-Level PheWAS

For any novel SNPs that were identified in GWAS, we performed a SNP-level PheWAS using GWAS Atlas.(18) Analyses examined 4,756 publicly available GWASs and used a Bonferroni corrected p-value of $1.05*10^{-5}$ to identify significant associations.

Biological Characterization

Biological Characterization. To investigate protein-protein interactions (PPI) of MAGMA-identified genes, we used STRING v12.0. Significant enrichment of PPI would suggest that proteins encoded by genes associated with a factor participate in common pathways.

Genetic Correlations

Genetic Correlations. For first- and second-order common factors in EUR individuals, we used the Complex-Traits Genetics Virtual Lab (CTG-VL)(19) to calculate genetic correlations with 1,437 traits. In AFR individuals, genetic correlations were calculated using LDSC for selected psychiatric and medical phenotypes due to limited inclusion of AFR individuals in available GWAS on CTG-VL. For GWAS-by-subtraction models, we calculated genetic correlations using LDSC with relevant psychiatric, social, and physical traits to compare the common and independent genetic effects. We applied a Benjamini-Hochberg false discovery rate (FDR) correction to account for multiple testing.

Polygenic Score-Based PheWAS

Prior to calculating polygenic scores (PGS) in the Penn Medicine Biobank (PMBB), GWAS were re-run excluding PMBB to ensure independence of the target sample. PGS were standardized, and PheWAS was conducted using logistic regression models, with sex, age, and the top 10 PCs included as covariates using the PheWAS R package(20). An FDR p-value was calculated to ascertain significance.

PMBB participants are recruited through the University of Pennsylvania Health System and provide access to their electronic health record (EHR) and blood or tissue samples.(21) Genotyping and imputation procedures within PMBB(21) resulted in 10,383 AFR and 29,355 EUR individuals. International Classification of Diseases (ICD)-9 and ICD-10 codes were gathered from EHRs and mapped to phecodes. Cases had at least two instances of a given ICD code ("phecodes").

Supplementary Results

MTAG

Prior to enhancing the statistical power of the summary statistics from Otowa, et al. 2016, 1 genome-wide significant lead SNP was present at the genome-wide significance threshold of $5*10^{-8}$. After applying MTAG, 5 lead SNPs were identified. Additionally, the sample size of the Otowa, et al. 2016 GWAS prior to applying MTAG was n = 17,310, whereas the effective sample size after applying MTAG was n = 115,651. A summary of lead SNPs is shown below, and a comparison of Manhattan plots prior to and after applying MTAG is shown in Supplementary Figure 3.

**Anxiety (pre-MTAG, Otowa, et al. 2016)**	
**Lead SNP**	**P-value**
rs1709393	1.65e-08

**Anxiety (post-MTAG, Otowa, et al. 2016, Purves, et al. 2019, Levey, et al., 2021)**	
**Lead SNP**	**P-value**
rs17794133	3.44e-11
rs7027175	4.22e-09
rs4937872	9.26e-09
rs6047130	1.3e-08
rs10434704	3.29e-08

Genetic Correlations among Input GWAS

European Ancestry. Genetic correlations among SUDs ranged from 0.60 (SE = 0.06, $p <$ 0.001; TUD and OUD) to 0.92 (SE = 0.05, $p < 0.001$; AUD and OUD). MDD and ANX were strongly genetically correlated ($r_g = 0.91$, SE = 0.04, $p < 0.001$), as were BD and SCZ ($r_g = 0.68$, SE = 0.03, $p < 0.001$). SUDs were significantly genetically correlated with the other psychiatric disorders as well.

African Ancestry. All four SUDs exhibited significant genetic correlations with one another. MDD and ANX were significantly correlated ($r_g = 0.89$, SE = 0.385, $p = 0.021$), as were BD and SCZ ($r_g = 0.43$, SE = 0.17, $p = 0.011$). Across disorder classes, AUD was correlated with all the psychiatric disorders except BD ($r_g = 0.19$, SE = 0.15, $p = 0.21$). TUD significantly correlated with BD ($r_g = 0.24$, SE = 0.12, $p = 0.037$) and SCZ ($r_g = 0.29$, SE = 0.10, $p = 0.005$). CanUD was genetically correlated with all the psychiatric disorders except ANX ($r_g = 0.29$, SE = 0.20, $p = 0.145$), and OUD was correlated with all.

Exploratory and Confirmatory Factor Analysis

In EUR individuals, we performed EFA to evaluate 1- through 4-factor models. Although select fit parameters were optimized in the 4-factor model, ($\chi^2(2) = 94{,}285$, CFI = 0.981, SRMR

= 0.017), there were several indications of overfitting in the model. First, there was a cross-loading of CanUD onto two factors, which decreases the interpretability of the result and is not carried forward into CFA by standard practice(22). More recent work suggests that when deciding how many factors to retain for CFA in the case of cross-loading on EFA, obtaining an "established set of common factors with clear interpretability over the relationships between variables" is the ultimate goal(23). Given that the 4-factor model would place CanUD on an additional separate factor from that of the rest of the SUDs, whereas the 3-factor model places CanUD on the same factor as the other SUDs which is consistent with prior research(24), we ultimately decided that the 3-factor model is the best theoretical fit of our data. This decision is supported by the fit parameter of RMSEA, which is the lowest in the 3-factor model (RMSEA = 0.228).

In AFR individuals, we tested two CFA models: a 2-factor model representing SUDs and psychiatric disorders and a 3-factor model replicating the EUR factor structure. The 3-factor CFA produced a negative residual variance and a correlation >1 between the psychotic and mood disorder factors. Thus, we proceeded with the 2-factor CFA model, which had an adequate fit ($\chi^2(19) = 21.49$, $p = 0.31$, AIC = 55.49, CFI = 0.99, SRMR = 0.10) and required no constraints (Supplementary Table 23).

Biological Characterization of First-Order Common Factor GWAS Findings

In characterizing the novel SNPs identified by GWAS of the mood disorders factor in EUR individuals, Hi-C data revealed that rs75174029 contacts several regions of *FOXP1*, including its promoter (Supplementary Figure 6). rs75174029 is an intronic variant in *FOXP1*, which is a key regulatory gene in neural development(25, 26). rs7652704 and 20 other SNPs in strong LD all had PICS probabilities of 0.027, suggesting difficulty in determining the most likely causal variant in the locus (Supplementary Table 19). rs7652704 is an eQTL of *NECTIN3* (also known as *PVLR3)* in cultured fibroblasts and displays chromatin interaction with *BTLA*, a gene involved in immune response,(27) and *NECTIN3*, which regulates cell organization and modulates stress responses.(28-30)

Supplementary Figures

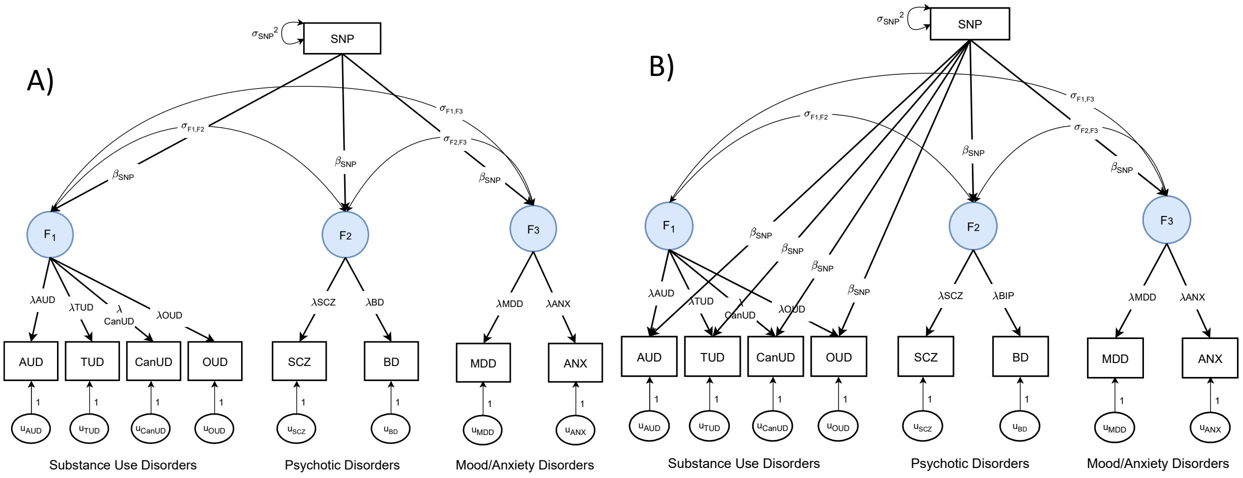

Supplementary Figure 1. Common and independent pathway models to identify factor specific Q_{SNPs}

Panel A depicts the common pathway model where a given SNP's effects operate through the factors. Panel B depicts the independent pathway model for Factor 1. In this model, each SNP predicts the indicators of Factor 1, as well as the other two factors. A χ^2 difference test was performed for the two models to determine if the SNP's effects could be explained by its association with the factor or, instead, by its association with specific indicators. Follow-up independent pathway models (as shown in Panel B) were run for the each of the other two first-order factors to identify their factor-specific Q_{SNPs}. An analogous approach was applied for the second-order factors and for African ancestry models. SNPs whose χ^2 p-value was $< 5*10^{-8}$ were removed from summary statistics prior to performing downstream analyses.

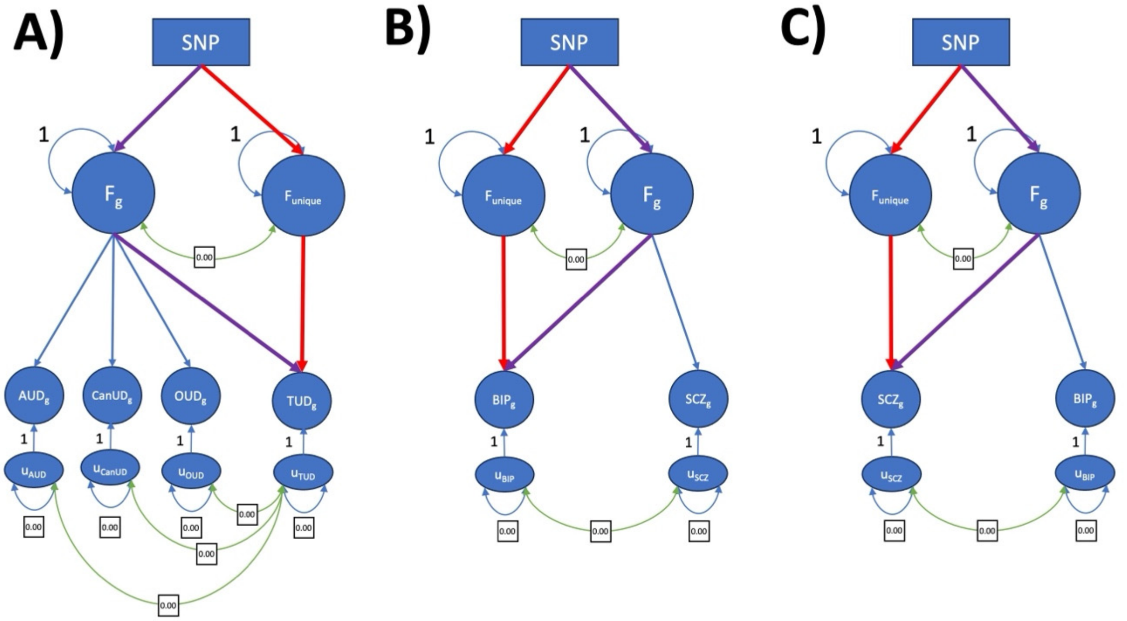

Supplementary Figure 2. GWAS-by-subtraction pathway models

Panel A depicts GWAS-by-subtraction of tobacco use disorder (TUD), whereas panels B and C depict GWAS-by-subtraction of bipolar disorder (BIP) and schizophrenia (SCZ), respectively. For each analysis, a general factor F_g was specified reflecting the respective first-order common factor, and a unique factor F_{unique} was specified with only the specific trait of interest loading onto it. The covariances between the two factors and between the traits were constrained to zero. Each SNP was then regressed onto F_g to estimate genetic effects that operated through the common pathway (purple arrow), and again onto F_{unique} to estimate genetic effects that operated through the independent pathway (red arrow).

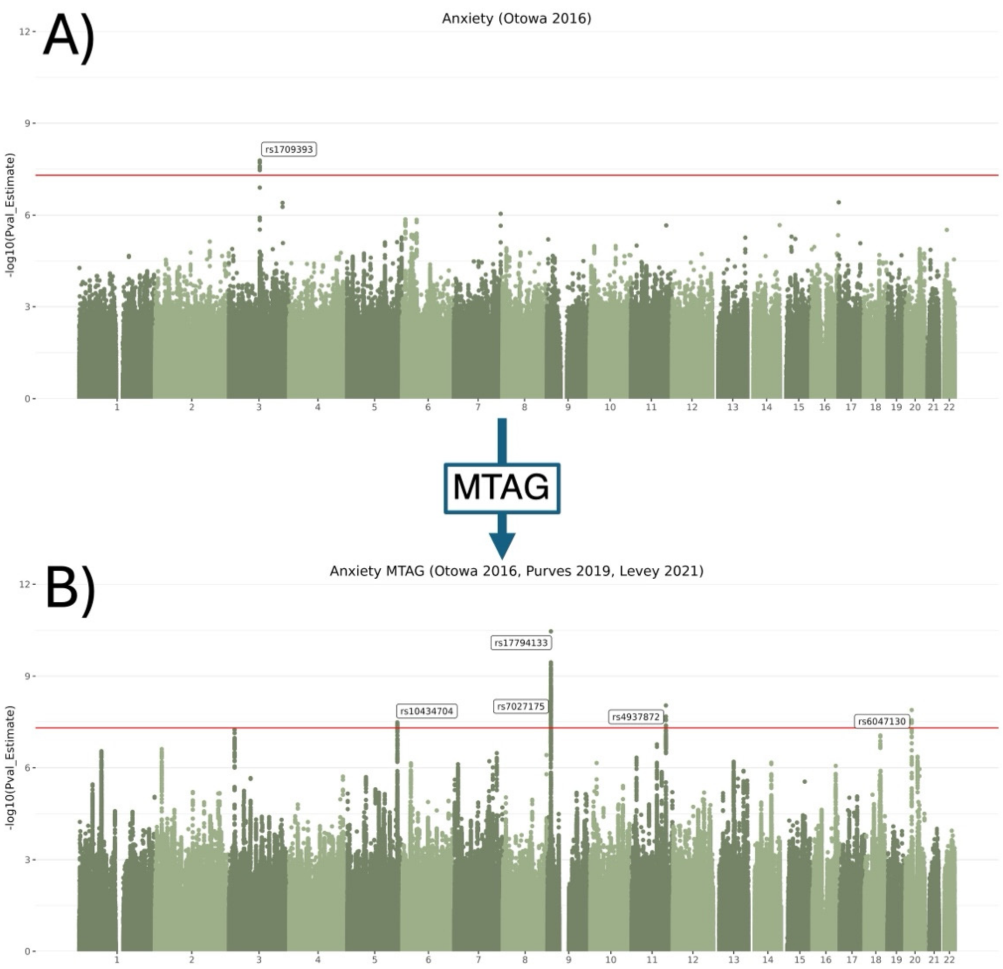

Supplementary Figure 3. Manhattan plot comparison of Otowa, et al., 2016 anxiety disorders GWAS prior to and after applying MTAG

Panel A depicts the results from the univariate GWAS of anxiety disorders by Otowa, et al. 2016, prior to the application of MTAG. After using summary statistics from GWAS of Lifetime Anxiety Disorder by Purves, et al., 2019 and GWAS of a 2-item General Anxiety Disorder questionnaire by Levey, et al., 2021 to enhance the statistical power of the anxiety disorders summary statistics, 5 lead SNPs were identified (Panel B).

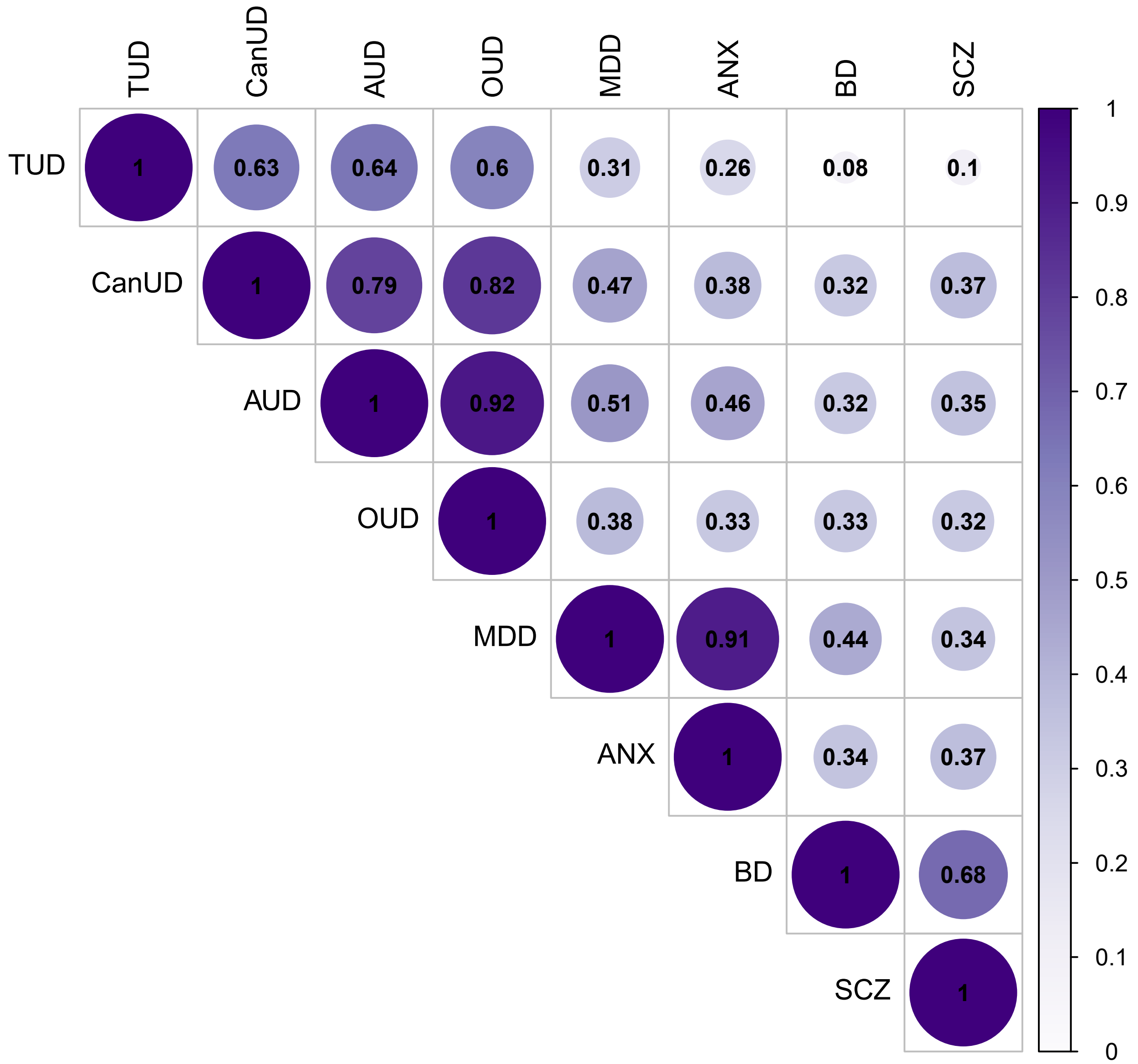

Supplementary Figure 4. Genetic correlations of input GWAS in European ancestry individuals

AUD = alcohol use disorder, CanUD = cannabis use disorder, TUD = tobacco use disorder, OUD = opioid use disorder, MDD = major depressive disorder, BD = bipolar disorder, ANX = anxiety disorders, SCZ = schizophrenia. Traits are ordered based on hierarchical clustering.

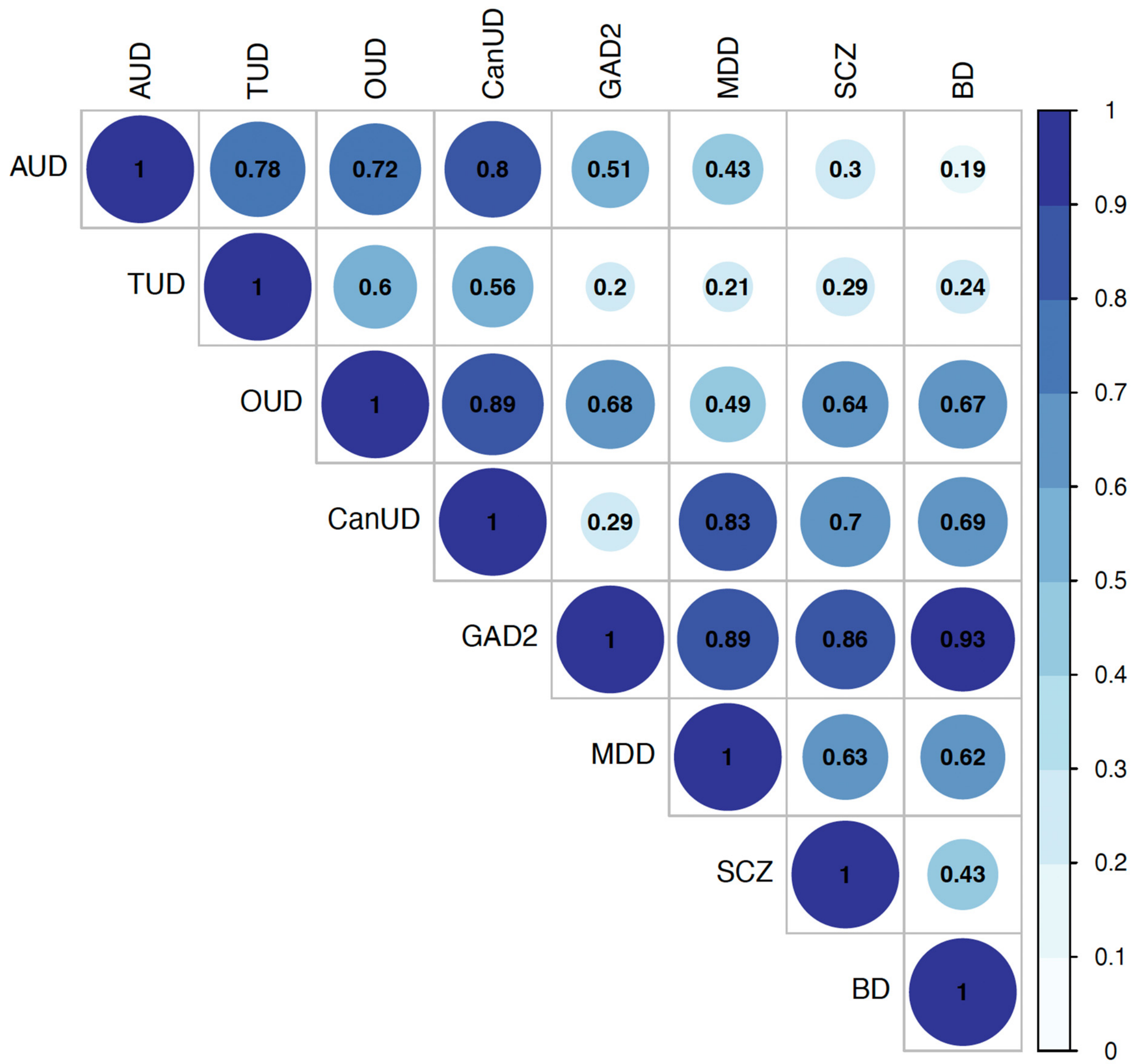

Supplementary Figure 5. Genetic correlations of input GWAS in African ancestry individuals
MDD = major depressive disorder, BD = bipolar disorder, GAD-2 = Generalized Anxiety Disorder-2 scores, SCZ = schizophrenia, AUD = alcohol use disorder, TUD = tobacco use disorder, CanUD = cannabis use disorder, OUD = opioid use disorder. Traits are ordered based on hierarchical clustering.

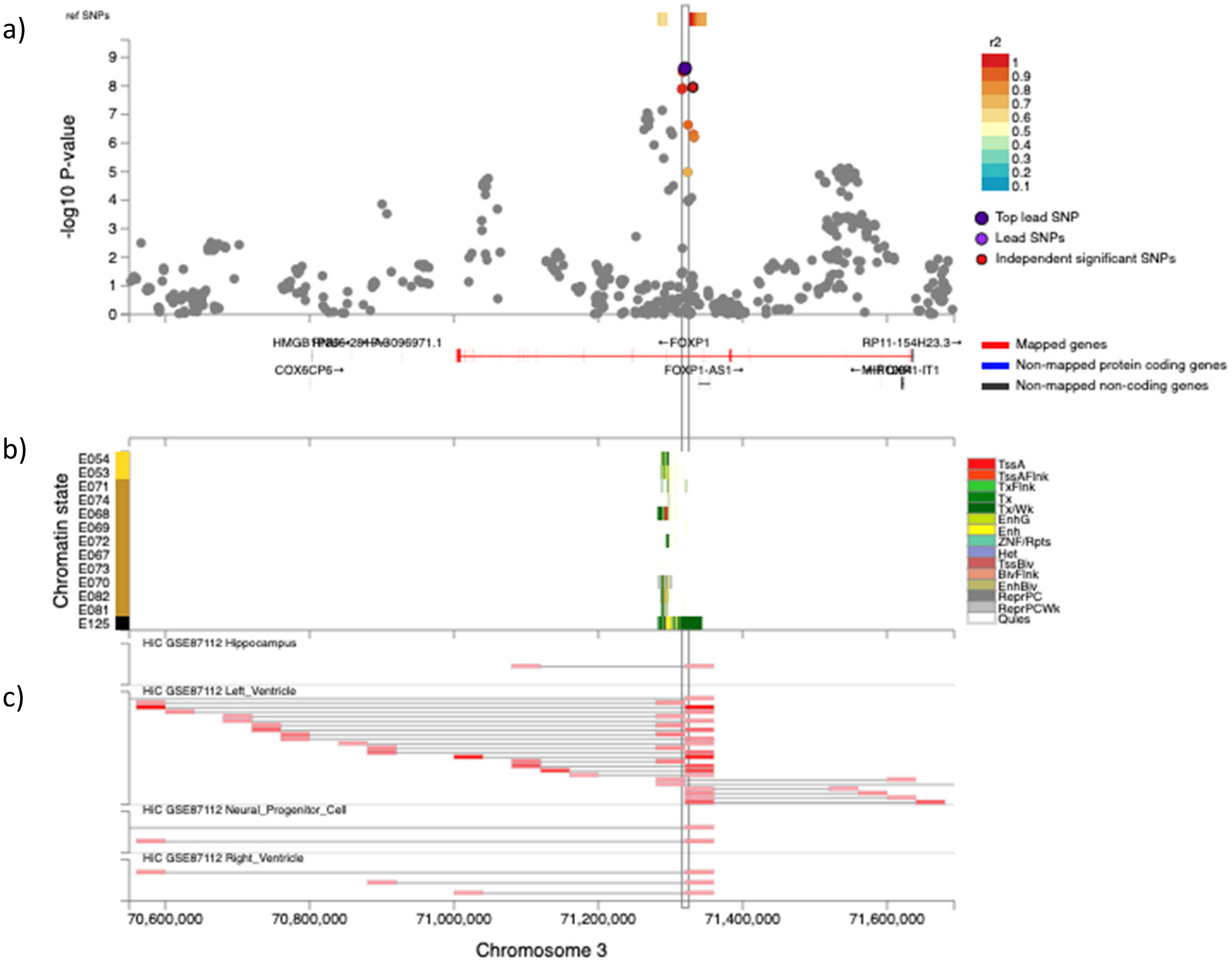

Supplementary Figure 6. Regional annotation plot for rs75174029, a novel SNP identified by the European ancestry mood/anxiety disorders GWAS.

(a) rs75174029 (in purple), its linked SNPs, and their position relative to genes. rs75174029's predicted genomic target *FOXP1* is shown in red. **(b)** Colocalization of rs75174029 with ROADMAP 15 core chromatin states (right-hand key) in 15 brain tissues (left hand key). E054 = ganglion eminence-derived neurospheres, E053 = cortex-derived neurospheres, E071 = hippocampus, E074 = substantia nigra, E068 = anterior caudate, E069 = cingulate gyrus, E072 = inferior temporal lobe, E067 = angular gyrus, E073 = dorsolateral prefrontal cortex, E070 = germinal matrix, E082 = female fetal brain, E081 = fetal male brain, E125 = NH-A astrocytes. TssA = Active Transcription Start Site, TsAFlnk = flanking active TSS, TxFlnk = transcribed at gene 5' and 3', Tx = strong transcription, TxWk = weak transcription, EnhG = genic enhancers, Enh = enhancers, ZNF/Rpts = ZNF genes and repeats, Het = heterochromatin, TssBiv = bivalent/poised TSS, BivFlnk = Flanking bivalent TSS/Enh, EnhBiv = bivalent enhancer, ReprPC = repressed PolyComb, PreprPCWk = weak repressed PolyComb, Quies = quiescent/low. **(c)** Colocalization with Hi-C signal in brain tissues. Each line represents an interaction, with the two red regions representing the loci which make contact.

rs75174029

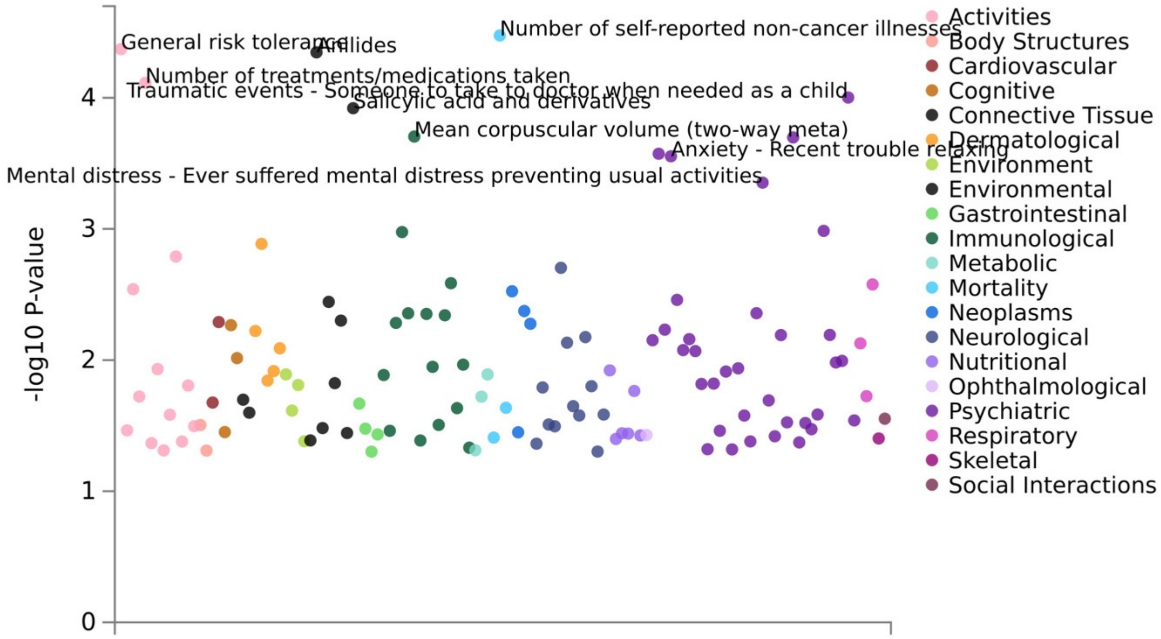

rs7652704

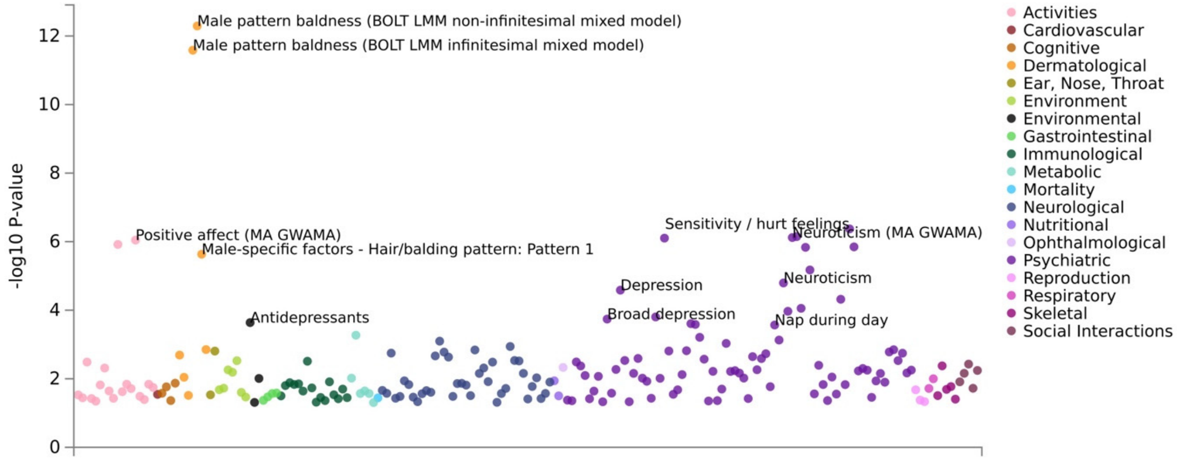

Supplementary Figure 7. PheWAS plots of novel SNPs for the mood disorders common factor
PheWAS plots were produced using GWAS Atlas.

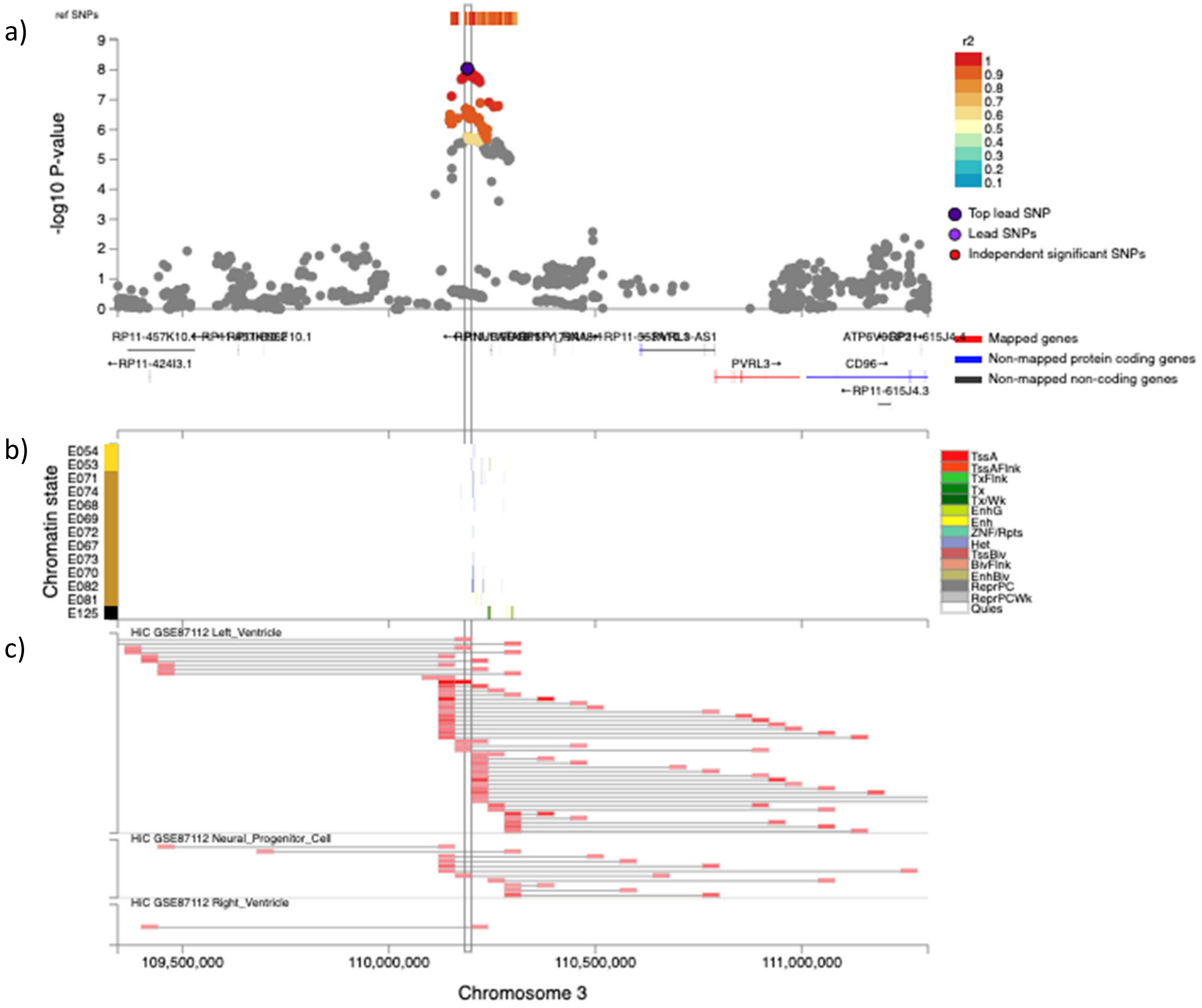

Supplementary Figure 8. Regional annotation plot for rs7652704, a novel SNP identified by the European ancestry mood/anxiety disorders GWAS.
(a) rs7652704 (in purple), its linked SNPs, and their position relative to genes. rs7652704's predicted genomic target *PVRL3* (*NECTIN3*) is shown in red. **(b)** Colocalization of rs7652704 with ROADMAP 15 core chromatin states (right-hand key) in 15 brain tissues (left hand key). E054 = ganglion eminence-derived neurospheres, E053 = cortex-derived neurospheres, E071 = hippocampus, E074 = substantia nigra, E068 = anterior caudate, E069 = cingulate gyrus, E072 = inferior temporal lobe, E067 = angular gyrus, E073 = dorsolateral prefrontal cortex, E070 = germinal matrix, E082 = female fetal brain, E081 = fetal male brain, E125 = NH-A astrocytes. TssA = Active Transcription Start Site, TsAFlnk = flanking active TSS, TxFlnk = transcribed at gene 5' and 3', Tx = strong transcription, TxWk = weak transcription, EnhG = genic enhancers, Enh = enhancers, ZNF/Rpts = ZNF genes and repeats, Het = heterochromatin, TssBiv = bivalent/poised TSS, BivFlnk = Flanking bivalent TSS/Enh, EnhBiv = bivalent enhancer, ReprPC = repressed PolyComb, PreprPCWk = weak repressed PolyComb, Quies = quiescent/low. **(c)** Colocalization with Hi-C signal in brain tissues. Each line represents an interaction, with the two red regions representing the loci which make contact.

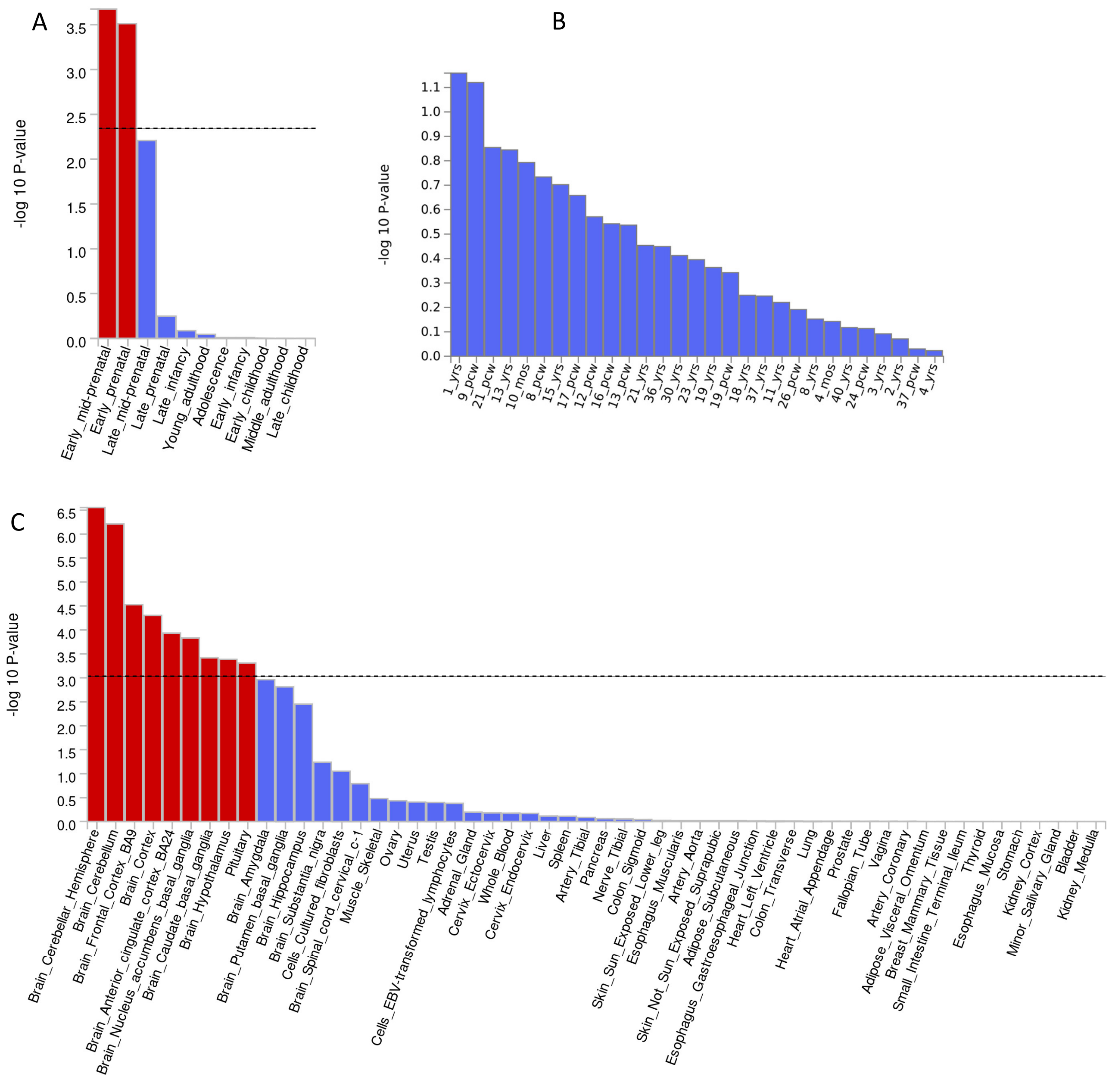

Supplementary Figure 9. Results of MAGMA tissue expression analysis of EUR substance use disorders factor

Results for the BrainSpan database are shown in panels A and B, and results for GTEx v8 are shown in Panel C. Dashed line represents significance threshold.

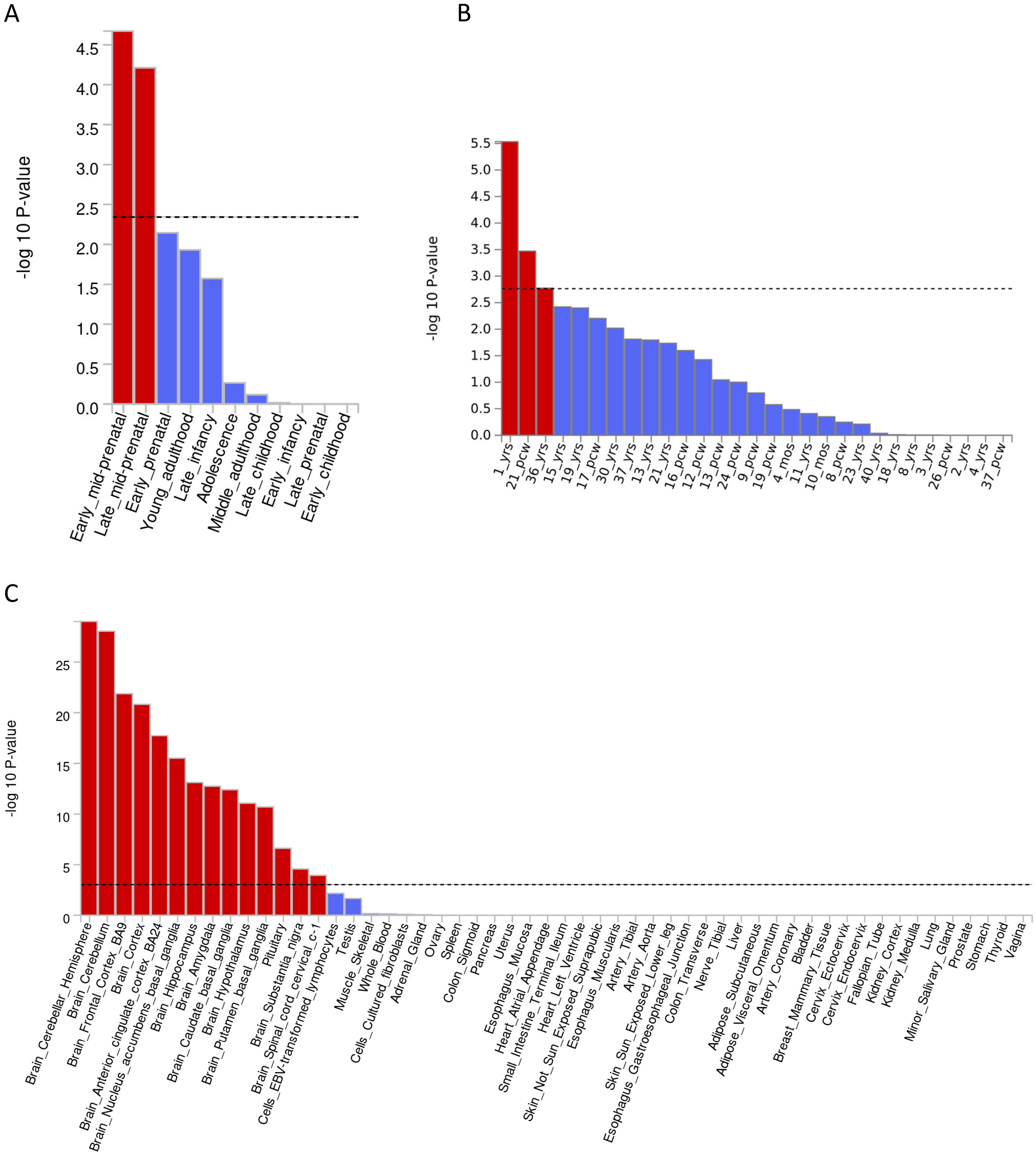

Supplementary Figure 10. Results of MAGMA tissue expression analysis of EUR psychotic disorders factor

Results for the BrainSpan database are shown in panels A and B, and results for GTEx v8 are shown in Panel C. Dashed line represents significance threshold.

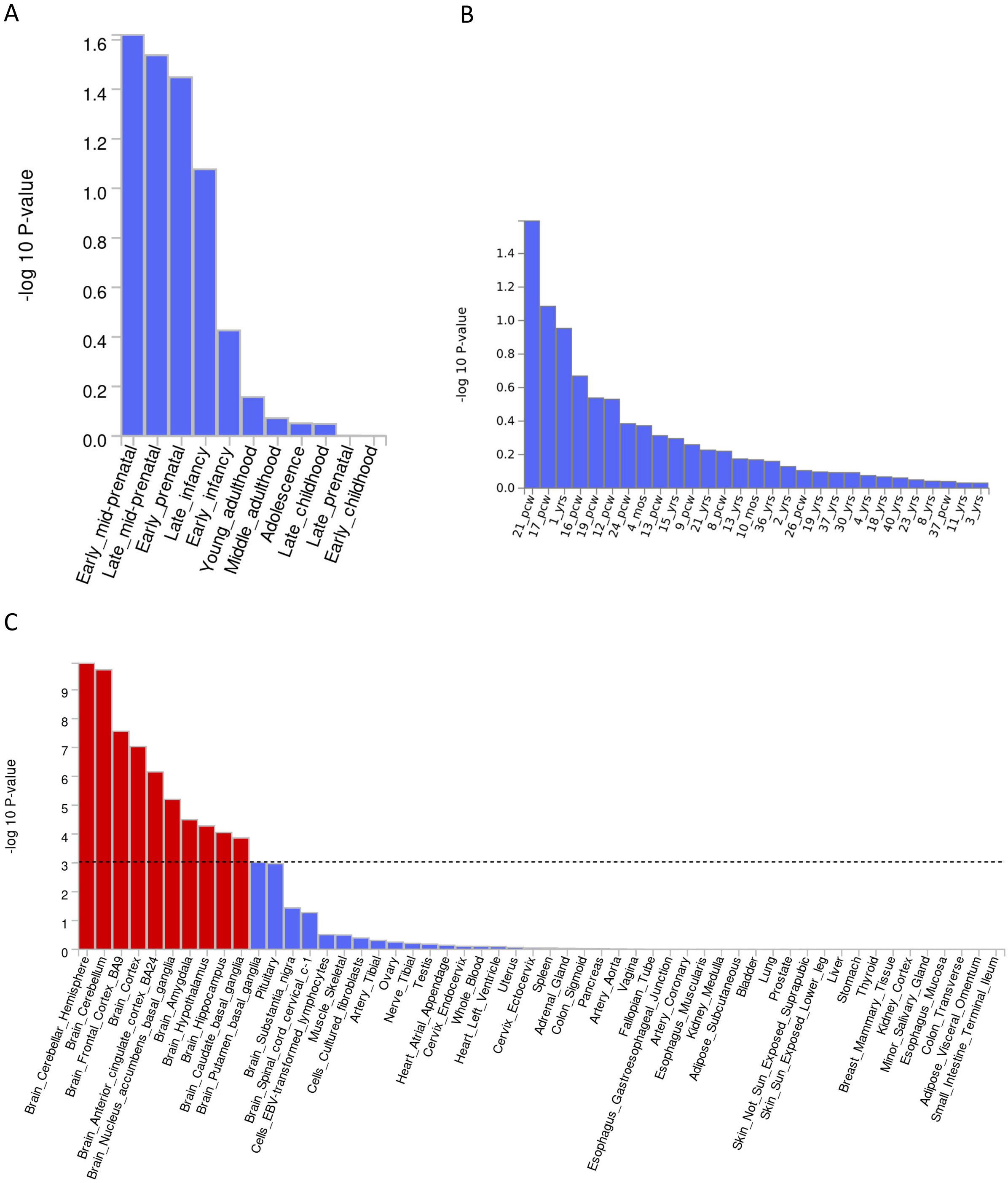

Supplementary Figure 11. Results of MAGMA tissue expression analysis of EUR mood disorders factor

Results for the BrainSpan database are shown in panels A and B, and results for GTEx v8 are shown in Panel C. Dashed line indicates significance threshold.

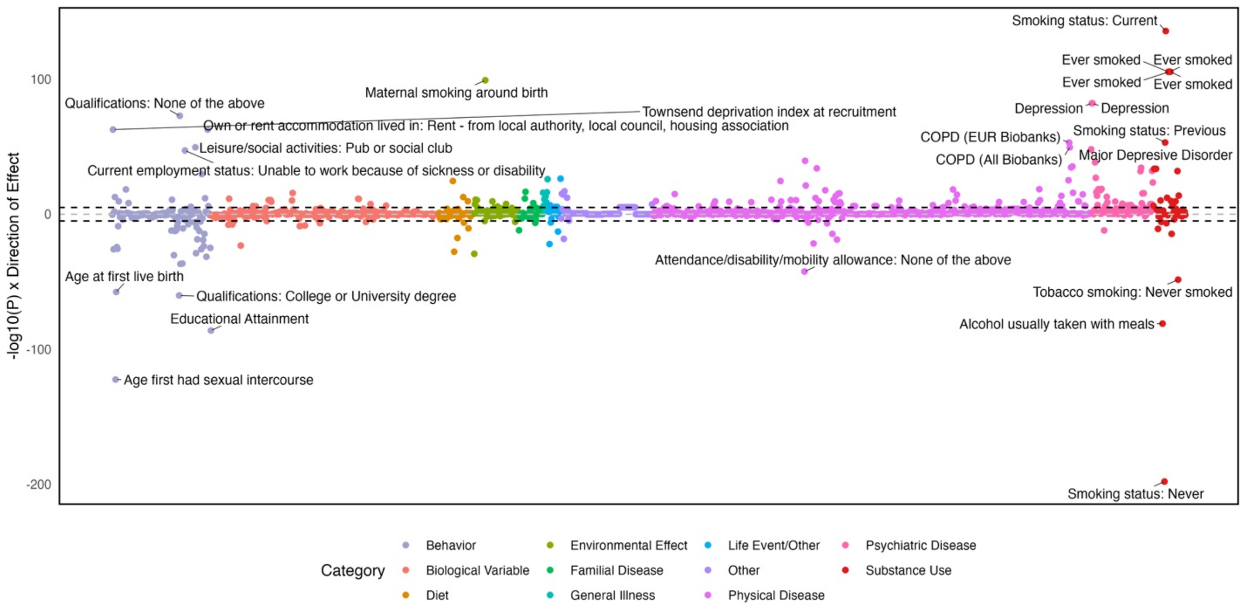

Supplementary Figure 12. Genetic correlation results for the EUR substance use disorders factor

The top 25 associations are shown. Assocation analyses were performed using the MASSIVE pipeline.

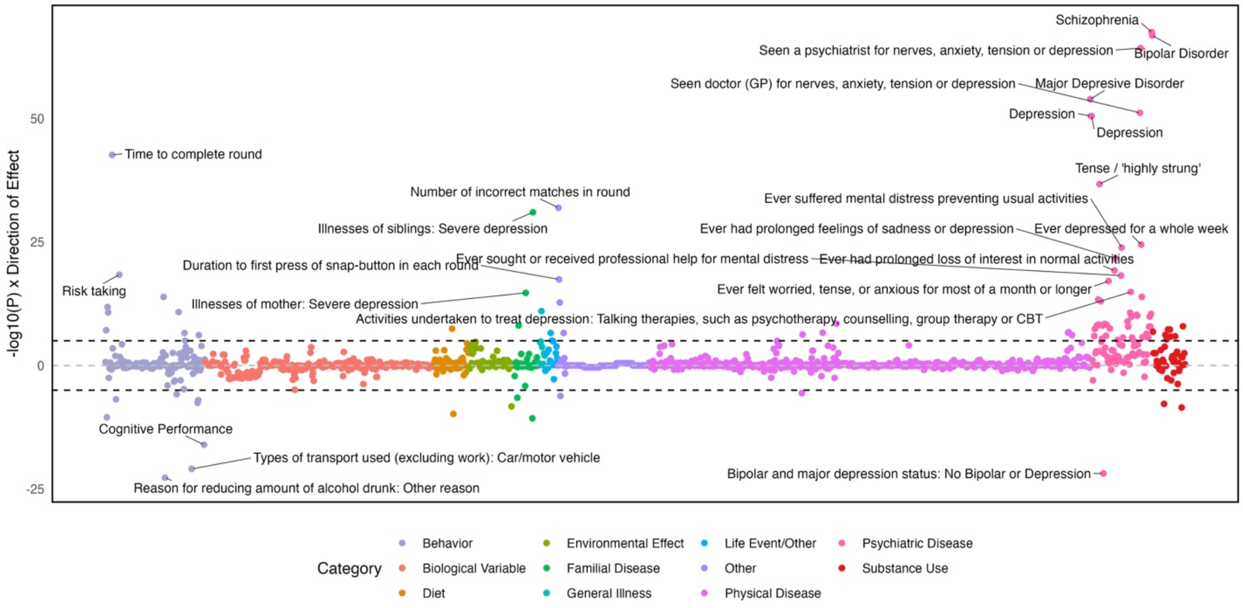

Supplementary Figure 13. Genetic correlation results for the EUR psychotic disorders factor

The top 25 associations are shown. Assocation analyses were performed using the MASSIVE pipeline.

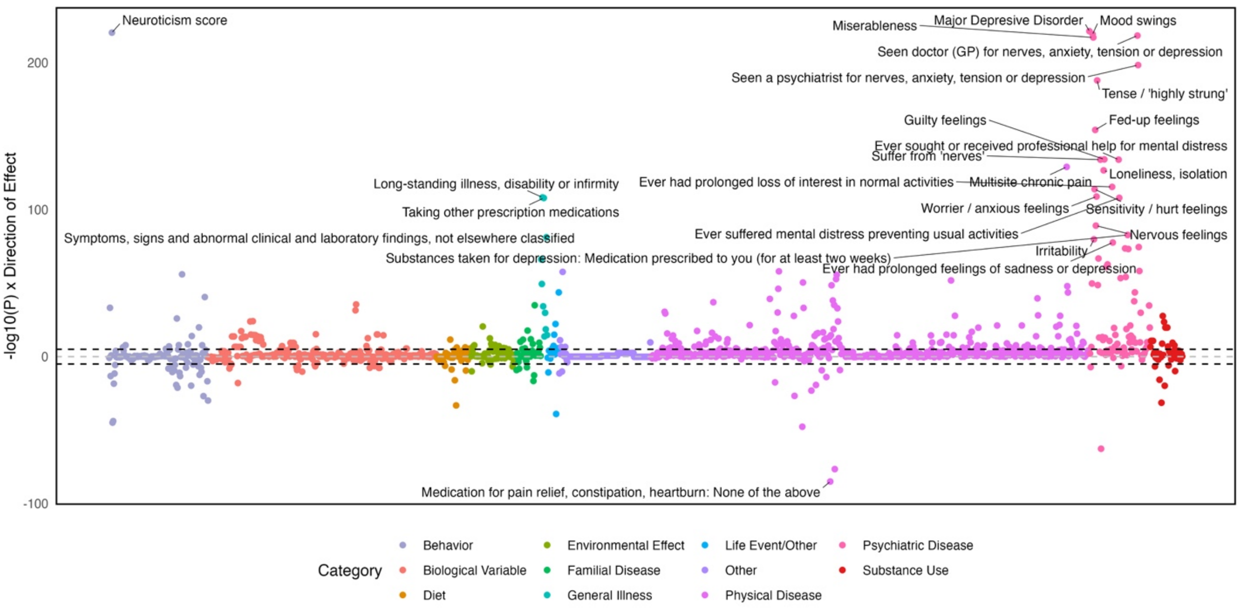

Supplementary Figure 14. Genetic correlation results for the EUR mood disorders factor
The top 25 associations are shown. Assocation analyses were performed using the MASSIVE pipeline.

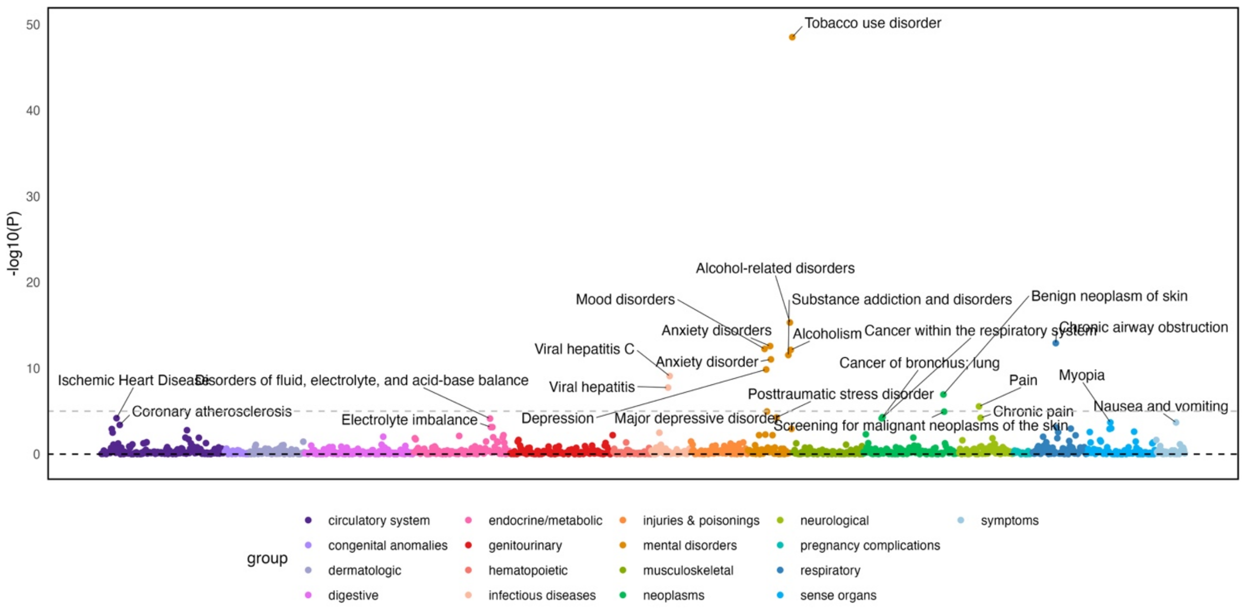

Supplementary Figure 15. PheWAS results for the EUR substance use disorders factor in Penn Medicine BioBank

The top 25 associations are shown. All p-values were adjusted using Benjamini-Hochberg false discovery rate (FDR) correction.

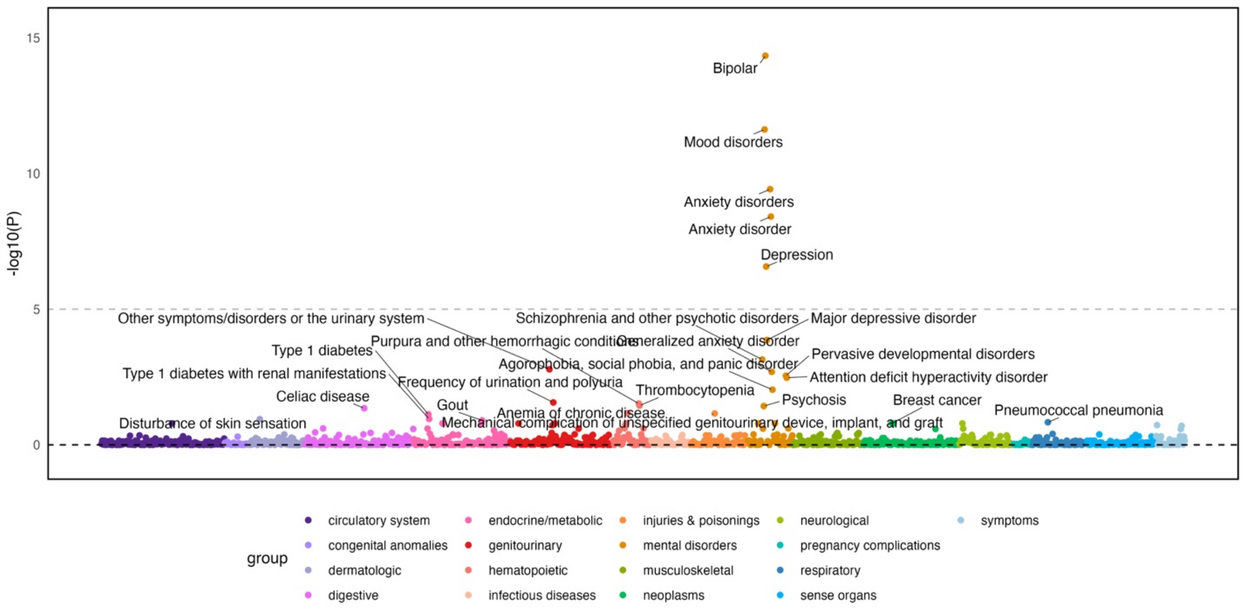

Supplementary Figure 16. PheWAS results for the EUR psychotic disorders factor in Penn Medicine BioBank
The top 25 associations are shown. All p-values were adjusted using Benjamini-Hochberg false discovery rate (FDR) correction.

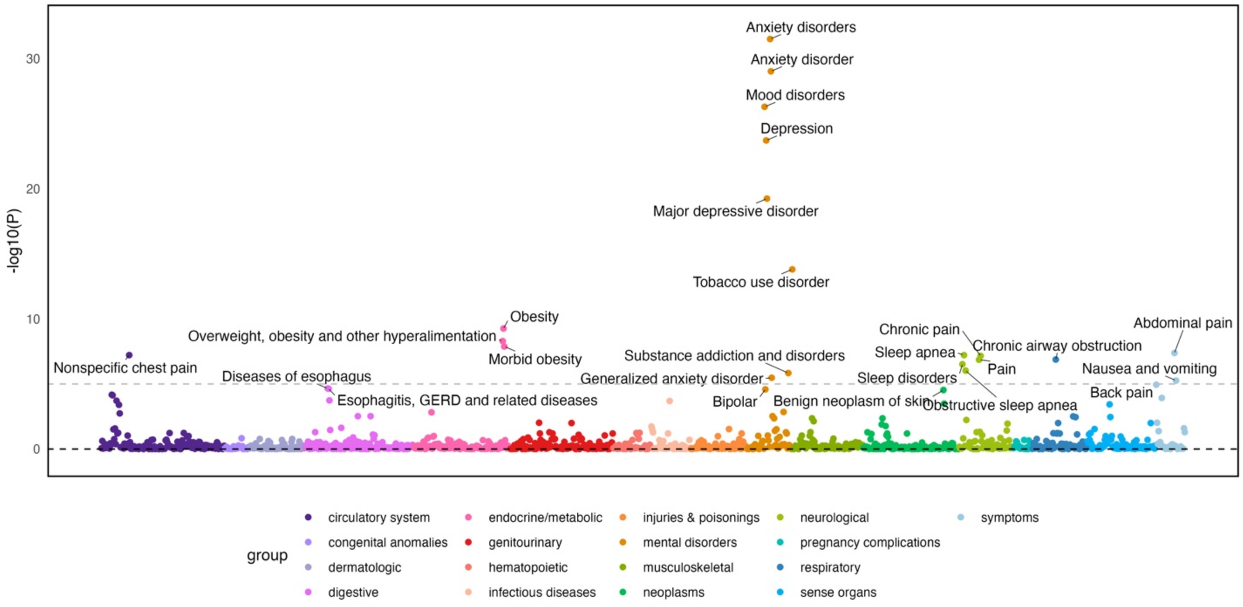

Supplementary Figure 17. PheWAS results for the EUR mood disorders factor in Penn Medicine BioBank

The top 25 associations are shown. All p-values were adjusted using Benjamini-Hochberg false discovery rate (FDR) correction.

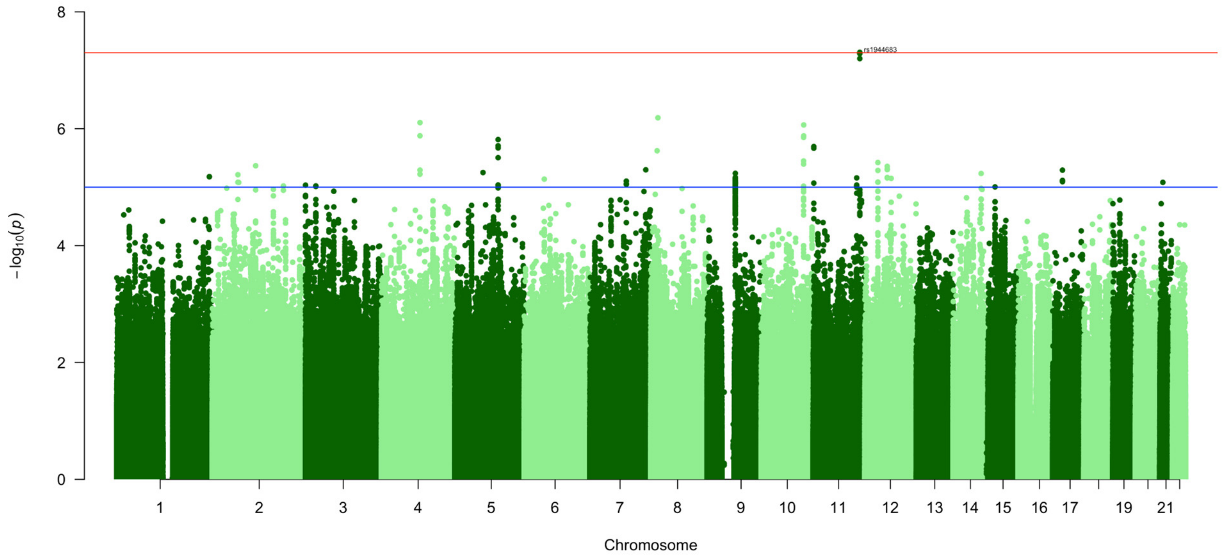

Supplementary Figure 18. Manhattan plot for substance use disorders factor in AFR ancestry individuals
GWAS identified 1 lead genome-wide significant SNP, rs1944683.

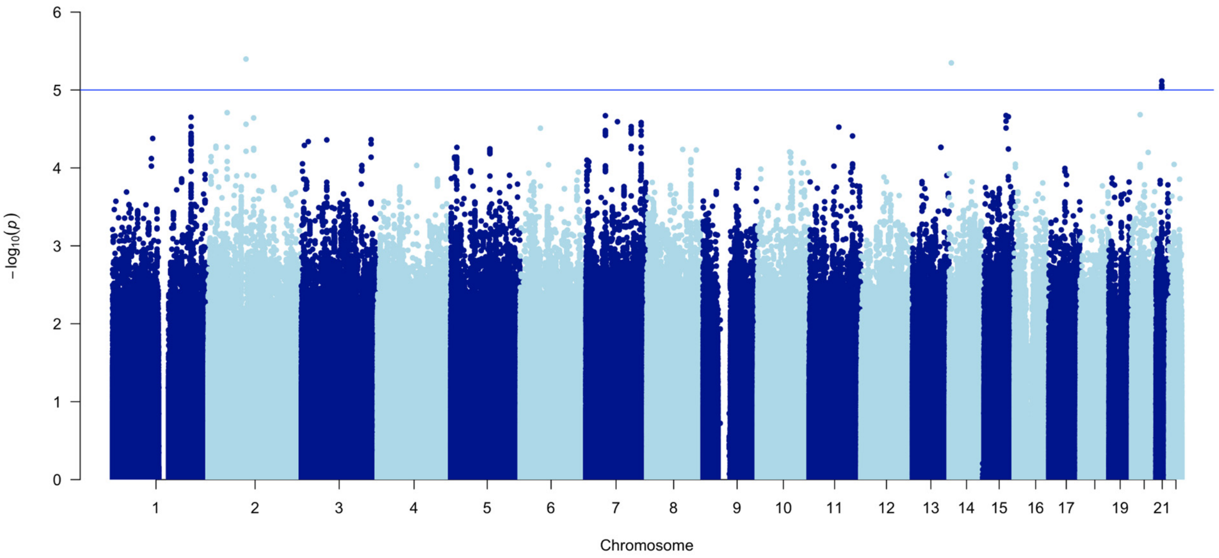

Supplementary Figure 19. Manhattan plot for psychiatric disorders factor in AFR ancestry individuals

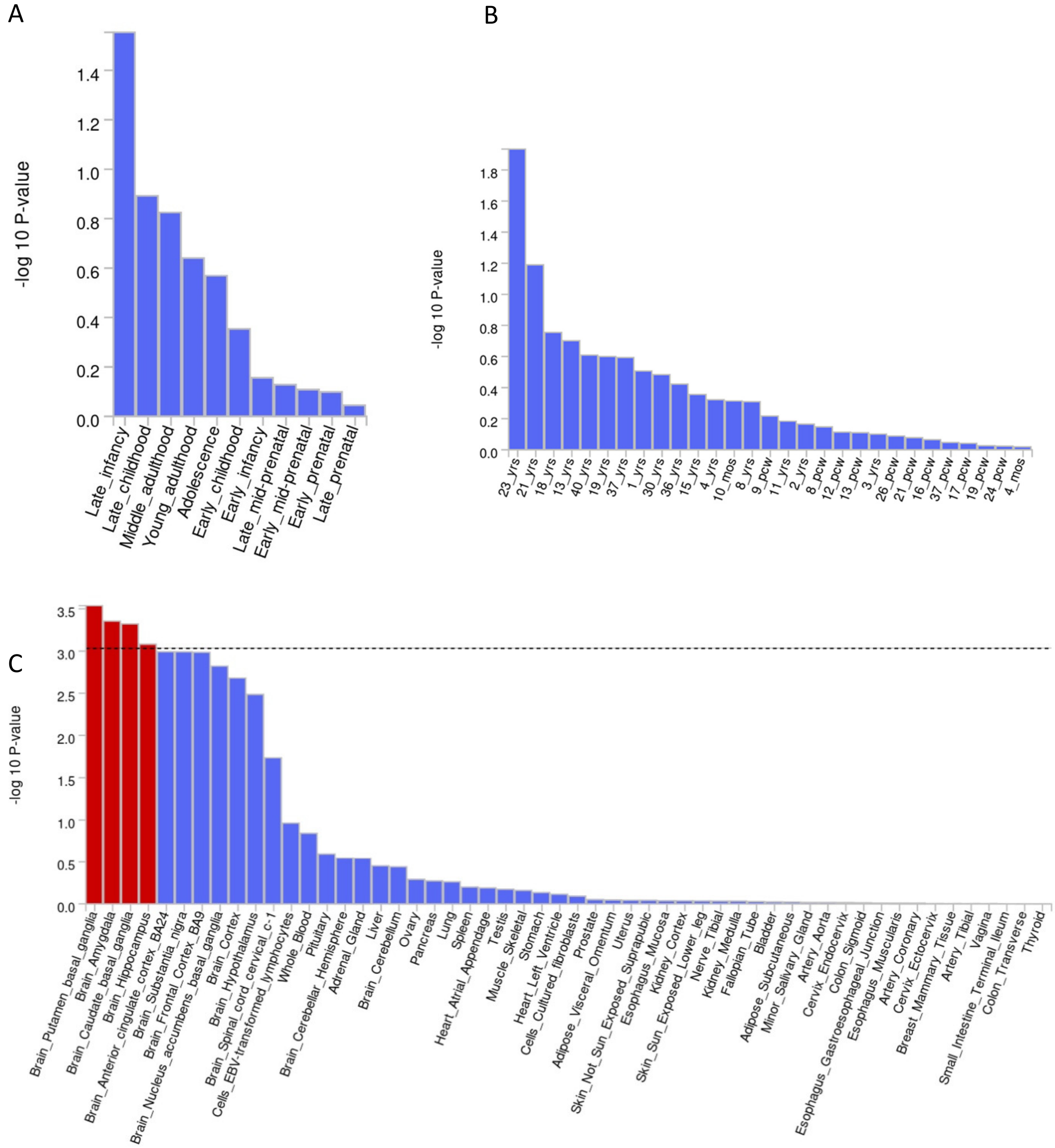

Supplementary Figure 20. Results of MAGMA tissue expression analysis of AFR ancestry substance use disorders factor

Results for the BrainSpan database are shown in panels A and B, and results for GTEx v8 are shown in Panel C. Dashed line indicates significance threshold.

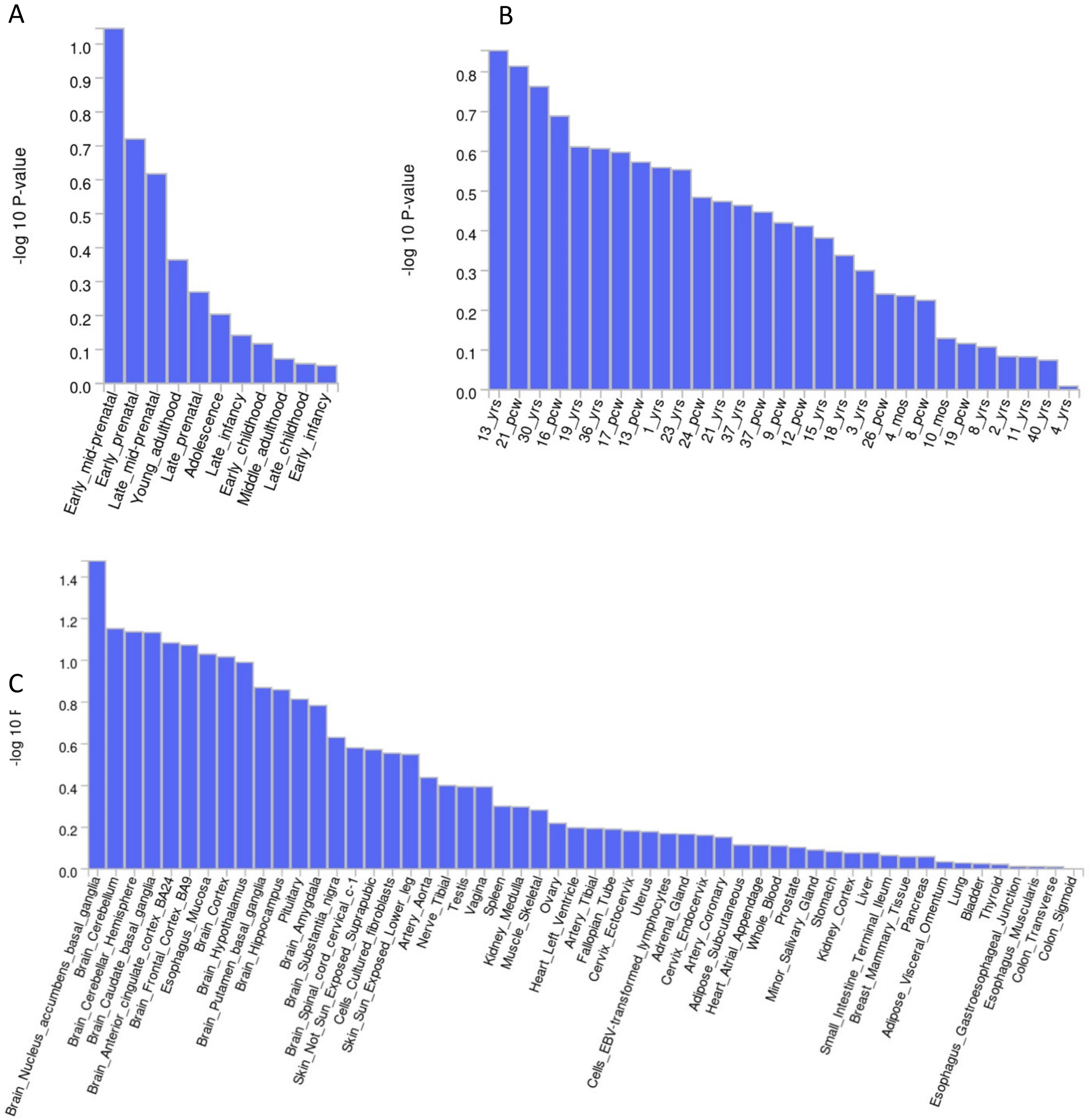

Supplementary Figure 21. Results of MAGMA tissue expression analysis of AFR ancestry psychiatric disorders factor

Results for the BrainSpan database are shown in panels A and B, and results for GTEx v8 are shown in Panel C.

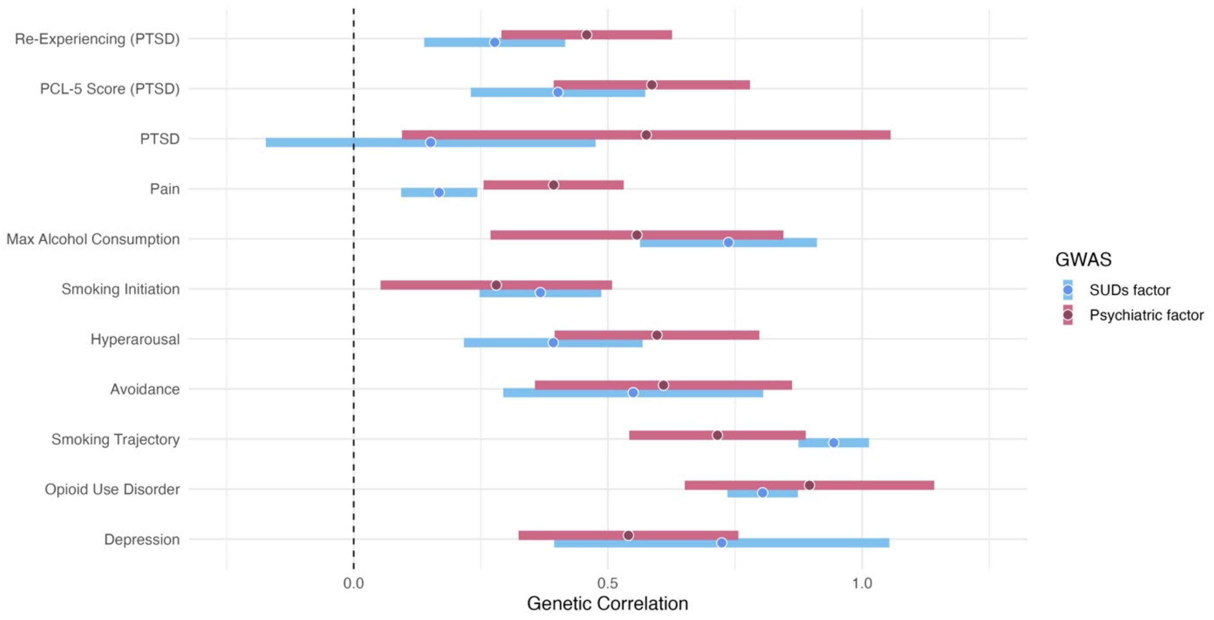

Supplementary Figure 22. Genetic correlations between AFR ancestry common factors and psychiatric and substance use phenotypes

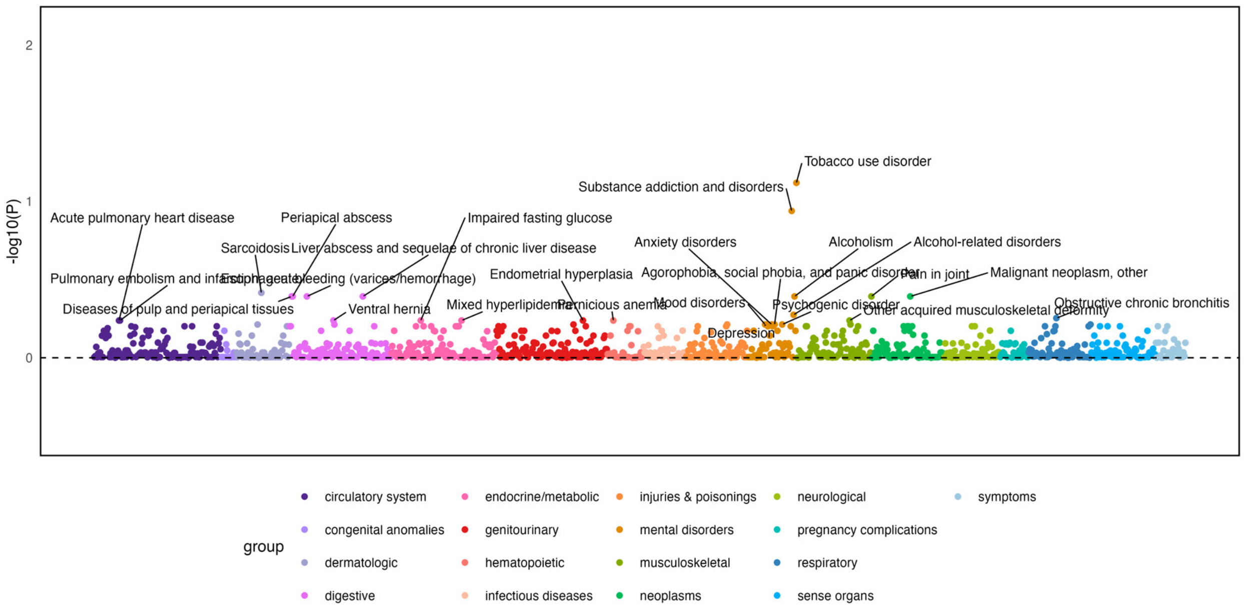

Supplementary Figure 23. PheWAS results for AFR ancestry substance use disorders factor in Penn Medicine BioBank

The top 25 associations are shown. All p-values were adjusted using Benjamini-Hochberg false discovery rate (FDR) correction.

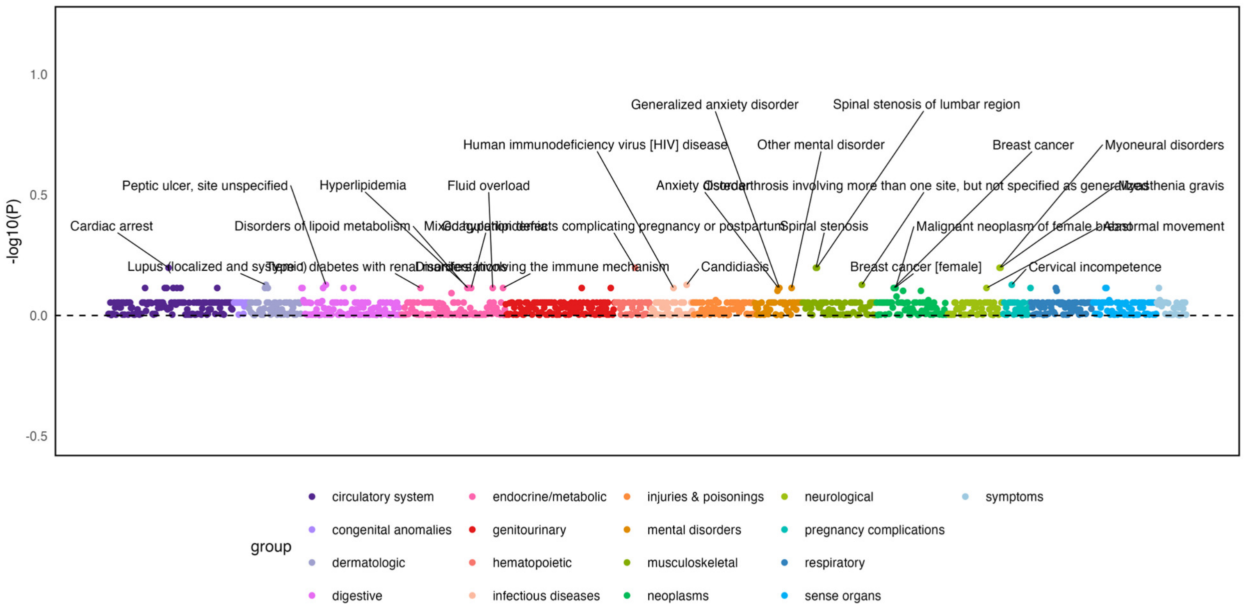

Supplementary Figure 24. PheWAS results for AFR ancestry psychiatric disorders factor in Penn Medicine BioBank

The top 25 associations are shown. All p-values were adjusted using Benjamini-Hochberg false discovery rate (FDR) correction.

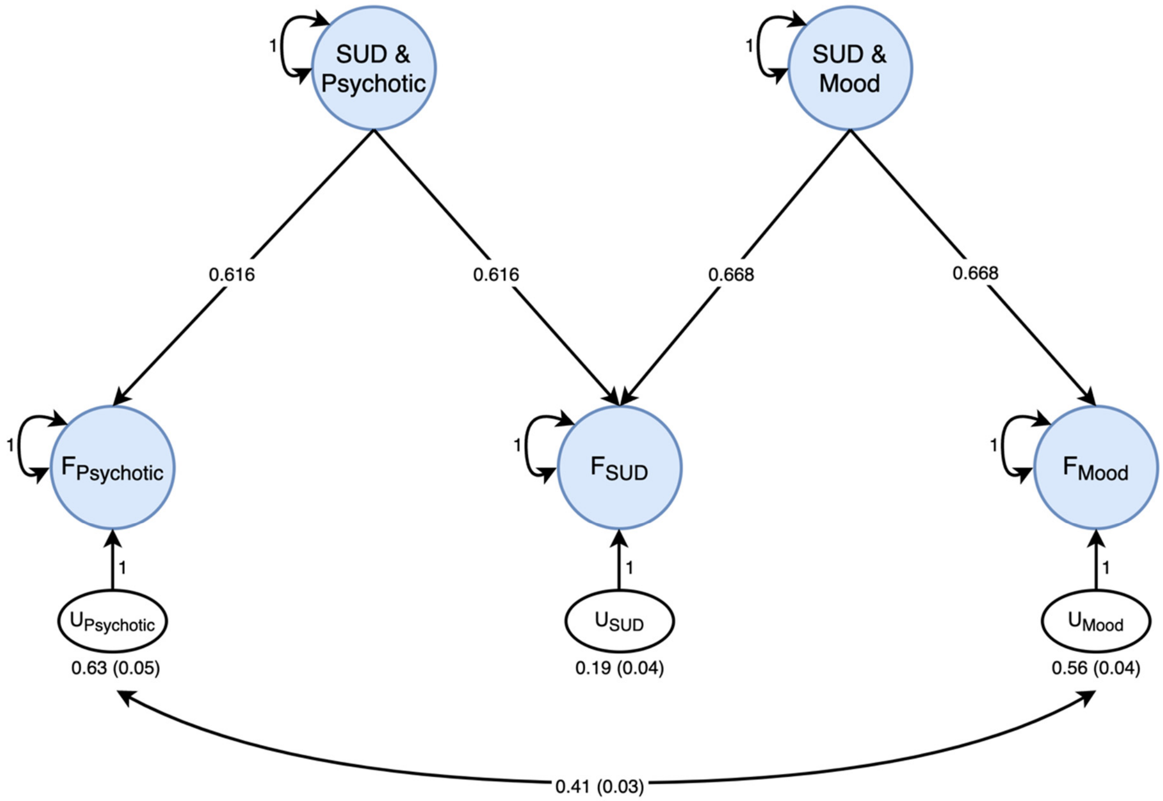

Supplementary Figure 25. EUR ancestry second order common factor model
Model fit statistics: $\chi2(2) = 57.61$, $p = 3.09*10^{-13}$, AIC = 65.61, CFI = 0.91, SRMR = 0.07.

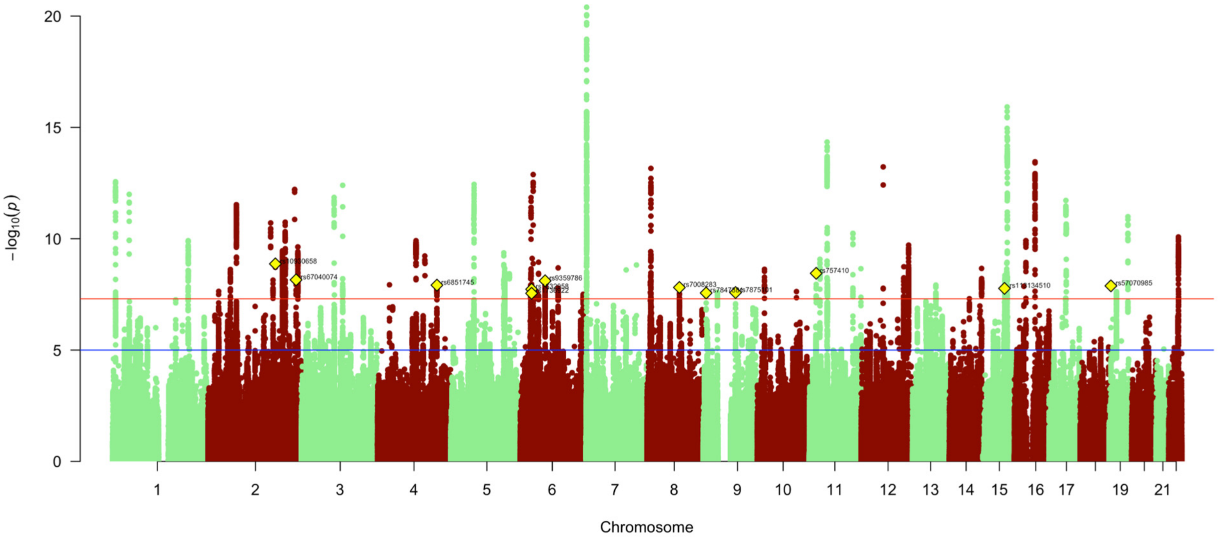

Supplementary Figure 26. Manhattan plot for second-order common factor representing overlap between substance use and psychotic disorders in EUR ancestry individuals
GWAS identified 76 lead SNPs, 12 of which were not identified by any of the input GWAS.

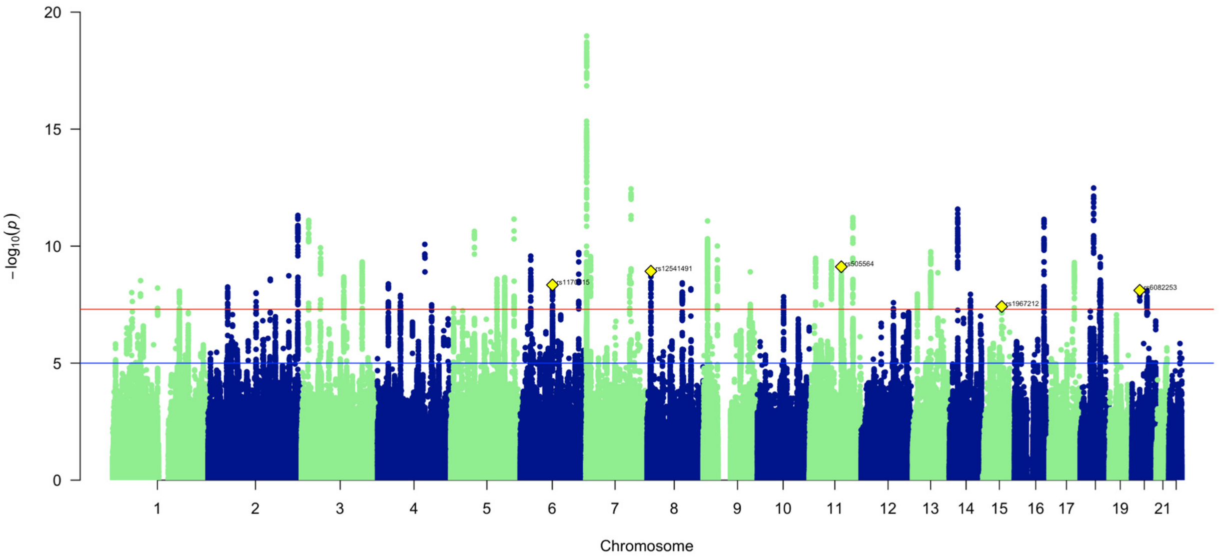

Supplementary Figure 27. Manhattan plot for second-order common factor representing overlap between substance use and mood/anxiety disorders in EUR ancestry individuals
GWAS identified 63 lead SNPs, 5 of which were not identified by any of the input GWAS.

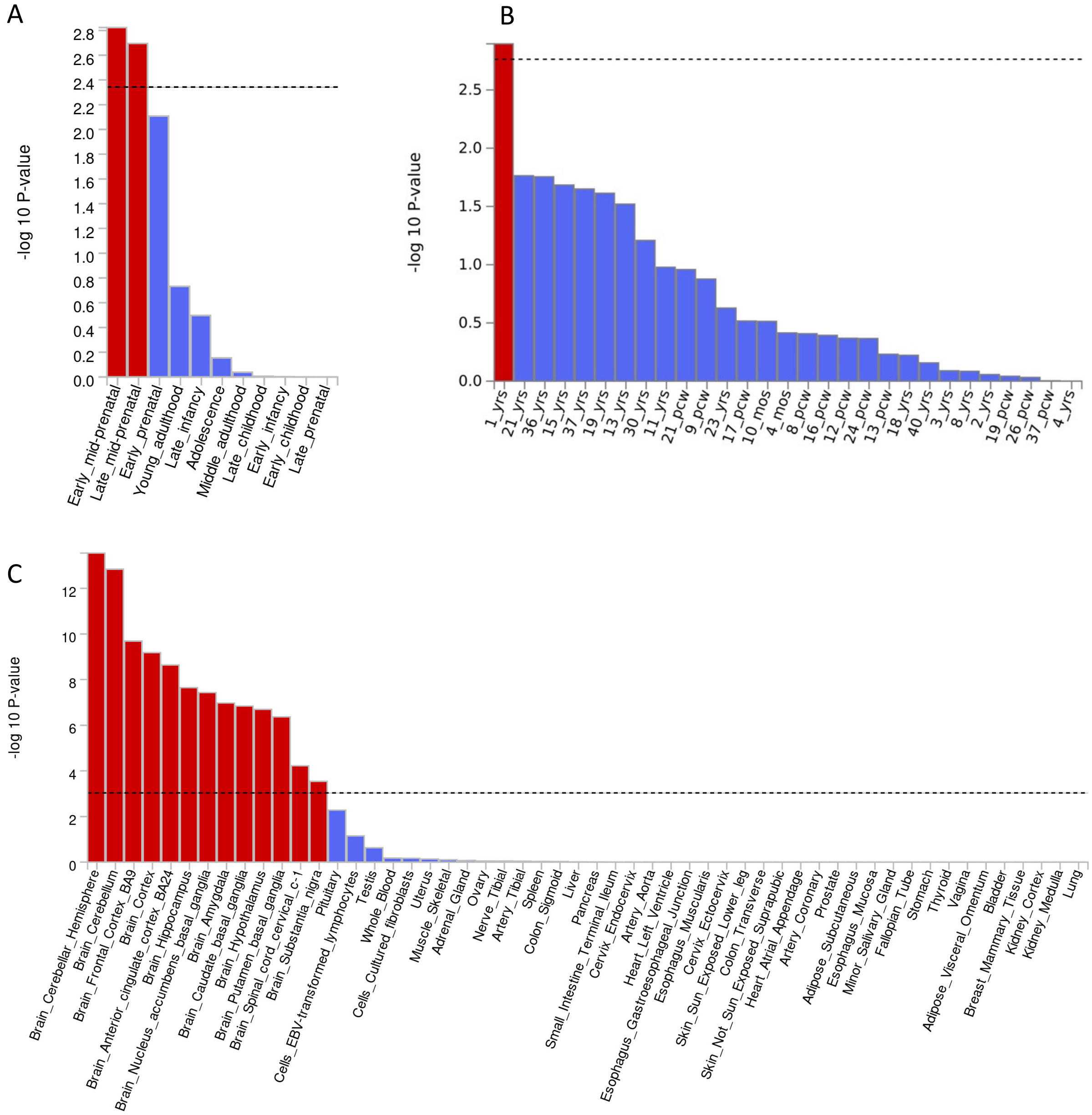

Supplementary Figure 28. Results of MAGMA tissue expression analysis of EUR ancestry second-order substance use and psychotic disorders factor

Results for the BrainSpan database are shown in panels A and B, and results for GTEx v8 are shown in Panel C. Dashed line indicates significance threshold.

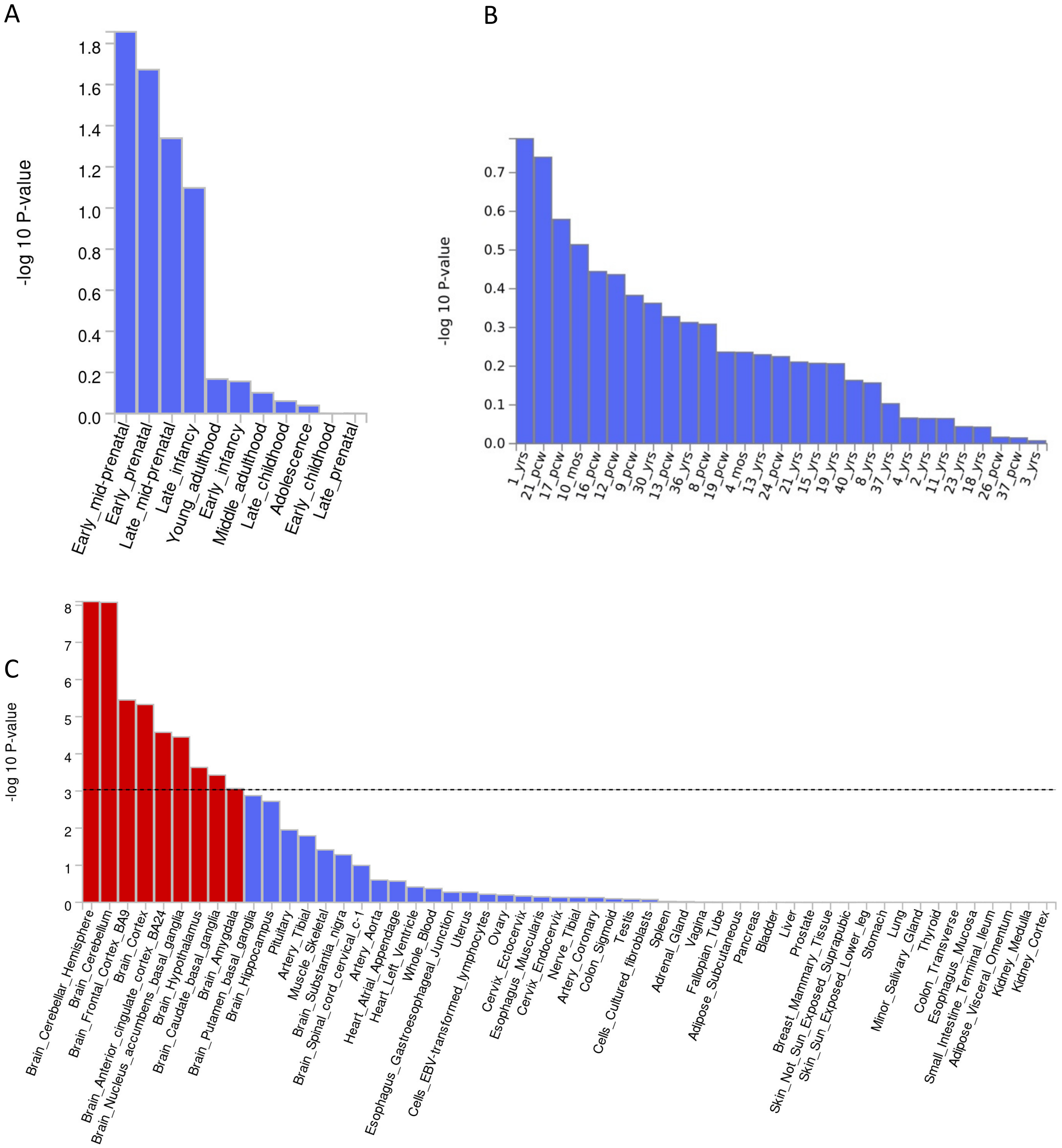

Supplementary Figure 29. Results of MAGMA tissue expression analysis of EUR ancestry second-order substance use and mood disorders factor

Results for the BrainSpan database are shown in panels A and B, and results for GTEx v8 are shown in panel C. Dashed line indicates significance threshold.

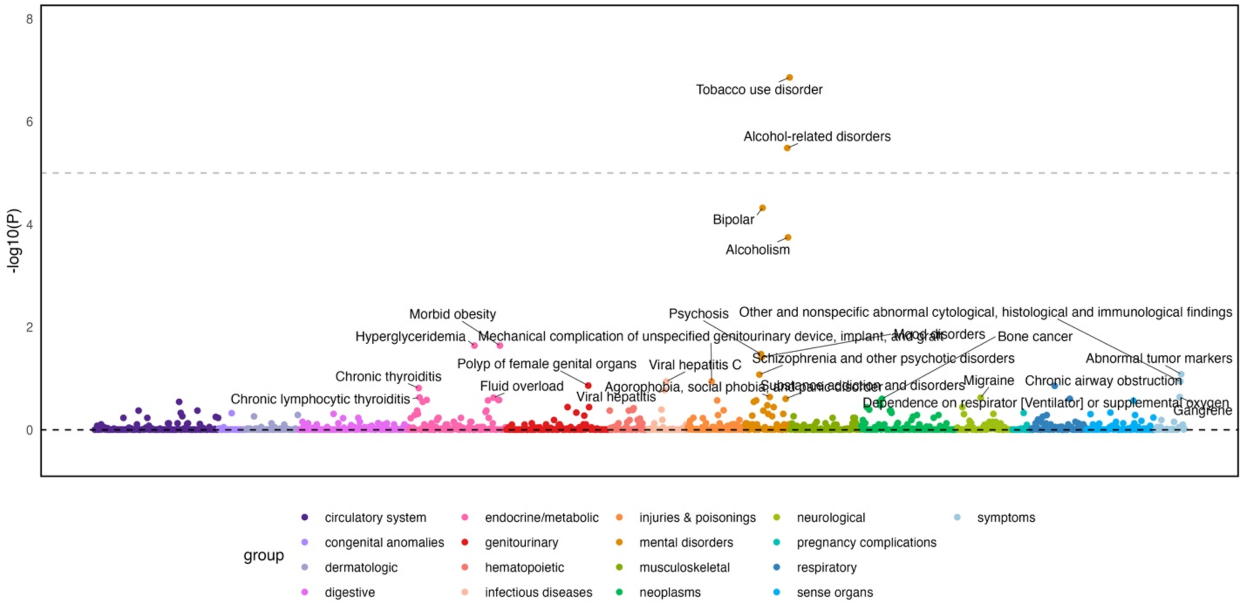

Supplementary Figure 30. PheWAS results for EUR ancestry second-order common factor representing overlap in substance use and psychotic disorders in Penn Medicine BioBank
The top 25 associations are shown. All p-values were adjusted using Benjamini-Hochberg false discovery rate (FDR) correction.

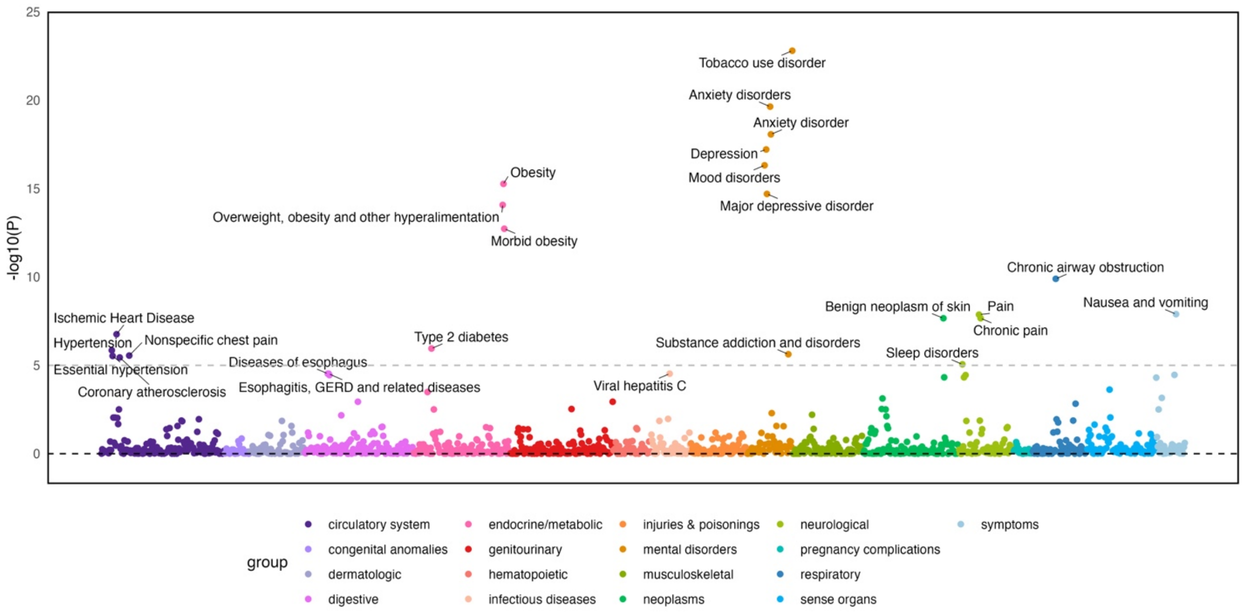

Supplementary Figure 31. PheWAS results for EUR ancestry second-order common factor representing overlap in substance use and mood/anxiety disorders in Penn Medicine BioBank

The top 25 associations are shown. All p-values were adjusted using Benjamini-Hochberg false discovery rate (FDR) correction.

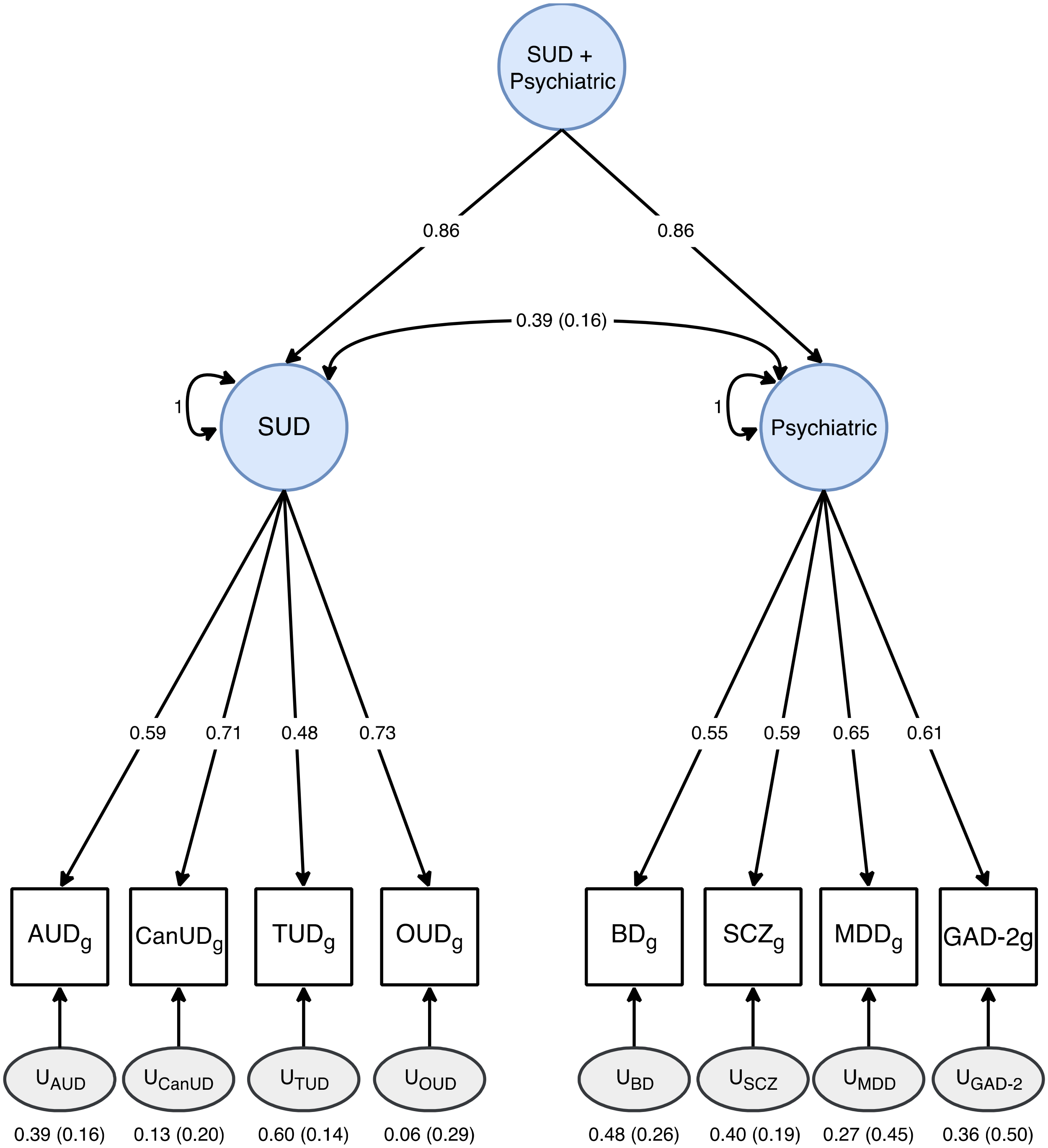

Supplementary Figure 32. AFR ancestry second order common factor model
Model fit statistics: $\chi 2(19) = 21.49$, $p = 0.31$, AIC = 55.49, CFI = 0.99, SRMR = 0.10.

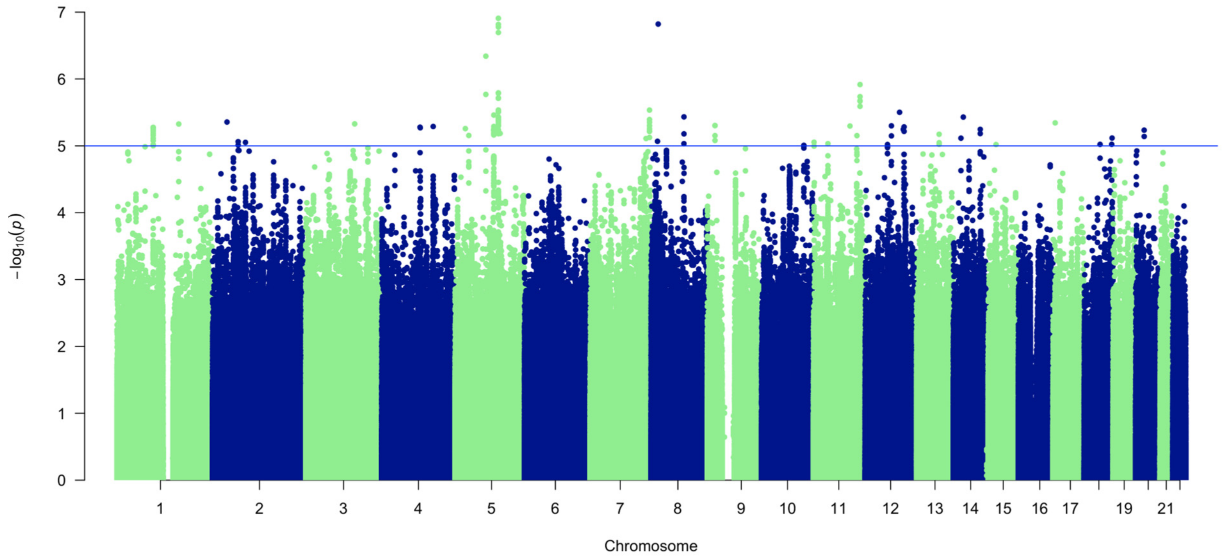

Supplementary Figure 33. Manhattan plot for second-order common factor representing overlap between substance use and psychiatric disorders in AFR ancestry individuals

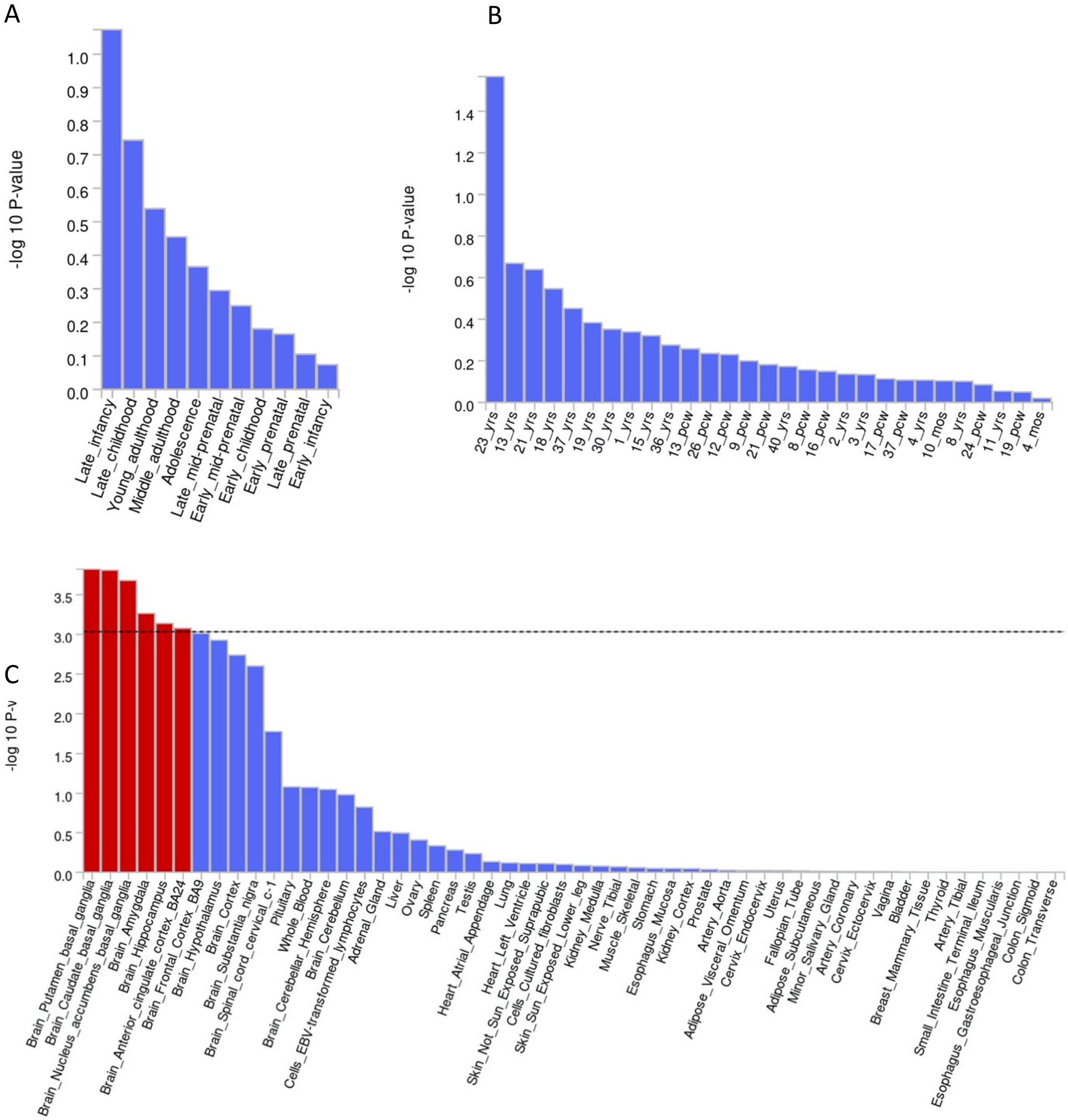

Supplementary Figure 34. Results of MAGMA tissue expression analysis of AFR ancestry second-order substance use and psychiatric disorders factor

Results for the BrainSpan database are shown in panels A and B, and results for GTEx v8 are shown in panel C. Dashed line indicates significance threshold.

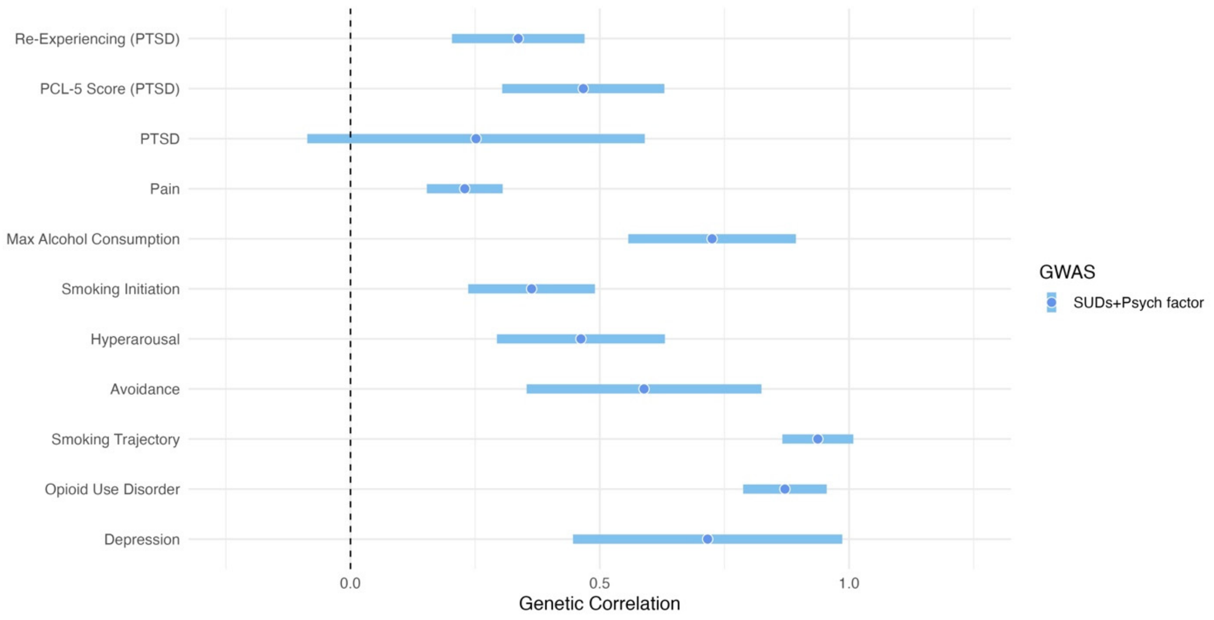

Supplementary Figure 35. Genetic correlations between the AFR ancestry second-order common factor and psychiatric and substance use traits

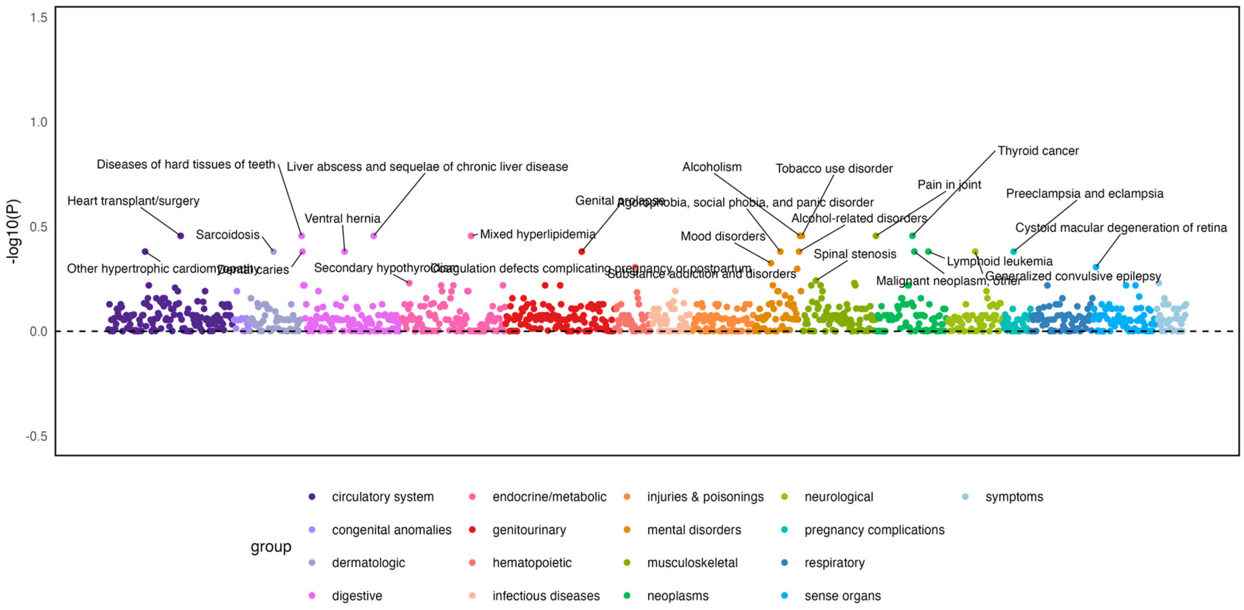

Supplementary Figure 36. PheWAS results for AFR ancestry second-order common factor representing overlap in substance use and psychiatric disorders in Penn Medicine BioBank
The top 25 associations are shown. All p-values were adjusted using Benjamini-Hochberg false discovery rate (FDR) correction.

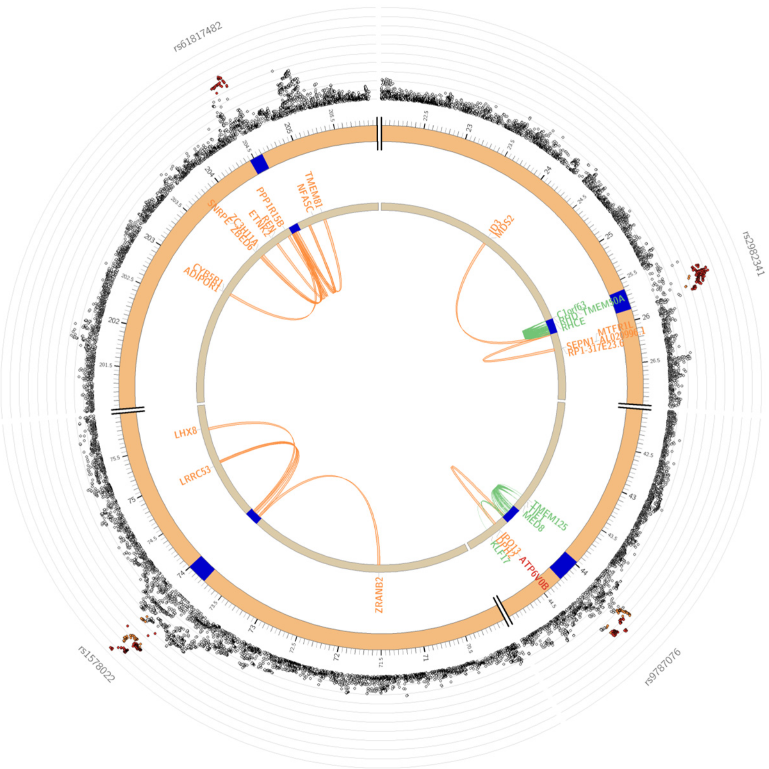

Chromosome 1

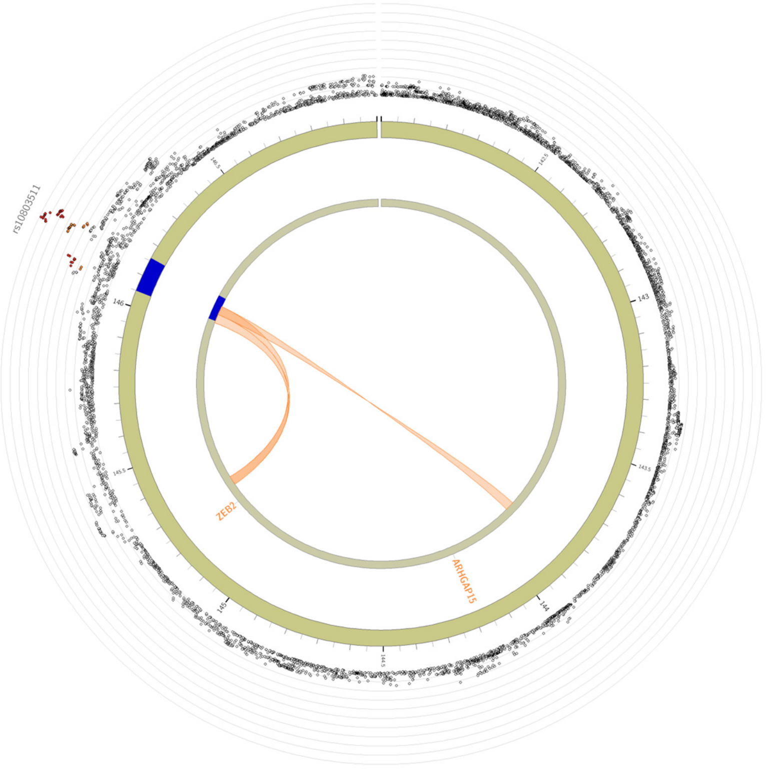

Chromosome 2

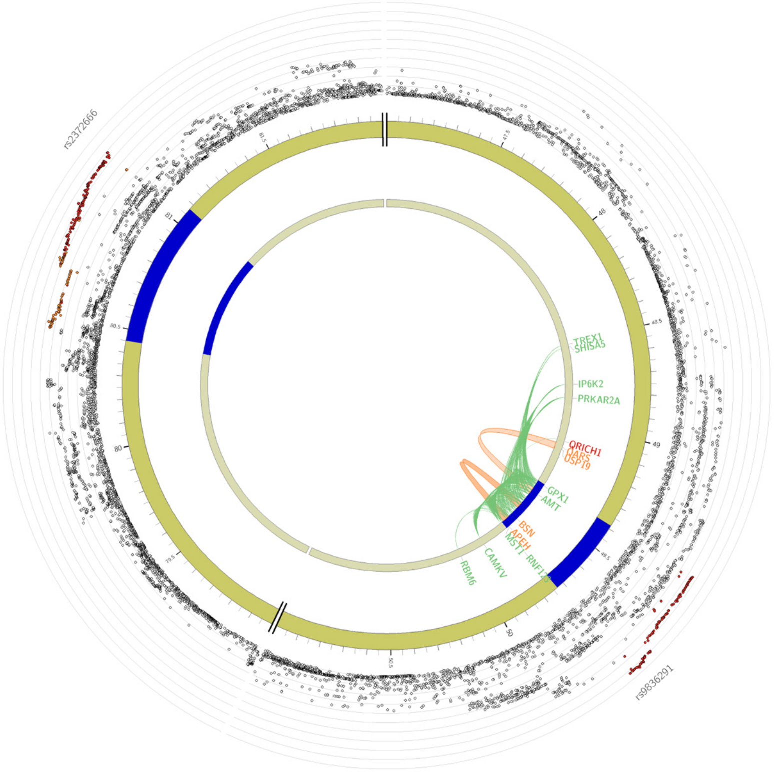

Chromosome 3

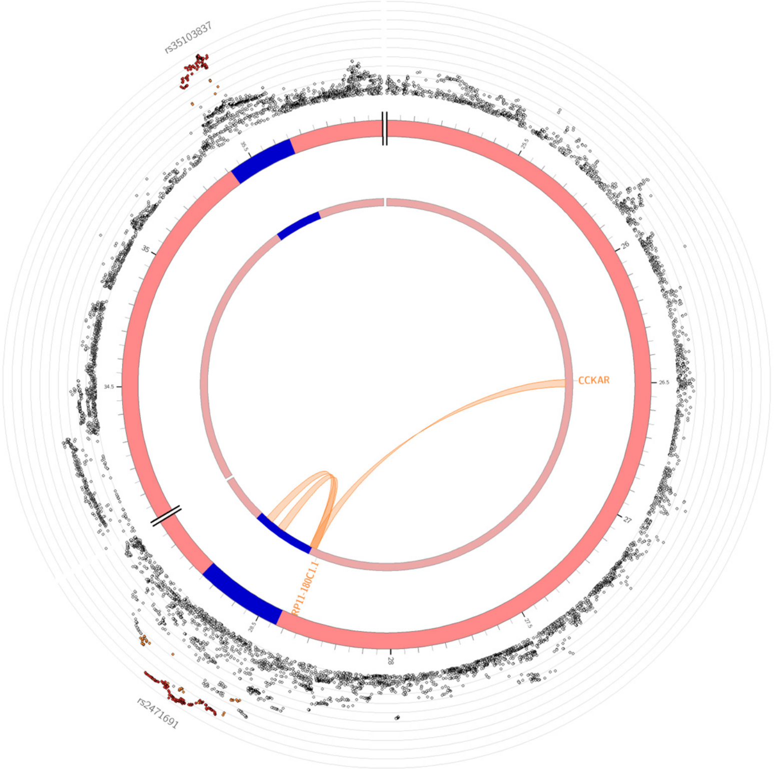

Chromosome 4

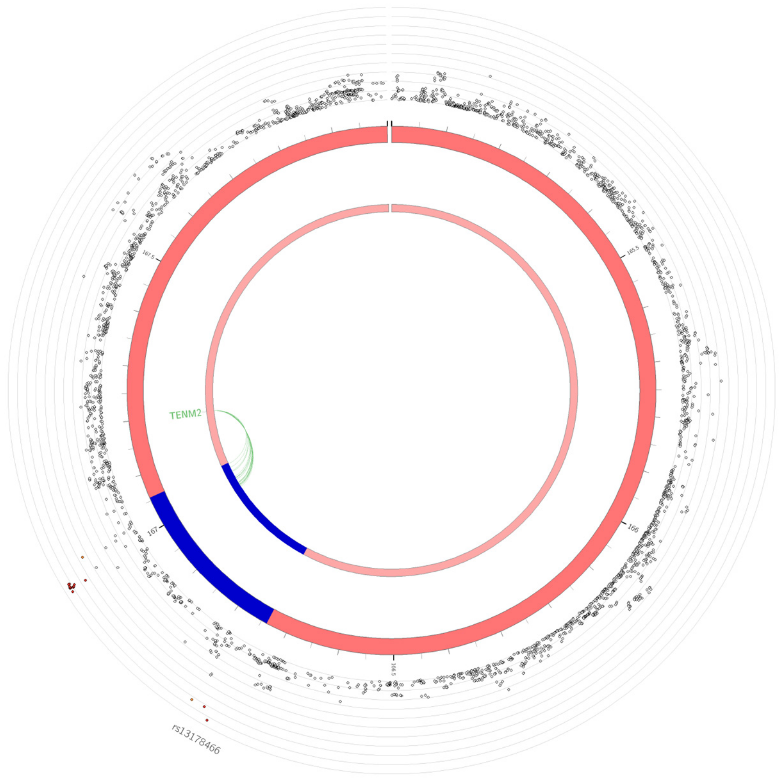

Chromosome 5

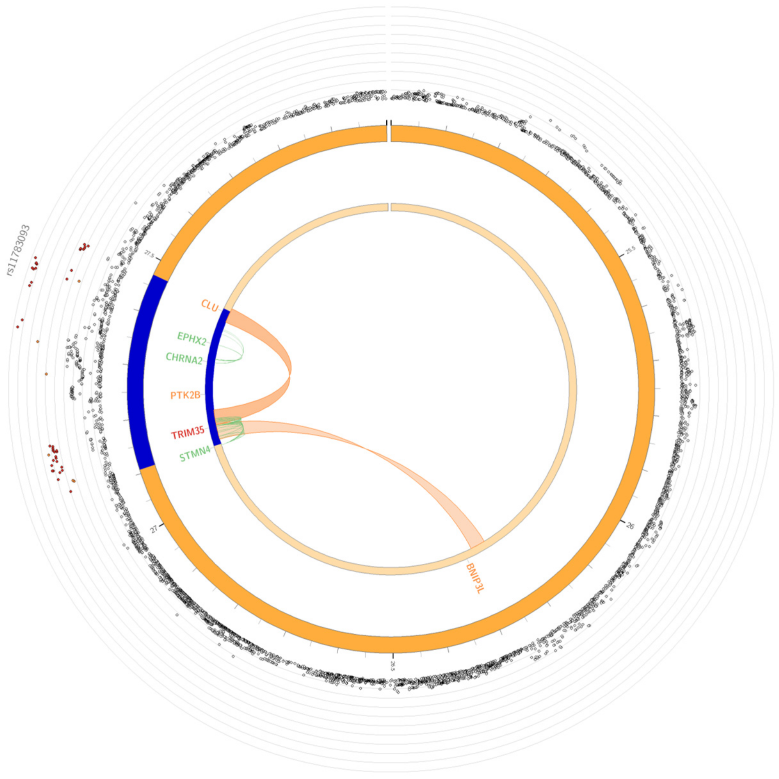

Chromosome 8

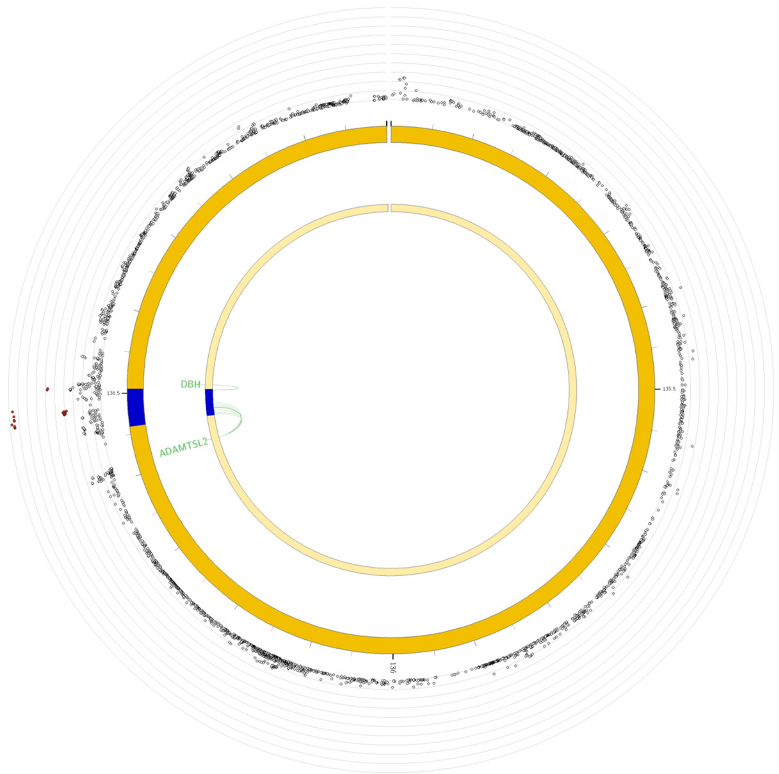

Chromosome 9

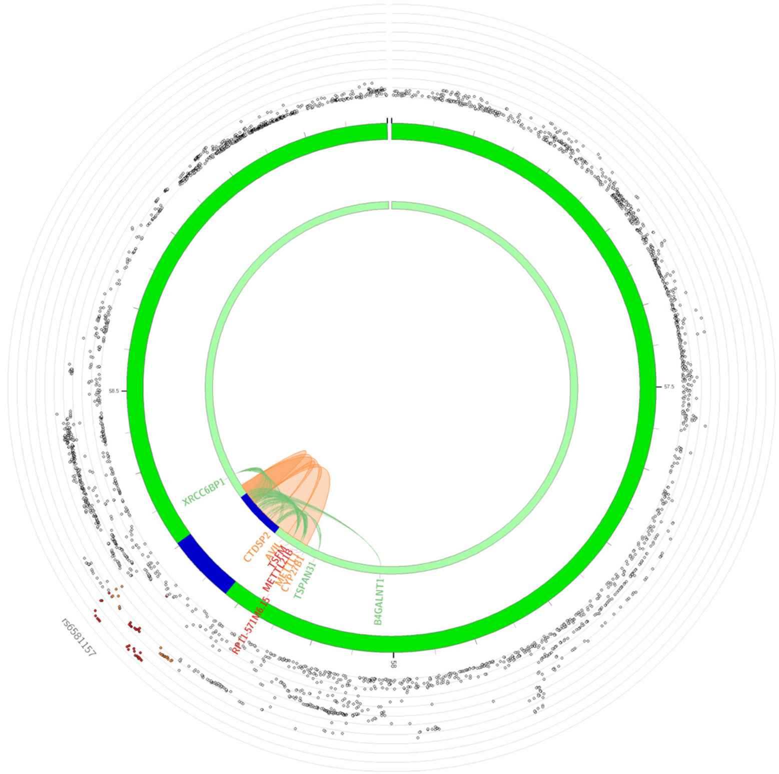

Chromosome 12

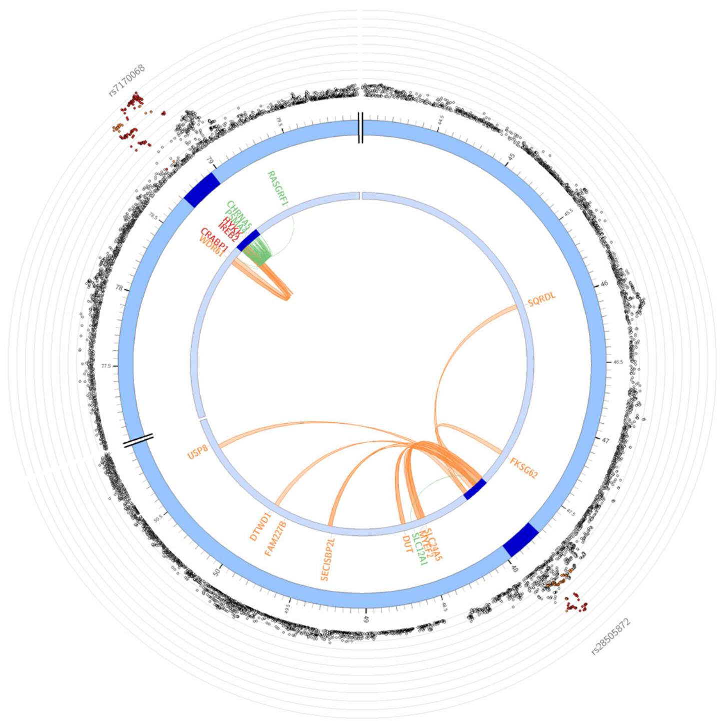

Chromosome 15

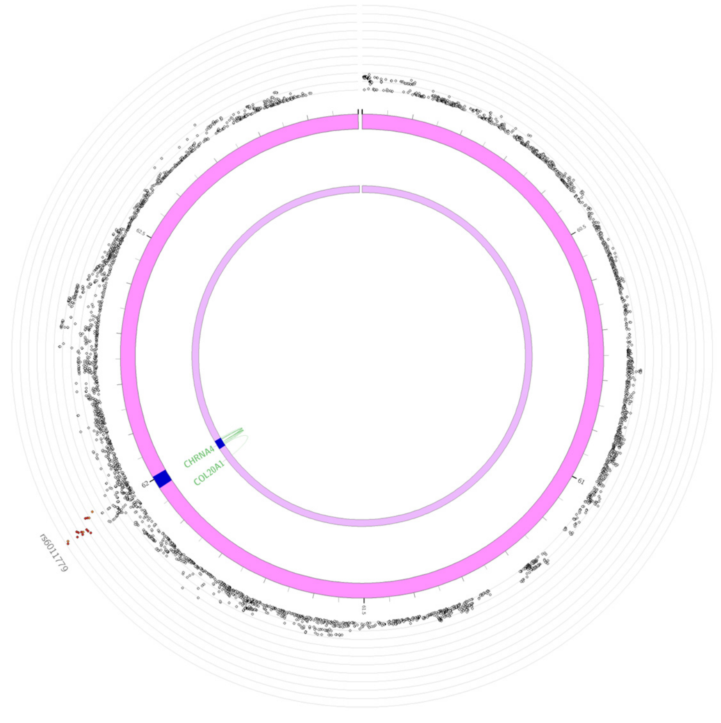

Chromosome 20

Supplementary Figure 37. Significant SNPs identified in TUD Independent GWAS
The chromosome is depicted as a circle with SNPs plotted by their -log10(p-value), and lead SNPs annotated. Orange links indicate chromatin contact, green links indicate cis-eQTLs, and red links indicate both.

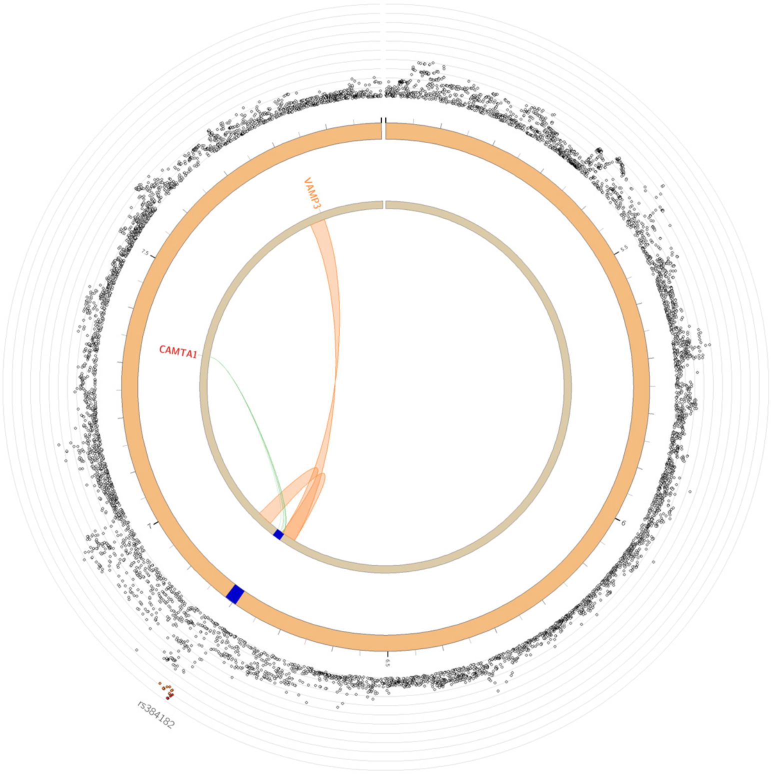

Chromosome 1

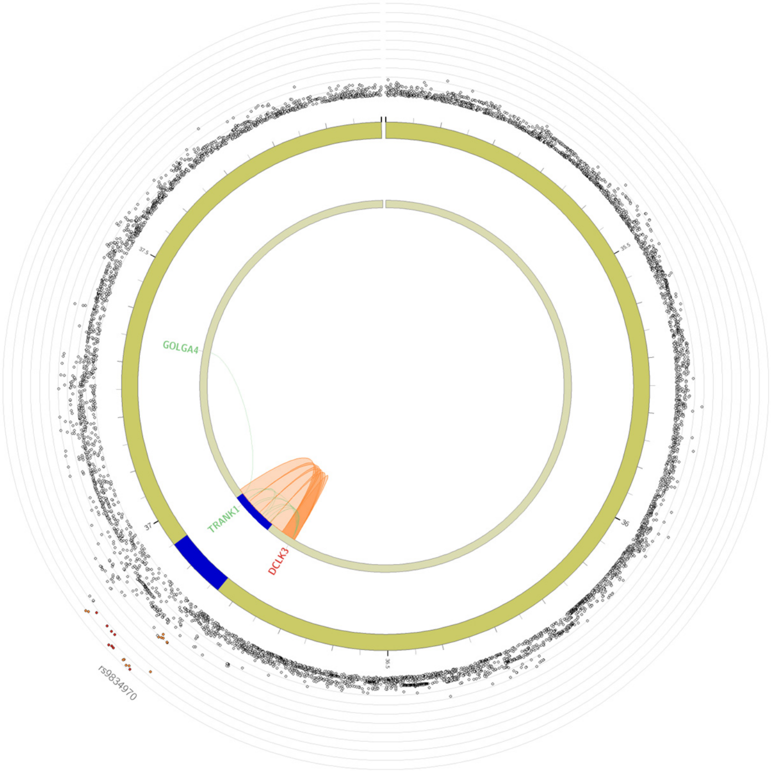

Chromosome 3

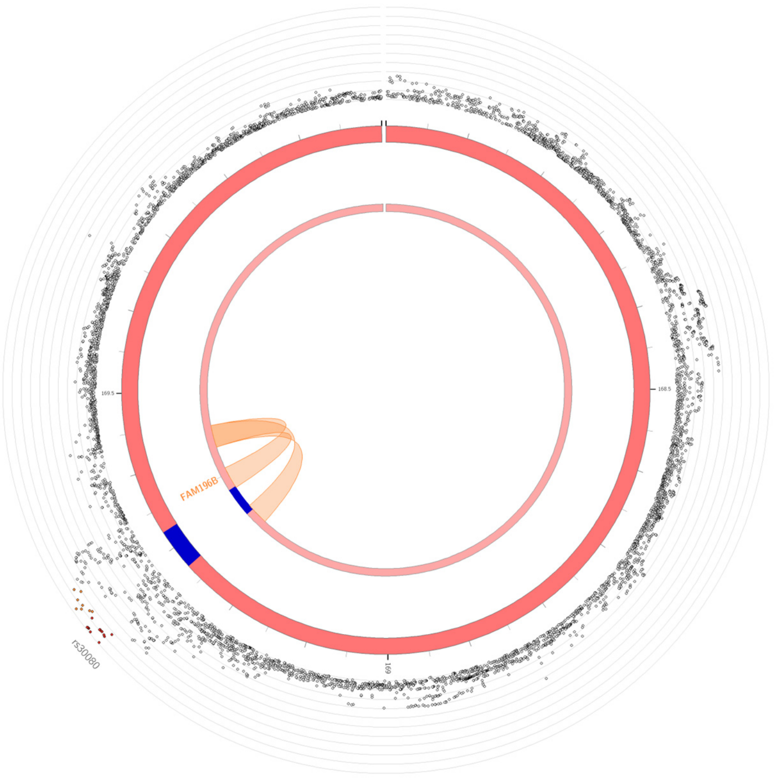

Chromosome 5

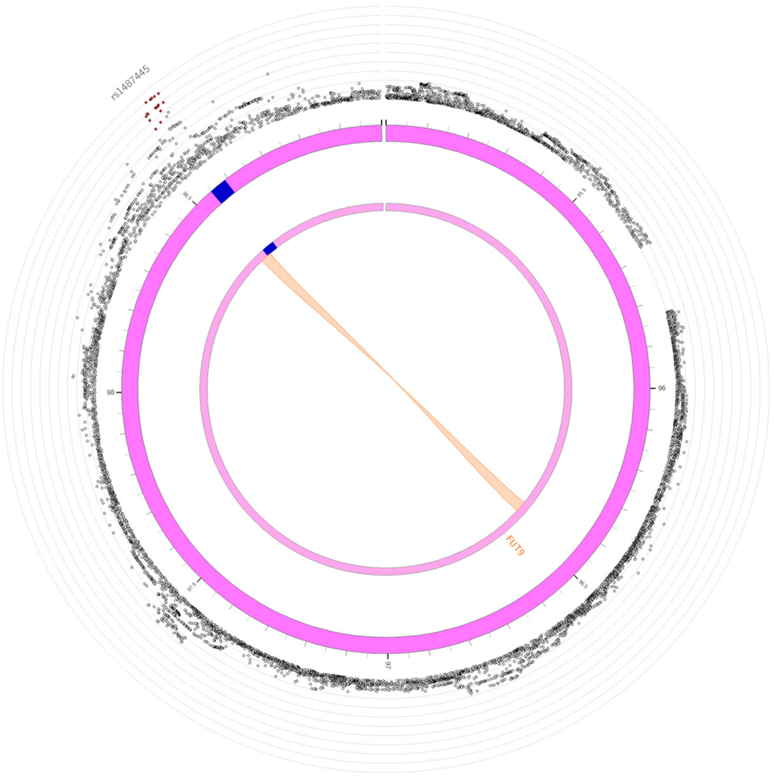

Chromosome 6

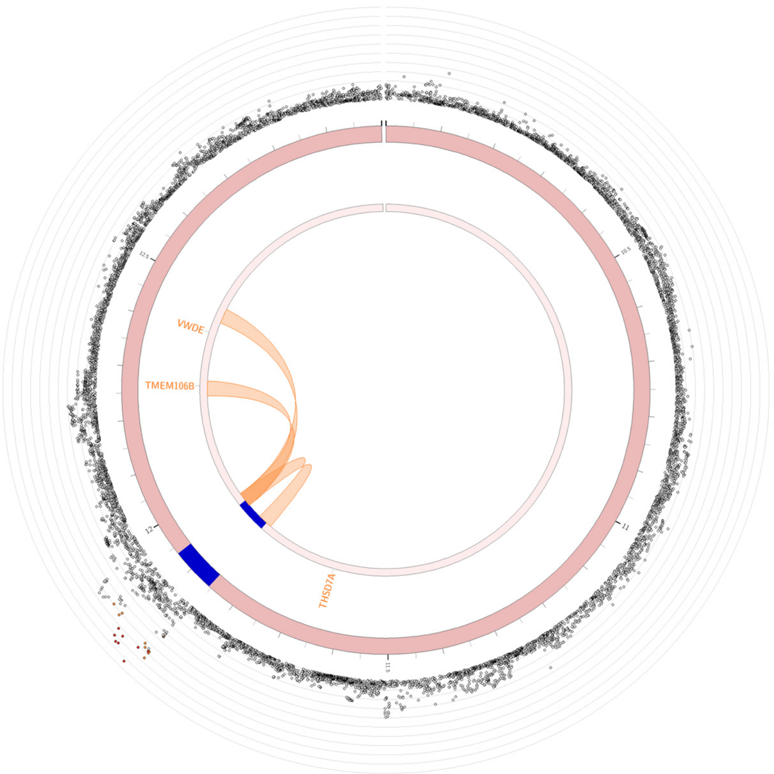

Chromosome 7

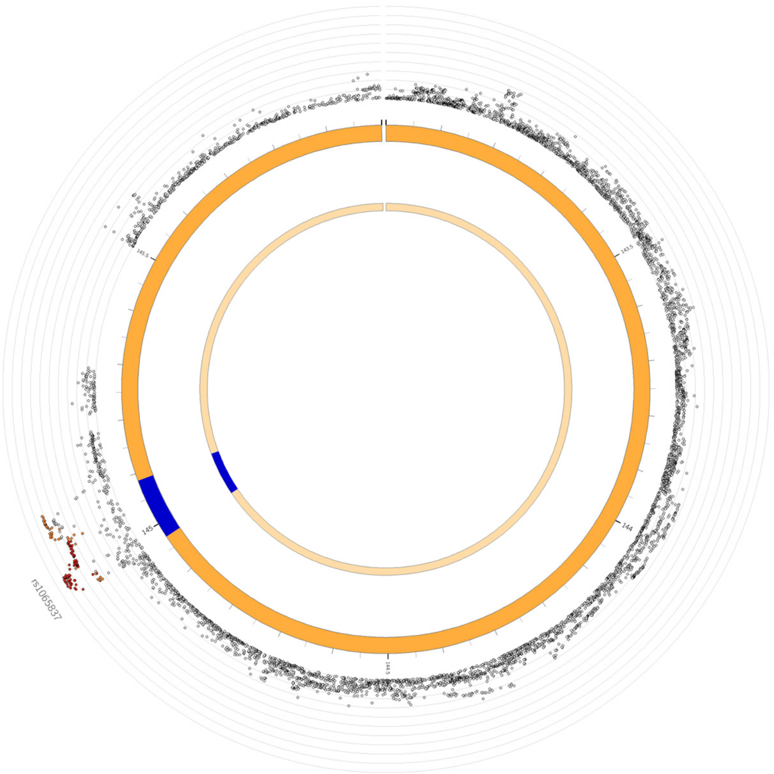

Chromosome 8

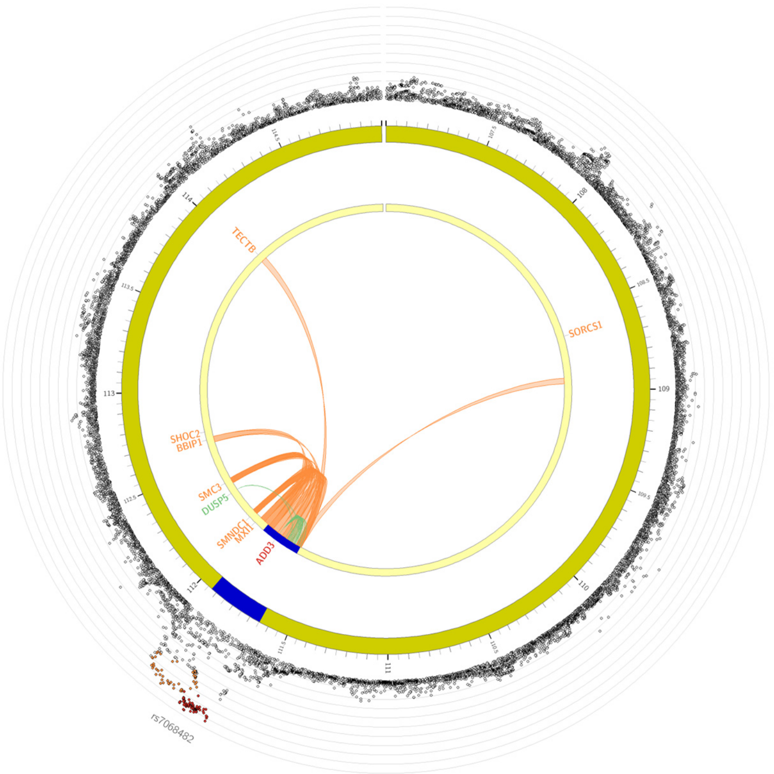

Chromosome 10

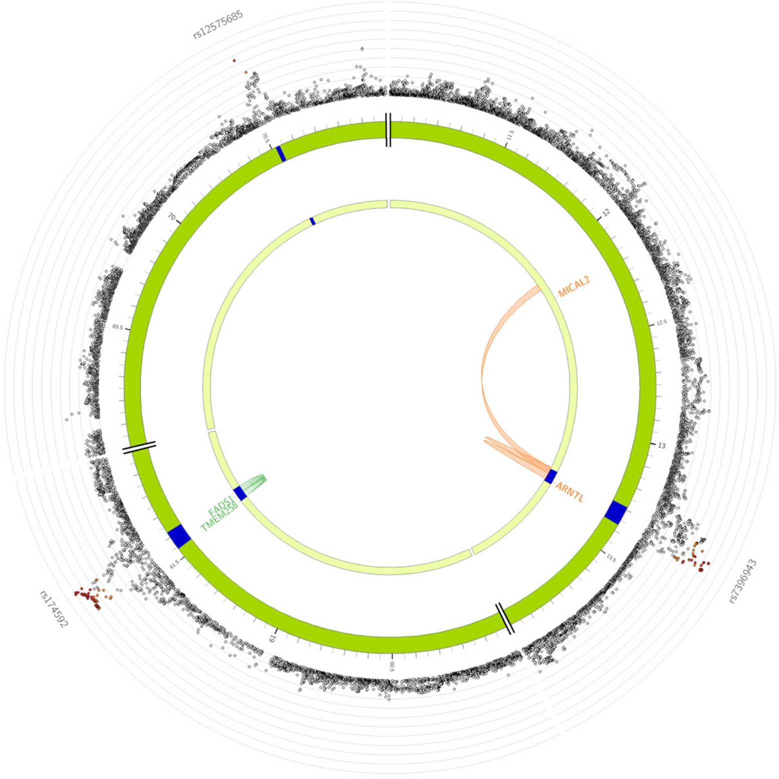

Chromosome 11

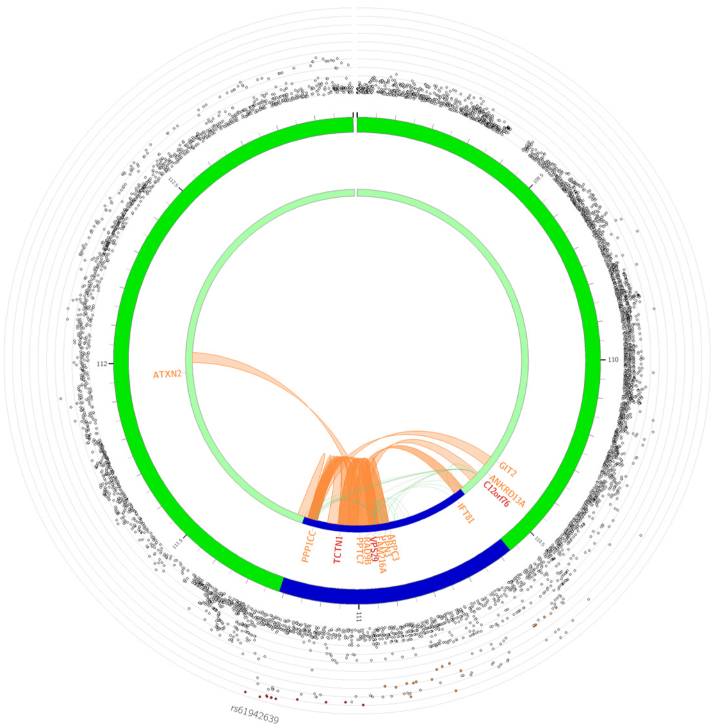

Chromosome 12

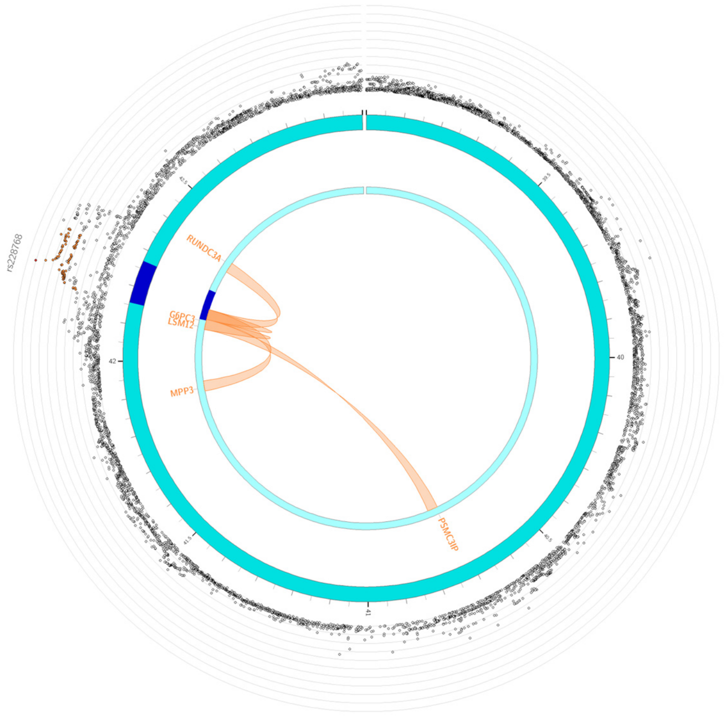

Chromosome 17

Supplementary Figure 38. Significant SNPs identified in BD Independent GWAS

The chromosome is depicted as a circle with SNPs plotted by their -log10(p-value), and lead SNPs annotated. Orange links indicate chromatin contact, green links indicate cis-eQTLs, and red links indicate both.

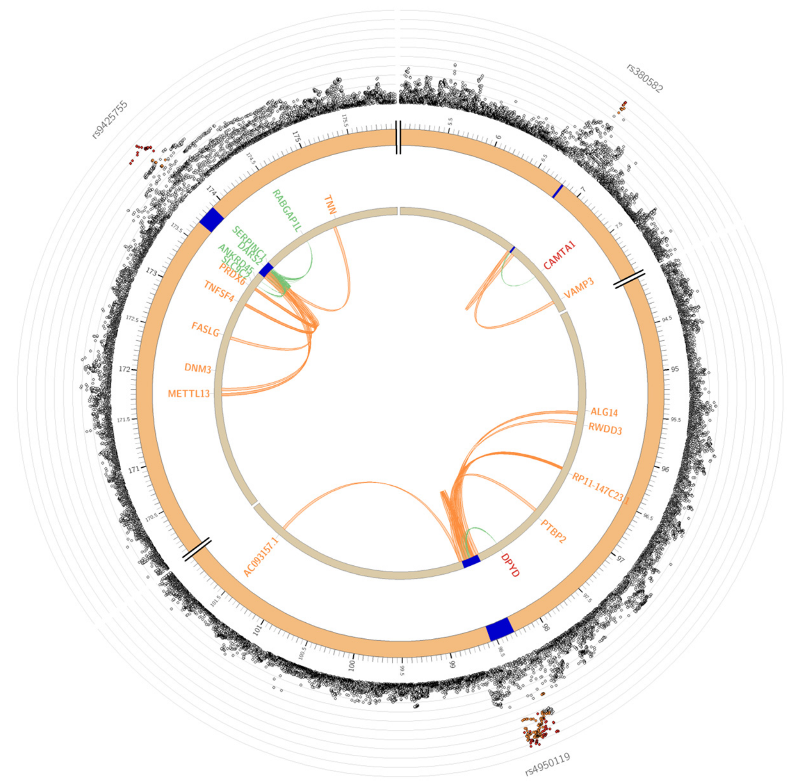

Chromosome 1

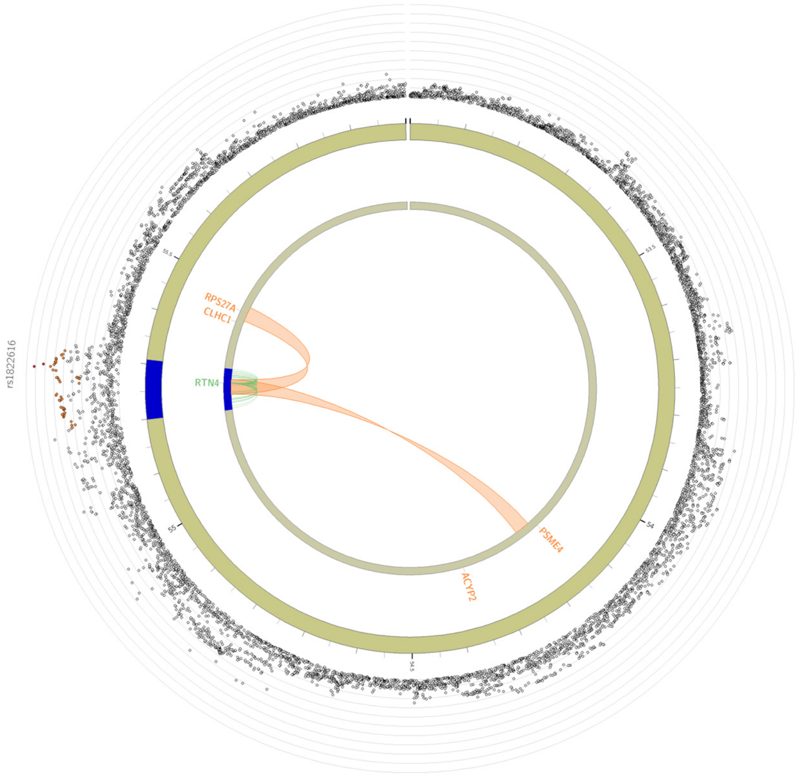

Chromosome 2

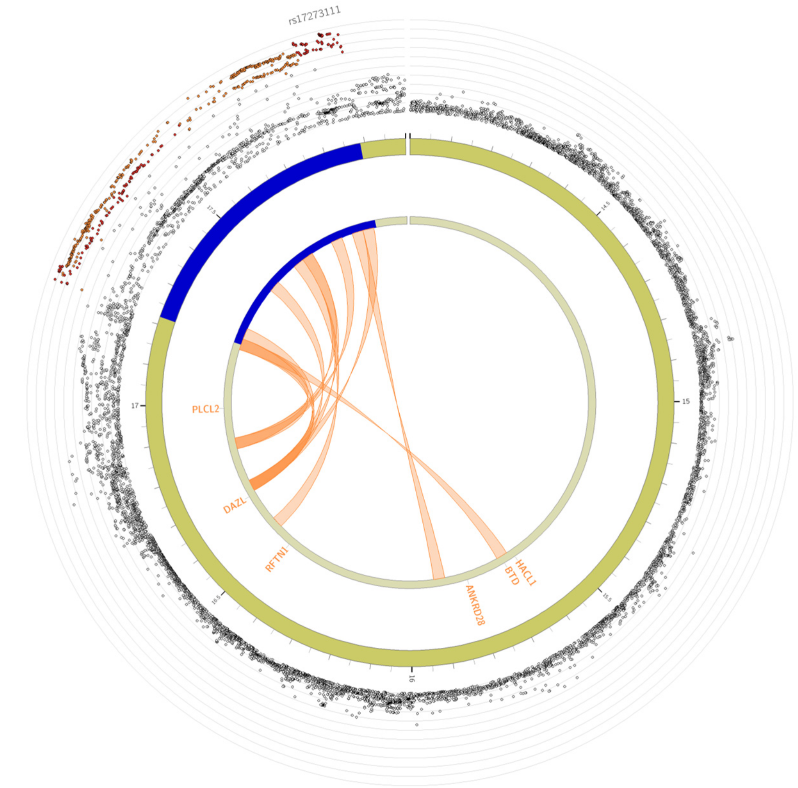

Chromosome 3

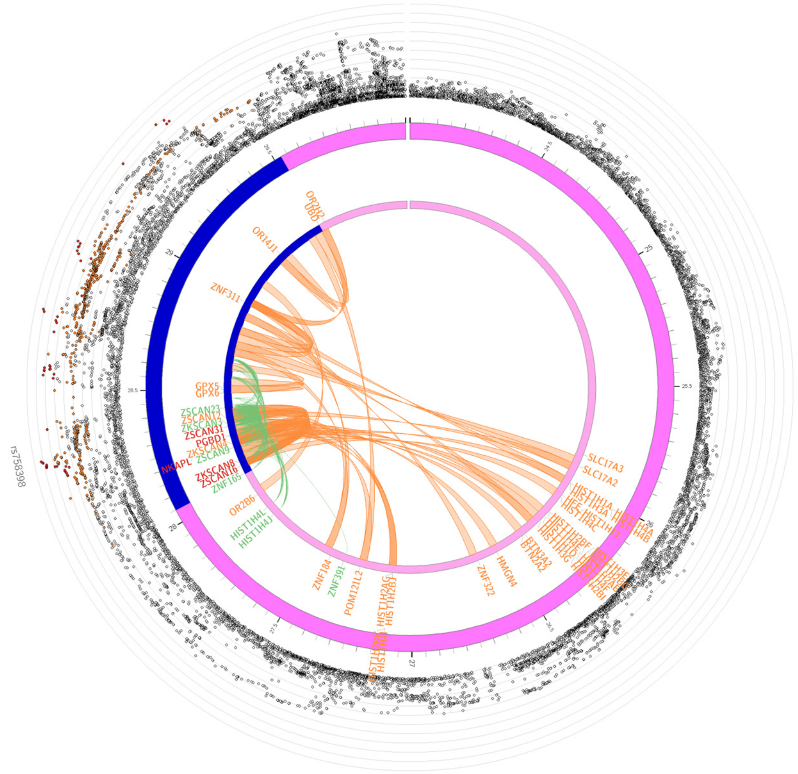

Chromosome 6

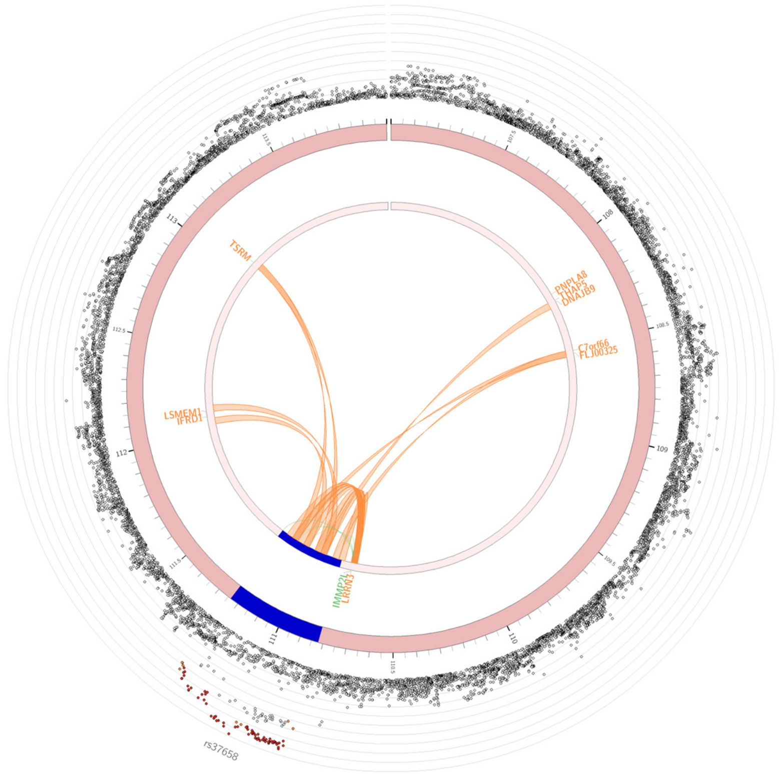

Chromosome 7

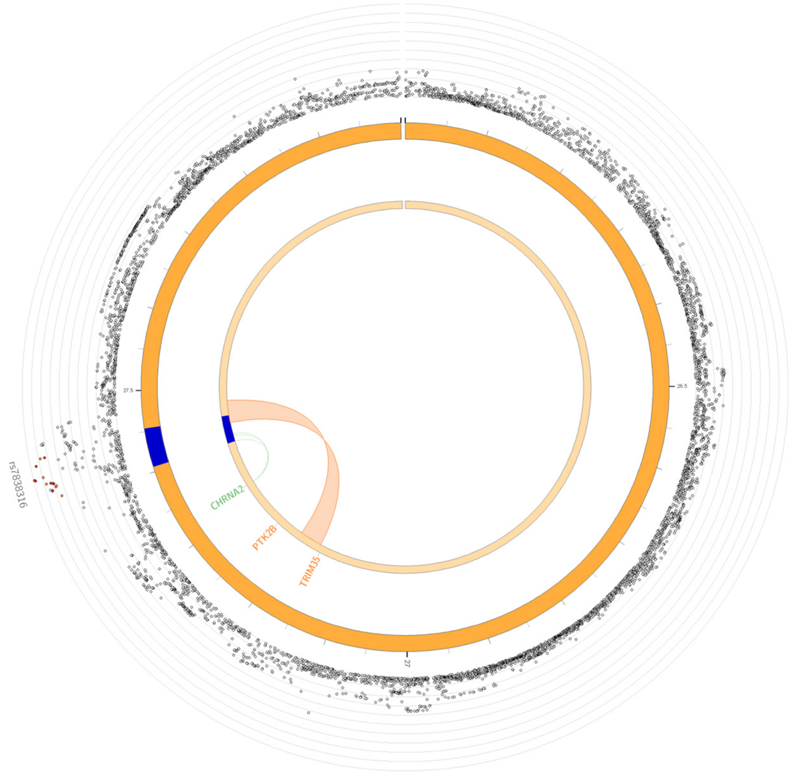

Chromosome 8

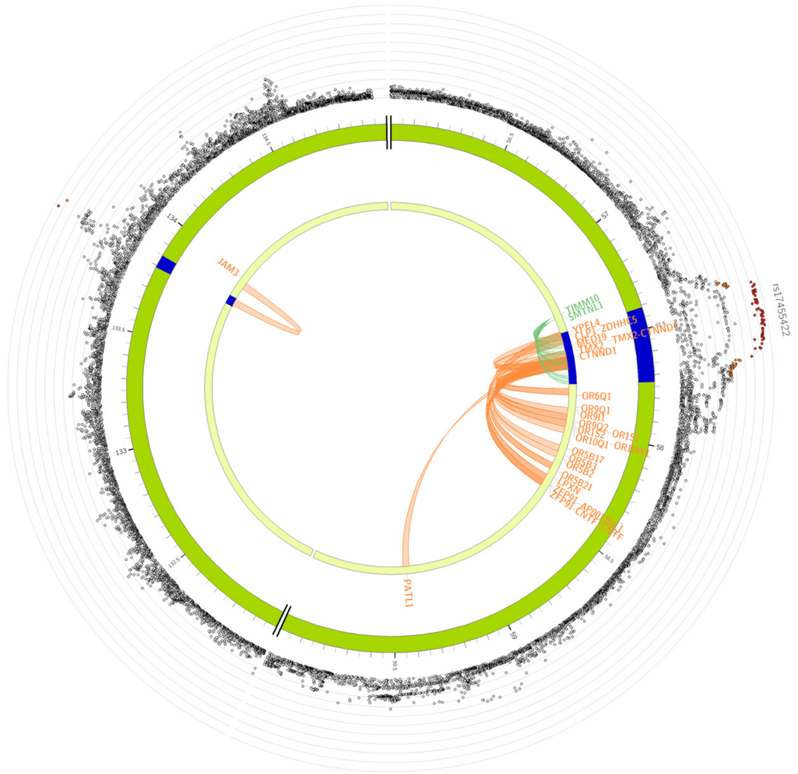

Chromosome 11

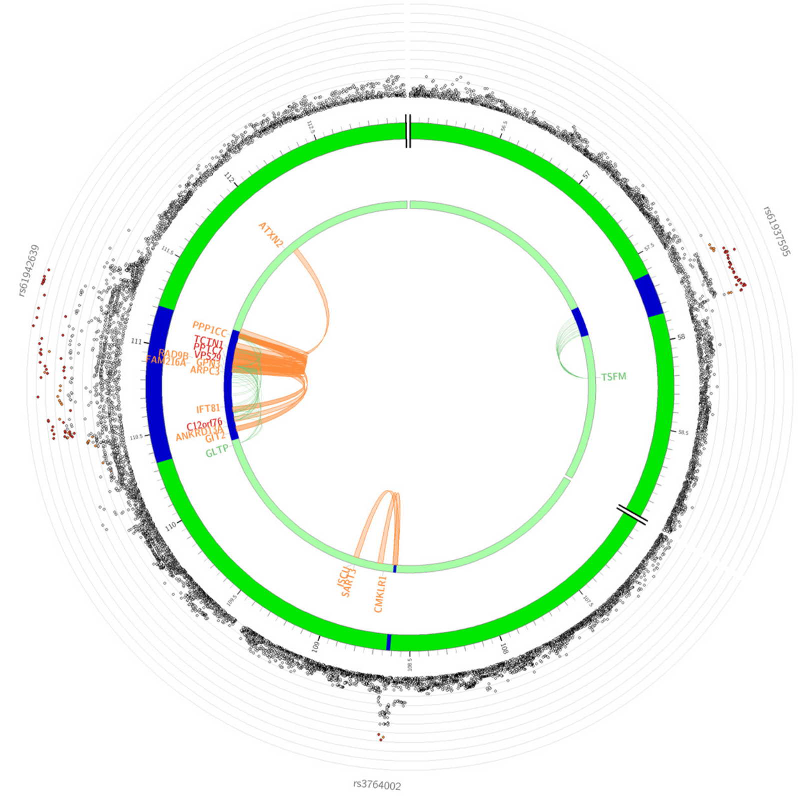

Chromosome 12

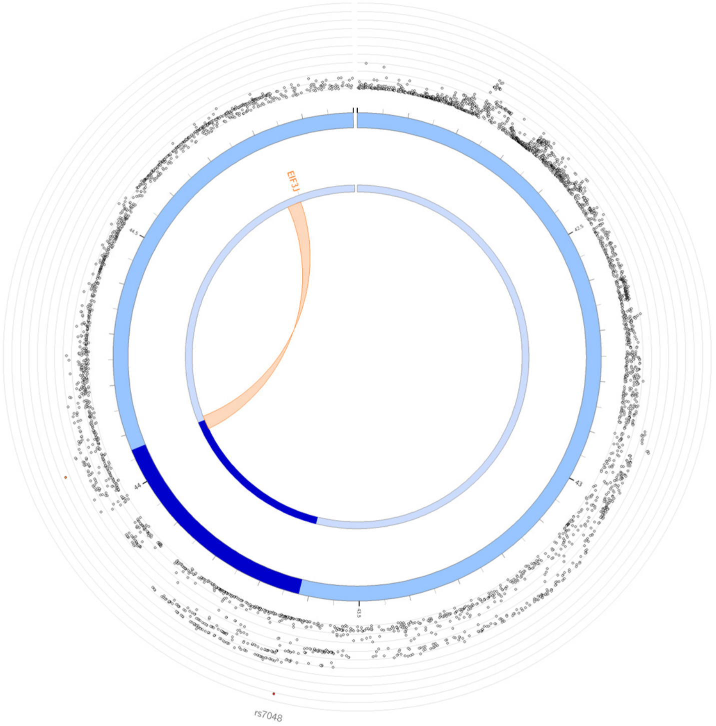

Chromosome 15

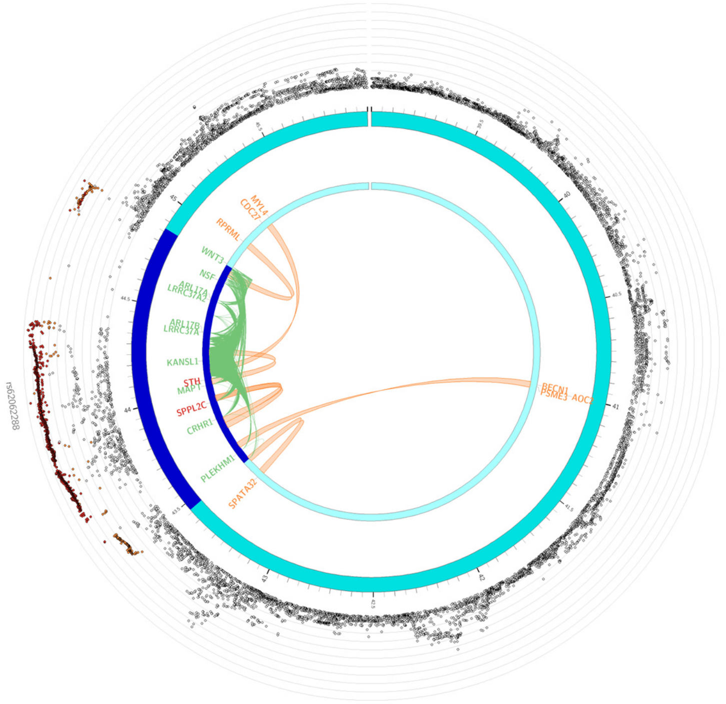

Chromosome 17

Supplementary Figure 39. Significant SNPs identified in SCZ Independent GWAS
The chromosome is depicted as a circle with SNPs plotted by their -log10(p-value), and lead SNPs annotated. Orange links indicate chromatin contact, green links indicate cis-eQTLs, and red links indicate both.

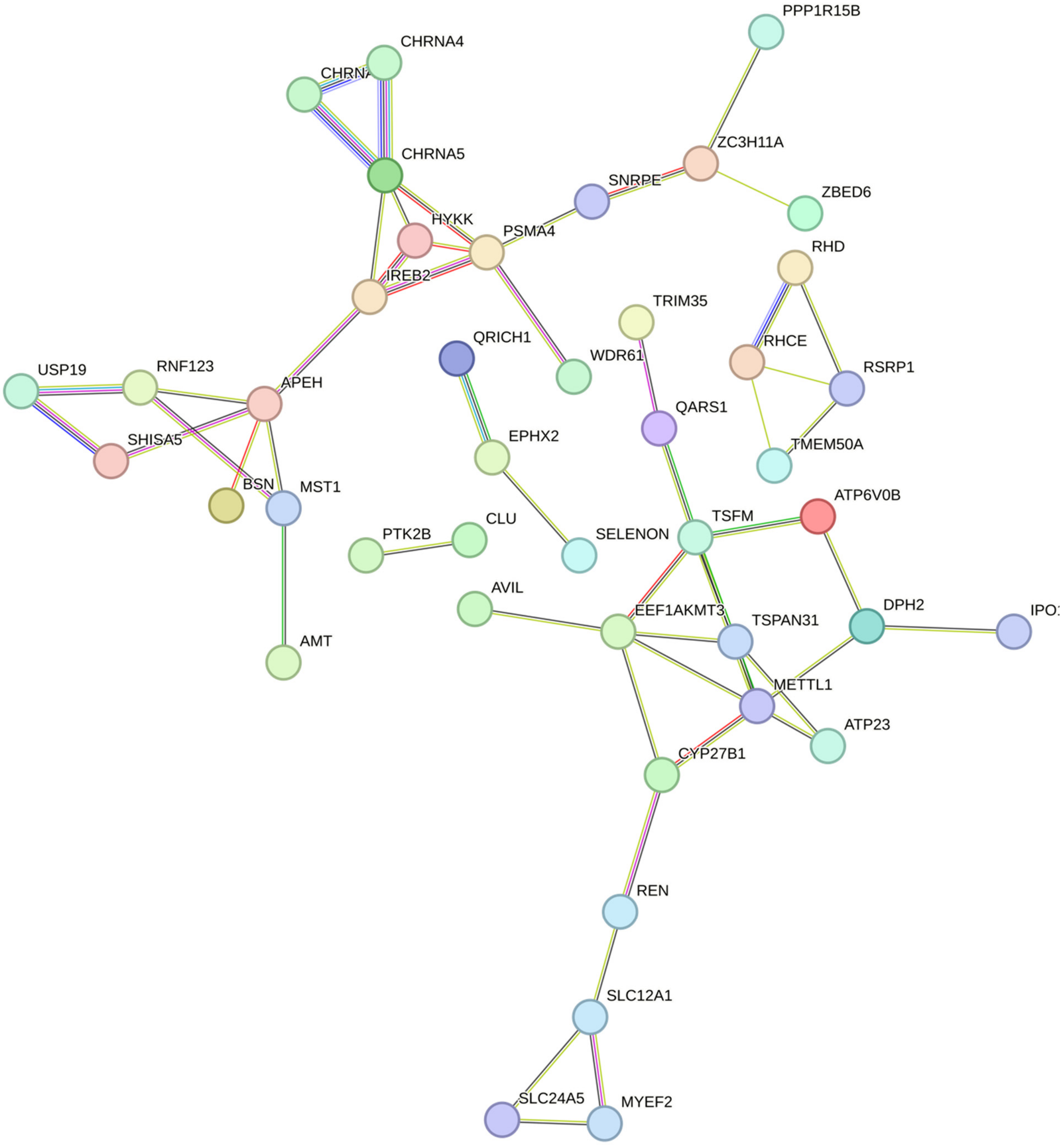

Supplementary Figure 40. Protein-protein interaction network plot for TUD Independent. Network plot was generated using STRING database v12.0. Nodes represent proteins, and edges represent protein-protein associations. Blue and pink edges represent known interactions, while green, red, and blue represent predicted interactions.

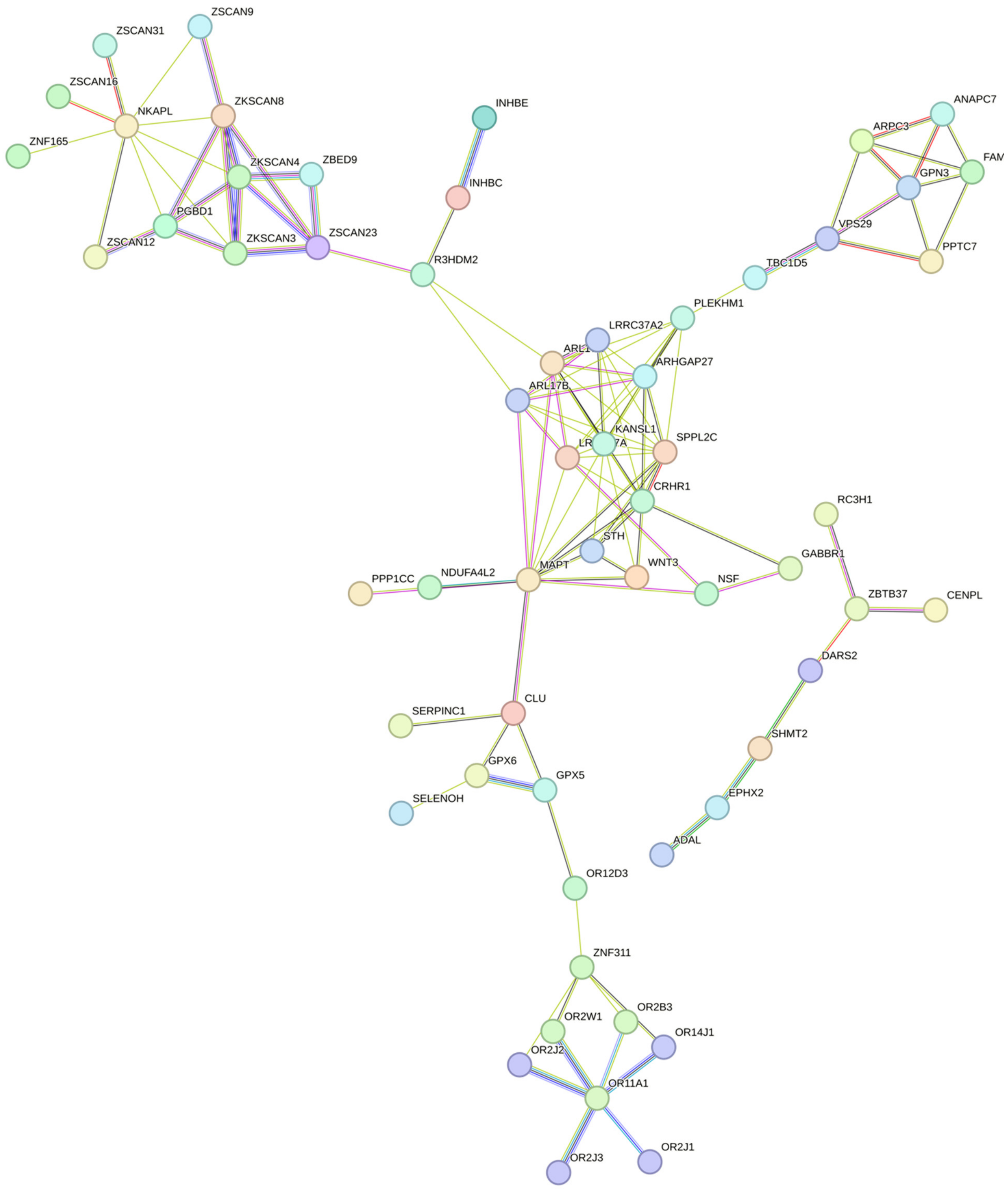

Supplementary Figure 41. Protein-protein interaction network plot for SCZ Independent. Network plot was generated using STRING database v12.0. Nodes represent proteins, and edges represent protein-protein associations. Blue and pink edges represent known interactions, while green, red, and blue represent predicted interactions.

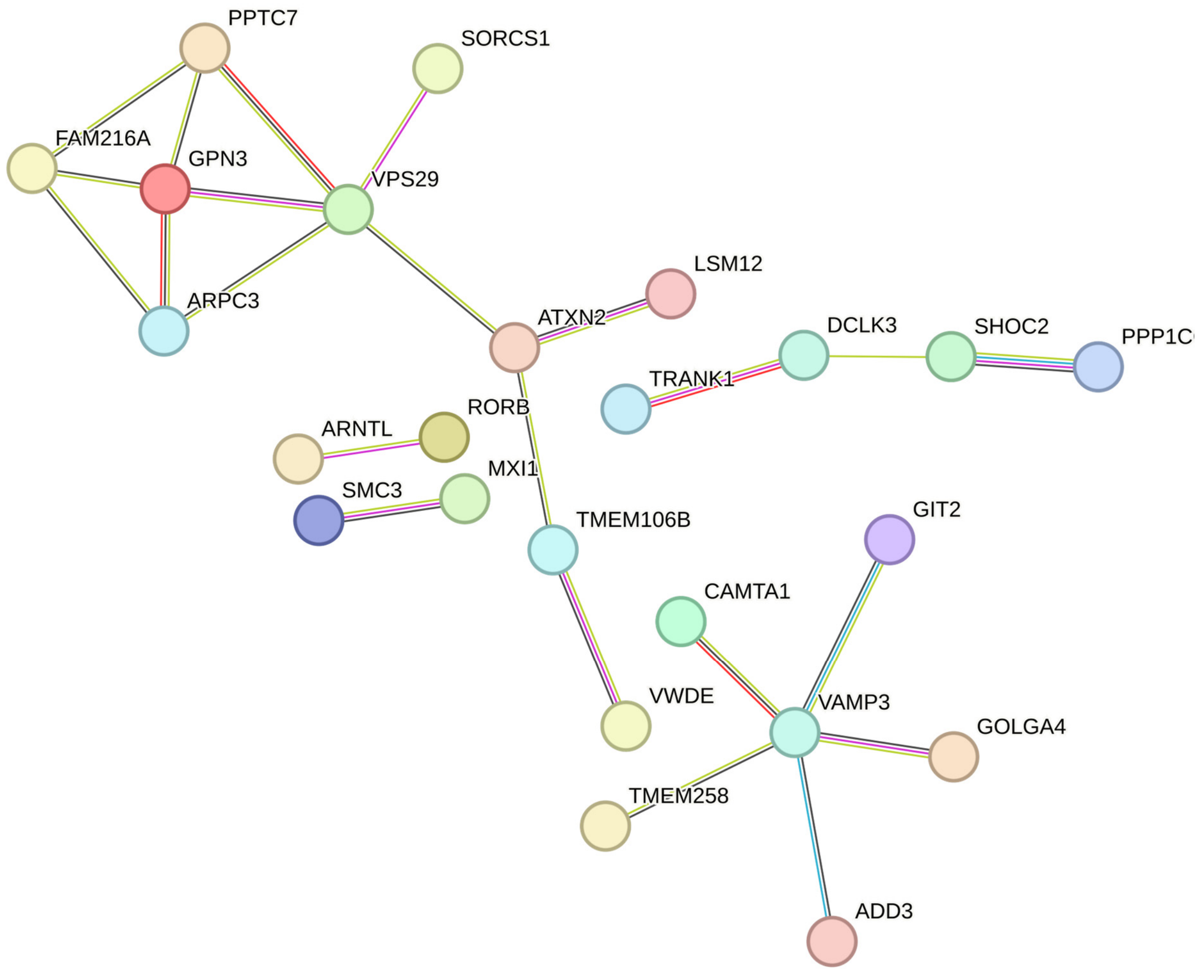

Supplementary Figure 42. Protein-protein interaction network plot for BD Independent. Network plot was generated using STRING database v12.0. Nodes represent proteins, and edges represent protein-protein associations. Blue and pink edges represent known interactions, while green, red, and blue represent predicted interactions.

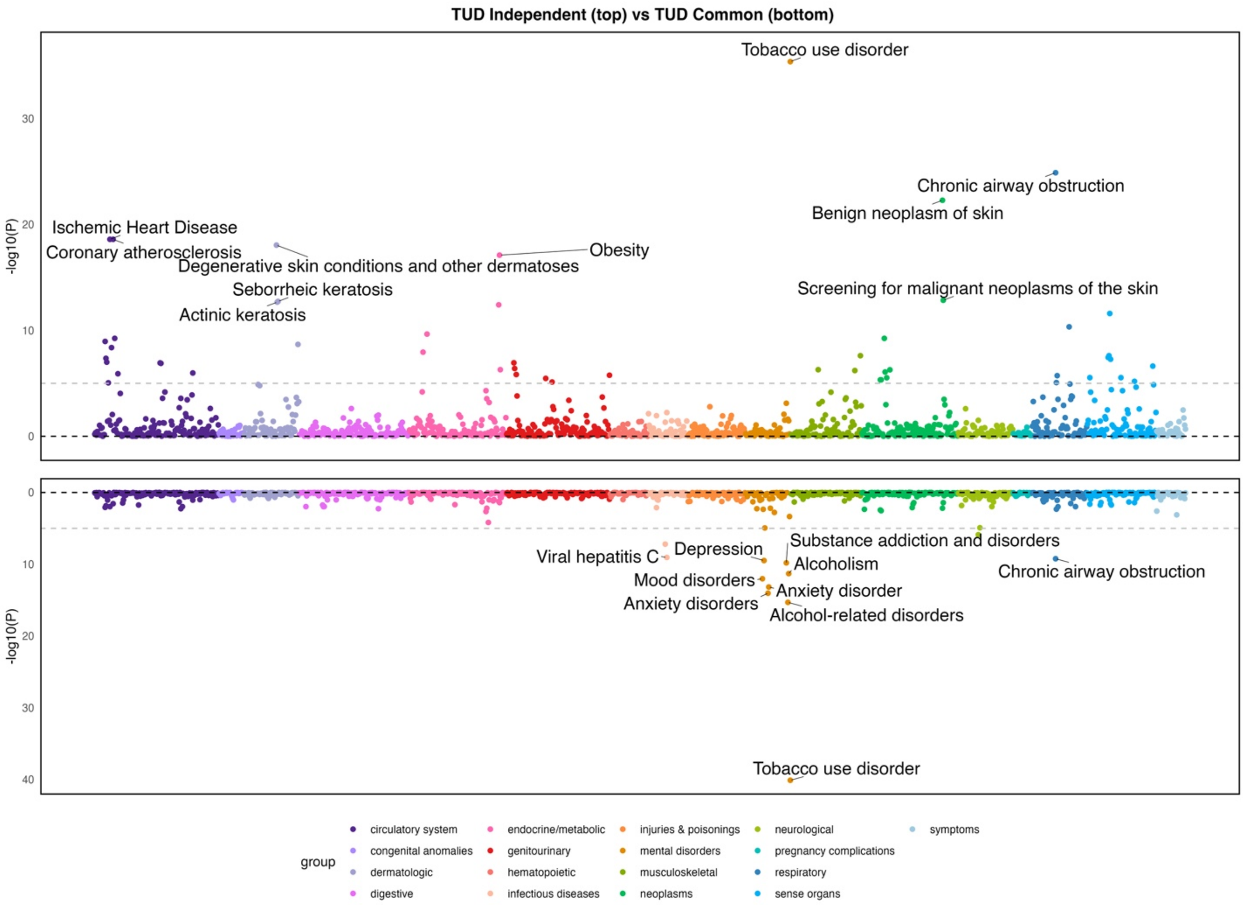

Supplementary Figure 43. Hudson plot of PheWAS results for tobacco use disorders GWAS-by-subtraction in Penn Medicine BioBank

The top 10 associations for each GWAS are shown (top panel = TUD Independent, bottom panel = TUD Common). All p-values were adjusted using Benjamini-Hochberg false discovery rate (FDR) correction.

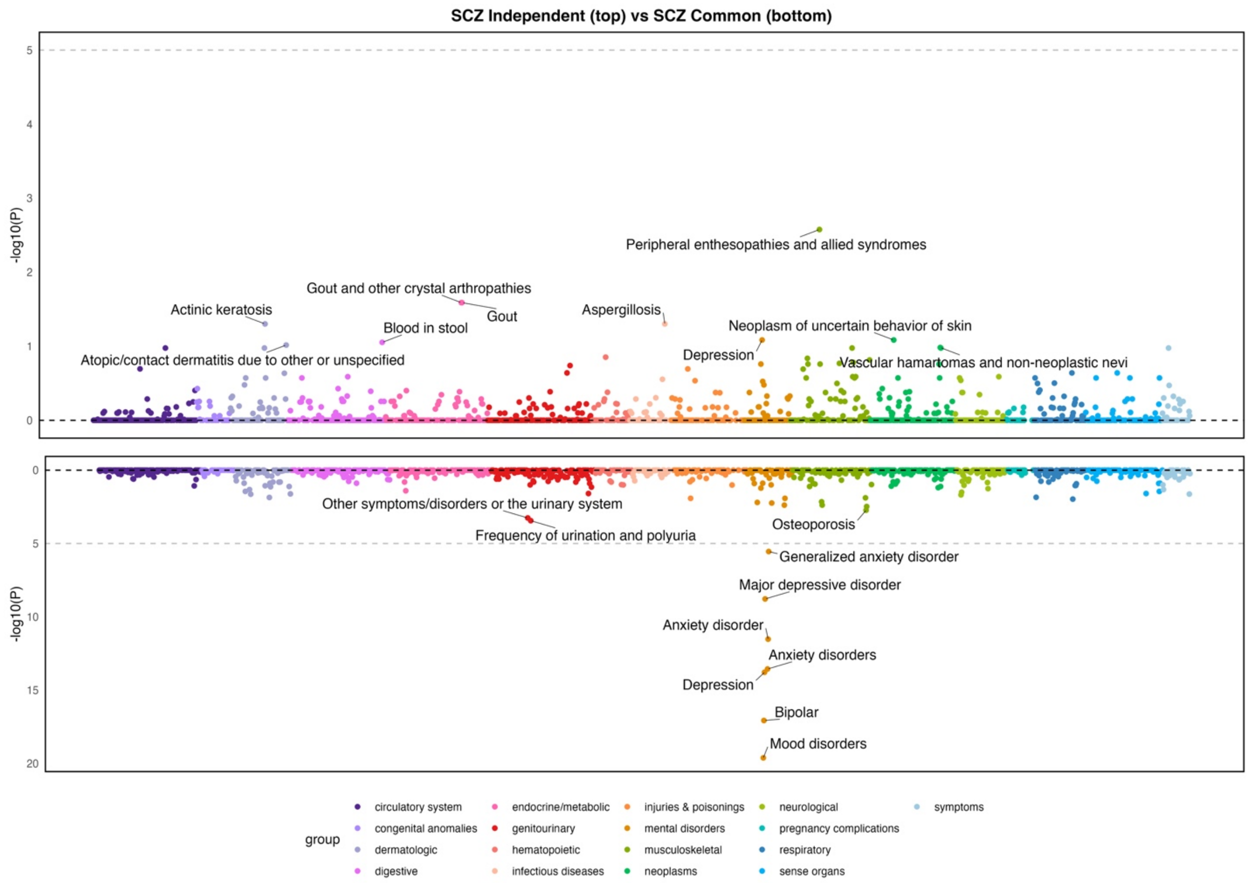

Supplementary Figure 44. Hudson plot of PheWAS results for schizophrenia GWAS-by-subtraction in Penn Medicine BioBank

The top 10 associations for each GWAS are shown (top panel = SCZ Independent, bottom panel = SCZ Common). All p-values were adjusted using Benjamini-Hochberg false discovery rate (FDR) correction.

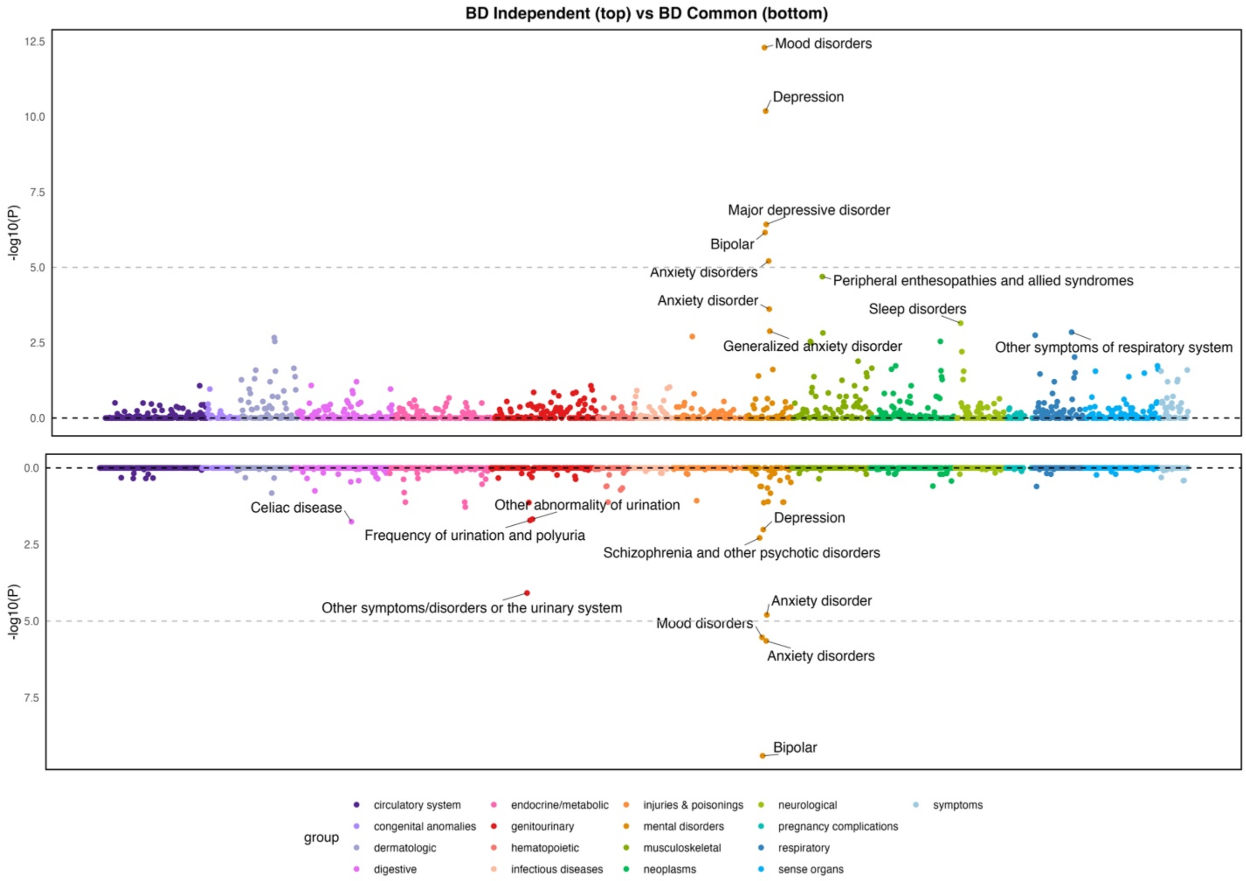

Supplementary Figure 45. Hudson plot of PheWAS results for bipolar disorder GWAS-by-subtraction in Penn Medicine BioBank

The top 10 associations for each GWAS are shown (top panel = BD Independent, bottom panel = BD Common). All p-values were adjusted using Benjamini-Hochberg false discovery rate (FDR) correction.

References

1. Turley P, Walters RK, Maghzian O, Okbay A, Lee JJ, Fontana MA, et al. (2018): Multi-trait analysis of genome-wide association summary statistics using MTAG. *Nature Genetics*. 50:229-237.
2. Purves KL, Coleman JRI, Meier SM, Rayner C, Davis KAS, Cheesman R, et al. (2020): A major role for common genetic variation in anxiety disorders. *Molecular Psychiatry*. 25:3292-3303.
3. Levey DF, Gelernter J, Polimanti R, Zhou H, Cheng Z, Aslan M, et al. (2020): Reproducible Genetic Risk Loci for Anxiety: Results From ~200,000 Participants in the Million Veteran Program. *American Journal of Psychiatry*. 177:223-232.
4. Otowa T, Hek K, Lee M, Byrne EM, Mirza SS, Nivard MG, et al. (2016): Meta-analysis of genome-wide association studies of anxiety disorders. *Molecular Psychiatry*. 21:1391-1399.
5. Grotzinger AD, Fuente Jdl, Privé F, Nivard MG, Tucker-Drob EM (2023): Pervasive Downward Bias in Estimates of Liability-Scale Heritability in Genome-wide Association Study Meta-analysis: A Simple Solution. *Biological Psychiatry*. 93:29-36.
6. Grotzinger AD, Rhemtulla M, de Vlaming R, Ritchie SJ, Mallard TT, Hill WD, et al. (2019): Genomic structural equation modelling provides insights into the multivariate genetic architecture of complex traits. *Nature Human Behaviour*. 3:513-525.
7. The International HapMap 3 Consortium (2010): Integrating common and rare genetic variation in diverse human populations. *Nature*. 467:52-58.
8. The 1000 Genomes Project Consortium (2015): A global reference for human genetic variation. *Nature*. 526:68-74.
9. Pan-UKB Team (2020): https://pan.ukbb.broadinstitute.org.
10. Luo Y, Li X, Wang X, Gazal S, Mercader JM, Me Research T, et al. (2021): Estimating heritability and its enrichment in tissue-specific gene sets in admixed populations. *Human Molecular Genetics*. 30:1521-1534.
11. Finucane HK, Bulik-Sullivan B, Gusev A, Trynka G, Reshef Y, Loh P-R, et al. (2015): Partitioning heritability by functional annotation using genome-wide association summary statistics. *Nature Genetics*. 47:1228-1235.
12. Ni G, Moser G, Ripke S, Neale BM, Corvin A, Walters JTR, et al. (2018): Estimation of Genetic Correlation via Linkage Disequilibrium Score Regression and Genomic Restricted Maximum Likelihood. *The American Journal of Human Genetics*. 102:1185-1194.
13. West SG, Taylor AB, Wu W (2012): Model fit and model selection in structural equation modeling. *Handbook of structural equation modeling*. New York, NY, US: The Guilford Press, pp 209-231.
14. Loehlin JC (1996): The Cholesky approach: A cautionary note. *Behavior Genetics*. 26:65-69.
15. Purcell S, Neale B, Todd-Brown K, Thomas L, Ferreira MA, Bender D, et al. (2007): PLINK: a tool set for whole-genome association and population-based linkage analyses. *Am J Hum Genet*. 81:559-575.
16. Sollis E, Mosaku A, Abid A, Buniello A, Cerezo M, Gil L, et al. (2023): The NHGRI-EBI GWAS Catalog: knowledgebase and deposition resource. *Nucleic Acids Research*. 51:D977-D985.
17. Hinrichs AS, Karolchik D, Baertsch R, Barber GP, Bejerano G, Clawson H, et al. (2006): The UCSC Genome Browser Database: update 2006. *Nucleic Acids Research*. 34:D590-D598.

18. Watanabe K, Stringer S, Frei O, Umićević Mirkov M, de Leeuw C, Polderman TJC, et al. (2019): A global overview of pleiotropy and genetic architecture in complex traits. *Nature Genetics*. 51:1339-1348.
19. Cuéllar-Partida G, Lundberg M, Kho PF, D'Urso S, Gutiérrez-Mondragón LF, Ngo TT, et al. (2019): Complex-Traits Genetics Virtual Lab: A community-driven web platform for post-GWAS analyses. *bioRxiv*.518027.
20. Denny JC, Bastarache L, Ritchie MD, Carroll RJ, Zink R, Mosley JD, et al. (2013): Systematic comparison of phenome-wide association study of electronic medical record data and genome-wide association study data. *Nature Biotechnology*. 31:1102-1111.
21. Verma A, Damrauer SM, Naseer N, Weaver J, Kripke CM, Guare L, et al. (2022): The Penn Medicine BioBank: Towards a Genomics-Enabled Learning Healthcare System to Accelerate Precision Medicine in a Diverse Population. *Journal of Personalized Medicine*. 12:1974.
22. Jöreskog KG (1969): A general approach to confirmatory maximum likelihood factor analysis. *Psychometrika*. 34:183-202.
23. Li Y, Wen Z, Hau K-T, Yuan K-H, Peng Y (2020): Effects of Cross-loadings on Determining the Number of Factors to Retain. *Structural Equation Modeling: A Multidisciplinary Journal*. 27:841-863.
24. Hatoum AS, Johnson EC, Colbert SMC, Polimanti R, Zhou H, Walters RK, et al. (2022): The addiction risk factor: A unitary genetic vulnerability characterizes substance use disorders and their associations with common correlates. *Neuropsychopharmacology*. 47:1739-1745.
25. Araujo DJ, Toriumi K, Escamilla CO, Kulkarni A, Anderson AG, Harper M, et al. (2017): Foxp1 in Forebrain Pyramidal Neurons Controls Gene Expression Required for Spatial Learning and Synaptic Plasticity. *The Journal of Neuroscience*. 37:10917-10931.
26. Braccioli L, Vervoort SJ, Adolfs Y, Heijnen CJ, Basak O, Pasterkamp RJ, et al. (2017): FOXP1 Promotes Embryonic Neural Stem Cell Differentiation by Repressing Jagged1 Expression. *Stem Cell Reports*. 9:1530-1545.
27. Ning Z, Liu K, Xiong H (2021): Roles of BTLA in Immunity and Immune Disorders. *Frontiers in Immunology*. 12.
28. Wang XD, Su YA, Wagner KV, Avrabos C, Scharf SH, Hartmann J, et al. (2013): Nectin-3 links CRHR1 signaling to stress-induced memory deficits and spine loss. *Nat Neurosci*. 16:706-713.
29. Wang HL, Li JT, Wang H, Sun YX, Liu R, Wang XD, et al. (2020): Prefrontal Nectin3 Reduction Mediates Adolescent Stress-Induced Deficits of Social Memory, Spatial Working Memory, and Dendritic Structure in Mice. *Neurosci Bull*. 36:860-874.
30. van der Kooij MA, Fantin M, Rejmak E, Grosse J, Zanoletti O, Fournier C, et al. (2014): Role for MMP-9 in stress-induced downregulation of nectin-3 in hippocampal CA1 and associated behavioural alterations. *Nature Communications*. 5:4995.
